# Lexico-Semantic Influence on Syntactic Processing: An Eye-Tracking Study with Spanish Relative Clauses

**DOI:** 10.3390/brainsci13030409

**Published:** 2023-02-26

**Authors:** Esther Álvarez-García, José Manuel Igoa González

**Affiliations:** 1 Department of Hispanic and Classic Philology, University of León, 24007 León, Spain; 2 Department of Basic Psychology, Autonomous University of Madrid, 28049 Madrid, Spain

**Keywords:** language comprehension, Spanish relative clause, eye-tracking, interactive accounts

## Abstract

This paper investigates the interaction between lexicosemantic and syntactic information in sentence processing by examining the online comprehension of Spanish relative clauses (RCs) of both restrictive and non-restrictive types. A corpus study shows that, in Spanish, a RC may be introduced by different function words (called relativizers), which differ in lexical frequency, as well as semantic features. Based on these facts, an eye-tracking experiment was conducted with the aim of analyzing whether lexicosemantic information could influence sentence processing at the early stages. The results report an early influence of lexicosemantic information not only when activating a relativizer but also when integrating it within the syntactic structure. Additionally, the semantic role played by each RC type seems to constrain sentence processing at different regions. Our results favor an interactive view of language processing, according to which language comprehension is guided from the early stages by different kinds of linguistic information rather than syntactic information alone.

## 1. Introduction

One of the main goals in the study of human language processing is to understand how listeners/readers recover the syntactic structure of a sentence from a string of words in order to comprehend the intended message. Assuming that structure building operations (known as parsing) are necessary for sentence understanding, it is pertinent to ask what kinds of information come into play in syntactic processing and when each one of them is accessed or becomes activated along the process. A general distinction in this regard is often made between purely structural information, on the one hand, involving word class identification and labelling and phrase structure assembly, and non-syntactic information, on the other, which encompasses a wide range of information sources such as different properties of individual lexical items, e.g., frequency of use, relevant semantic features such as animacy or thematic roles, or contextual information from previous discourse or from a visual scenario [1].

As regards the order in which the various relevant information types come into play, the issue is whether syntactic decisions are made, or at least initiated, before any extra-syntactic information becomes available, or are constrained or guided by this information in the first place. The latter may occur in two different ways, either in a cascaded fashion, according to which non-syntactic information is activated alongside syntactic information and may be used to decide between alternative parsing choices at a given point [2], or in a fully interactive way, where non-syntactic information favors certain parsing options and blocks others incompatible with it [3].

A related question that is often addressed in the literature on sentence parsing refers to the ways in which syntactic and non-syntactic information are used at different stages, either separately or in combination, and sequentially or simultaneously. This involves, on the one hand, the pace at which different parsing operations take place, either in the form of a continuous, incremental word-by word progression [4], or in a discontinuous manner, with some points in the sentence involving more costly operations and others allowing a more easy-going processing mode, depending on the memory requirements the parser faces at each point [5], and relatedly, the depth of processing attained by the parser in the face of different strategies, such as performing a shallower syntactic analysis with few initial commitments until enough information becomes available to carry out a more fine-grained analysis if or when needed [6,7].

The major aim of this article is to assess the interplay of syntactic and non-syntactic information during the comprehension of relative clauses (henceforth RCs) in Spanish. To that end, we will report a corpus study followed by an eye-tracking experiment carried out with two types of RCs, restrictive and non-restrictive, in which we manipulated (or recorded, in the case of the corpus study) the kind of relativizer that introduces the RC, taking advantage of the fact that Spanish displays interesting contrasts between different kinds of relativizers. Besides the default and more common item *que* (that stands for “that” in English), which lacks semantic features, Spanish has other less frequent relativizers, one of which carries gender and number features (i.e., *el cual*, “which” in English), and others that bear semantic features as well, such as *quien* (“who/whom”) or *donde* (“where”), which respectively encode animacy and location information. By addressing the effects of these two contrasts—RC type and relativizer type—on RC processing, we purport to find out whether non-syntactic information—i.e., structural or lexical frequency and/or semantic features—is recruited alongside purely syntactic information at different stages during sentence processing. Although RC processing has been widely studied in psycholinguistic research across many different languages and for various purposes, our study is the first to test the role of these variables in function (closed class) words.

## 2. Literature Review

### 2.1. RC Processing

Relative clauses have been of interest in the psycholinguistic research for different reasons: on the one hand, they can be manipulated to examine the resolution of local structural ambiguities (such as “The defendant examined by the lawyer turned out to be unreliable/The defendant that was examined…” [8]), but more importantly for the purpose of this paper, they allow us to analyze structure building operations and the influence of different kinds of information—both linguistic and non-linguistic—on these processes. In this regard, two main topics related to RC processing have been examined.

On the one hand, several studies have analyzed RC processing when these structures follow a complex noun phrase (NP), so the RC—between square brackets in (1)—may modify the first noun, as in (1a), thus producing a “high attachment” of the RC, or the second noun, as in (1b), resulting in a “low attachment” of the RC [9].

(1)

a. Someone shot the maid of the actor [who was on the balcony with her husband].
b. Someone shot the butler of the actress [who was on the balcony with her husband].

English speakers show a preference for a low attachment of the RC, reflected in fewer comprehension errors and shorter reading times when compared to a forced high attachment structure (among others, [9,10,11,12,13]). These results have been interpreted as favoring modular and locality theories of language processing, such as the garden-path model [14,15], which claims that sentence parsing depends on purely structural information, at least at early stages, and always favors an analysis that minimizes processing load. In this sense, a low attachment of the RC implies that the incoming constituent (that is, the RC) is associated with the currently processed phrase, resulting in a less demanding operation when compared to the attachment to a phrase that has already been processed and therefore closed. This locality preference is implemented in the garden-path model through a syntactic principle called “Late Closure” [16], which does not take into account non-syntactic information related to the sentence that is being processed.

Low attachment preferences have been replicated across different languages—for instance, Swedish, Norwegian, Romanian [17], or Chinese [18]; however, there are also languages that exhibit a different pattern. This is the case of Spanish, the language under study in this paper. Cuetos & Mitchell [9] first showed that Spanish speakers favor a high attachment of the RC (also [11,12,19,20]), which speaks against locality and, therefore, against syntactic principles exclusively guiding sentence processing. This preference has also been confirmed in other languages such as Dutch [21] or German [22].

In addition, some studies have also proved that attachment preferences might be influenced by non-syntactic information, such as the semantic features of the NPs (e.g., Cuetos & Mitchell [9] analyzed English RCs following non-human NPs, resulting in a preference for a high attachment of the RC), the preposition linking the two nouns of the complex NP (e.g., Gilboy et al. [23] found that replacing the Spanish preposition *de* (“of”) with *con* (“with”) results in a preference shift from high to low attachment), or prosody (e.g., Maynell [24] showed that the presence of an intonational phrase boundary before an English RC results in a preference for a high attachment). These results seem to refute modular proposals of language processing and, instead, favor an interactive view where extra-syntactic information becomes available at early stages and can even guide sentence processing [3,25].

The second main topic in the psycholinguistic literature related to RC processing has been the study of subject versus object RCs (henceforth SRC versus ORC). SRCs, that is, RCs where the relative pronoun (or relativizer) takes the subject role, as in (2a), seem to be less costly to process in languages such as English when compared to ORCs (2b), where the relativizer is the object of the relative verb—*attacked* in (2).

(2)

a. The reporter [that_i_ __i_ attacked the senator] admitted the error.
b. The reporter [that_i_ the senator attacked __i_] admitted the error.

This asymmetry has been replicated across different languages—for instance, German [26], French [27], or Japanese [28], and with different experimental paradigms and measures—self-paced reading [29], eye-tracking [30,31], ERPs [32], and fMRI [33], which was initially interpreted as evidence of a universal processing mode guided by syntactic principles, such as the Accessibility Hierarchy [34], the Active Filler and Recent Filler Strategies proposed by the garden-path model [35], or the Dependency Locality Theory [36]. Leaving aside particular differences among these theories, most of them recognize a syntactic principle that relates the relativizer with its original position within the RC, so the longer the distance between these units is, the more costly its processing becomes as more incoming constituents need to be kept active by the parser until they are finally integrated. In English, as well as in other languages, the distance between the relativizer and its original position is longer in ORC when compared to SRC—identified in (2) by the dash, which would explain this processing asymmetry.

Nevertheless, and similarly to what has been previously observed in relation to high/low attachments, the asymmetry between SRCs and ORCs has not showed up in every language under study so far—e.g., in Basque, ORCs are easier to process than SRCs [37], and this pattern has also been observed in Chinese [38,39] and in some studies with Japanese [40], and more interestingly, it can be counterbalanced when manipulating certain extra-syntactic features. For example, Traxler et al. [30] (also [31,41,42,43,44]) found that the asymmetry between these two types of RCs can be neutralized when the antecedent NP (or head noun) is inanimate such as *movie* in (3), or similarly, when the NP within the RC is a personal pronoun such as *he/him* in (4) [45,46].

(3)

a. The movie [that pleased the director] received a prize at the film festival.
b. The movie [that the director watched] received a prize at the film festival.

(4)

a. The reporter that attacked him admitted the error.
b. The reporter that he attacked admitted the error.

As for Spanish, there is only one study to date that has addressed this question [47], and the results show, as in English, a SRC advantage when the head noun is animate—such as in (2) above, but a neutralization of this asymmetry when it is inanimate (3).

As previously reported, the problems of attachment ambiguities and subject-object asymmetry have become central when it comes to the study of RC processing. Considering the theoretical questions presented at the beginning of the introduction, the findings reported so far provide evidence that syntactic processing is not completely autonomous, but rather it seems that non-syntactic information plays a role when building sentence structure from early stages, regardless of whether this happens in a cascaded or fully interactive way, or in an incremental or discontinuous manner.

### 2.2. Function Words: Relativizers

Interestingly enough, there is a third aspect related to the structure of RCs that might be useful in order to fully understand how these sentences are processed—and therefore how different kinds of linguistic information interact when comprehending language: In languages such as Spanish, the same RC can be introduced by different relativizers, for instance, *que* (“that”; in English, *that* is considered a complementizer when introducing a RC [48], but in Spanish, *que* is always labeled as a relative pronoun when heading a RC [49,50]) in (5a), or *donde* (“where”) in (5b).

(5)

a. Fui a visitar la ciudad en la que nací.

I went to visit the city (that) I was born in.

b. Fui a visitar la ciudad donde nací.

I went to visit the city where I was born.

There are some syntactic contexts in which Spanish relativizers cannot vary [48], but when they do, as in example (5), this variation does not change the syntactic structure of the sentence nor its referential meaning. Nevertheless, Spanish relativizers have different linguistic features, so they can be manipulated in order to test whether and how these features influence sentence processing. First, Spanish relativizers may differ in semantic features [49,50]: Thus, the Spanish relativizers *que* (“that”) and *el cual* (“which”) lack semantic features, so they can combine with any head noun (human, object, place, time...); other relativizers, such as *quien* (“who”; Spanish *quien* and English *who* are not completely equivalent either, as they differ in syntactic combinations. For example, in contrast to English *who*, Spanish *quien* can only head prepositional restrictive RCs (el médico a quien llamé—the doctor whom I called)) or *donde* (“where”), possess intrinsic semantic features, which must be shared with the head noun: *quien* refers to human beings, while *donde* carries a locative meaning. Second, Spanish relativizers also differ in lexical frequency as it will be reported in the corpus study of Section 3. Based on these differences among Spanish relativizers, one of the aims of our study is to compare the processing of sentences in which a RC can be introduced by a more frequent relativizer—*que* in (6a), when compared to a less frequent one—*donde* in (6b), and also by a relativizer possessing semantic features—*donde* in (6b)—with another lacking them—*que* in (6a).

(6)

a. La policía registró el barrio del que proceden los muchachos desaparecidos.

The police searched the neighborhood which the missing boys come from.

b. La policía registró el barrio de donde proceden los muchachos desaparecidos.

The police searched the neighborhood where the missing boys come from.

Previous studies on RC processing have mainly analyzed the influence of lexicosemantic information based on the manipulation of content words, such as the antecedent NP or the NPs inside the RC; however, function words such as relativizers also differ in semantic features and lexical frequency, and so they could also be taken into account in order to examine whether and how this extra-syntactic information constrains sentence processing. However, as far as we know, there are just a few studies so far addressing these questions from the manipulation of function words.

Firstly, Tabor et al. [51] addressed the question whether the lexical frequency of an ambiguous function word such as *that* could be relevant when comprehending sentences. *That* may be interpreted as a determiner, a conjunction or a pronoun, the conjunction interpretation being the most frequent one according to Gibson [52]. Tabor et al. used a self-paced reading paradigm— which consists of presenting a written sentence in segments (words or phrases) that participants must read one at a time while they press a key in order to advance to the next segment—to compare reading times for the ambiguous word *that* versus an unambiguous word such as *those* following a verb like *visited*. This verb cannot take a clause as complement, so both *that* and *those* must be interpreted as determiners.

(7)

a. The lawyer visited that cheap hotel to stay for the night.
b. The lawyer visited those cheap hotels to stay for the night.

The authors recorded longer reading times for ambiguous sentences (7a), meaning they were more costly to process than unambiguous ones (7b). These results were interpreted as an interference of the more frequent interpretation of *that* as a conjunction, even though this interpretation was not possible in the syntactic context examined. Once readers realized this interpretation was not correct, they had to inhibit it in order to activate the correct one—*that* as a determiner—leading to longer reading times.

Gibson [52] also addressed this question, pointing out that Tabor et al. [51] did not distinguish the particular kind of frequency that influenced their results: In absolute terms, *that* is more frequently interpreted as a conjunction, but this is also the most frequent interpretation in the context Tabor et al. analyzed—that is, after a verb. Gibson wondered whether these results would be replicated when using a syntactic context in which the interpretation of *that* as a determiner is more frequent, for instance, after a preposition, as in (8).

(8)

a. The lawyer for that skilled surgeon asked for a raise.
b. The lawyer for this skilled surgeon asked for a raise.

Gibson [52], using also the self-paced reading technique, recorded longer reading times for ambiguous versus unambiguous sentences in both contexts—post-verb (7a versus 7b) and post-preposition (8a versus 8b), suggesting that the interpretation of *that* as a conjunction, although impossible in (8), was also activated. In addition, Gibson found no interaction between syntactic context and ambiguity, which means that the influence of lexical frequency occurs systematically regardless of syntactic context. Both Gibson and Tabor et al. [51] interpreted these results as supporting interactive accounts: If the parser only considered syntactic information, it would activate the syntactically correct interpretation of *that*, yielding no differences in the processing of ambiguous versus unambiguous sentences. However, the fact that the most frequent interpretation of the ambiguous function word was also activated shows that the parser considers non-syntactic information, such as lexical frequency, when building the structure of a sentence.

Other studies have employed experimental paradigms that are more sensitive to changes in processing as they occur along time, such as eye-tracking, which may enable to tell apart early and late decisions made by the parser. For instance, Schmauder et al. [53] compared sentences in which the target word—in italics—was a function (9) versus content word (10), with a higher or lower frequency rate (9a versus 9b; 10a versus 10b).

(9)

a. As we looked *across* the crowd we could see Dad’s bright red jacket.
b. As we looked *amidst* the crowd we could see Dad’s bright red jacket.

(10)

a. The old-fashioned *method* was far more effective than any modern one.
b. The old-fashioned *helmet* was far more effective than any modern one.

They measured the spillover effect, that is, the duration of the first fixation following the target word, which according to these authors, reflects the integration cost of the target word within the syntactic structure. The results showed longer first fixations following less versus more frequent function words (9b versus 9a); however, there were no differences between content words due to lexical frequency (10a versus 10b). Schmauder et al. [53] argued in favor of an early influence of lexical frequency on the syntactic integration of a function word: The less frequent a function word is, the more costly its syntactic integration becomes.

Nevertheless, Schmauder et al.’s study [53] has some limitations that should be taken into account. First, these authors analyzed the spillover effect for the first fixation, that is, the duration of a fixation when the eyes first meet a word. This measure has been traditionally related to lexical retrieval [54,55,56], so it could be questioned whether this effect reflects a syntactic integration cost due to the low frequency of a function word or, simply, a cost in the retrieval of a less frequent function word. Regarding this question, Rayner et al. [54] argued that first-pass duration (so named when the target region includes more than one word [57], and gaze duration when it contains only one word [58]), instead of first fixation, is a better measure to analyze syntactic integration as it shows that, after a first fixation, the reader re-fixates a word before leaving it in a regressive or progressive manner. This refixation would indicate a difficulty when establishing a relationship between the fixated word and previous ones, that is, an integration cost of the target word into the syntactic structure [56].

Similarly, Rayner et al. [54] have pointed out that a spillover effect could be related to a syntactic integration cost but also to a lexical retrieval cost: If activating a lexical unit is harder than expected, as it normally occurs with low frequency words, this cost can be dragged onto the next word, resulting in a spillover effect. In order to distinguish one cause from the other, Rayner et al. recommended to run a correlation analysis between reading times of the target word and the following one: A positive correlation between these two words would mean that the spillover effect is a consequence of a lexical retrieval cost; on the contrary, the lack of a positive correlation would denote that the spillover effect reflects a cost that goes beyond retrieving the target word from the mental lexicon and is related to syntactic integration. Unfortunately, Schmauder et al. [53] did not perform such an analysis, so the spillover effect they found could be related to either a syntactic integration cost—as they argued—or a lexical retrieval cost.

In sum, the results of the studies reviewed so far are not conclusive about the influence of function words and their lexical information on sentence processing. On the one hand, Tabor et al. [51] and Gibson [52] provided evidence for this influence but could not demonstrate that it occurs at early stages as self-paced reading techniques are not sensitive to early processing. In this sense, both modular and interactive theoretical accounts could explain their results. On the other hand, Schmauder et al.’s results [53] could be related either to a syntactic integration cost, thus reflecting some influence of lexical frequency on syntactic processing at early stages, or merely to a lexical retrieval cost. Therefore, these studies do not provide a satisfactory answer to the question at hand, that is, does lexical information related to function words constrain or guide sentence building operations? Given this state of affairs, we argue that Spanish relativizers could be useful in order to examine this question, as they differ in lexical frequency (as it will be reported in Section 3), and also in semantic information, with some of them possessing intrinsic semantic features and others lacking them. In fact, the semantic differences between relativizers allow us to analyze the influence of these features on sentence processing based on the manipulation of function words, as we have no evidence of such a study so far. E. Fernández [59] suggested a similar study to the one presented here but with a different purpose: She asked whether the preference for high or low attachment of a RC with two attachment sites within a noun phrase could be determined by the relativizer introducing it; however, such an experiment has not been conducted so far.

### 2.3. RC Type

Beyond relativizers, there is a second characteristic related to RCs that has scarcely been studied in relation to sentence processing: RC type. In languages such as Spanish, RCs can be classified into two different types: Restrictive (7) and Non-Restrictive (11). These two types of RCs differ, on the one hand, in their semantic function: Restrictive RCs are used to identify the referent of the head noun, which is unknown prior to introducing the embedded clause, whereas non-restrictive RCs add an extra meaning, unnecessary to identify the referent of the head noun, as this is already known by the reader (see [60,61] for different accounts of non-restrictive RCs in English, and [62] for a comprehensive review of Spanish RCs). In addition, Spanish restrictive and non-restrictive RCs also differ in frequency (as it will be reviewed in Section 3), so they can also be compared so as to examine whether the frequency of these syntactic structures influences sentence processing.

(11)

a. La policía registró mi barrio, del que proceden los muchachos desaparecidos.

The police searched my neighborhood, which the missing boys come from.

b. La policía registró mi barrio, de donde proceden los muchachos desaparecidos.

The police searched my neighborhood, where the missing boys come from.

Most studies on RC processing have been carried out with restrictive RCs. The comparison between restrictive and non-restrictive RCs has been addressed mainly with the interest of exploring the contribution of prosody and, to a lesser extent, punctuation marks (i.e., commas) in spoken and written RCs, respectively. As regards spoken RCs in Spanish, restrictive and non-restrictive types are distinguished on the basis of the location of pauses and changes in pitch at constituent boundaries. Thus, restrictive RCs usually bear a longer pause and a rising pitch at the end of the main clause (or the antecedent NP), plus a reset to lower pitch at the RC onset, whereas non-restrictive RCs bear a shorter pause (usually not perceptible) and a falling pitch at the clause boundary (or the antecedent NP), with reset to higher pitch at RC onset [63]. Written RCs, in turn, mirror this intonational pattern by regularly placing a comma after the head noun to identify non-restrictive RCs, while restrictive RCs conventionally follow the head noun without punctuation (see examples 7 and 11 above). Spanish is no different from other European languages in this regard. In any case, we should bear in mind that the presence of a comma right after the head noun in the written version of non-restrictive RCs (or its absence in restrictive RCs), and before the relativizer, may reveal the type of RC the reader is facing, and this has implications for the interpretation of eye-tracking data, as we shall see later.

To the best of our knowledge, only a handful of studies have addressed the contrast between restrictive and non-restrictive RCs in written modality in terms of processing difficulty. In a study by Grodner et al. [64], restrictive and non-restrictive RCs were compared in a self-paced reading task under two contextual conditions: A null context, and a supportive context preceding the target RC for each kind of RC. For restrictive RCs—e.g., “The postman that the dog bit…”, the supportive context introduced two referents compatible with the denotation of the subject noun of the target sentence, whereas for non-restrictive RCs—e.g., “The postman, who the dog bit…”, it only gave one such referent. The results showed a reversed pattern of reading times in the region following the relativizer, i.e., “the dog bit”, across RC types as a function of context: reading times were longer for restrictive RCs under the null context condition and for non-restrictive RCs under the supportive context. Therefore, when read in isolation, restrictive RCs are harder to process than non-restrictive ones, while the opposite is the case with a facilitating context. The authors take this pattern of results as evidence for the early influence of discourse context on the interpretation of unambiguous sentences.

In addition, a few studies have addressed the effects of punctuation marks on written sentence processing. In this regard, it has been shown that the presence of a comma located at phrasal or clausal boundaries of non-restrictive RCs and other structures, such as vocatives and parentheticals, tends to increase processing times in eye-tracking measures—such as fixation durations or number of fixations—on the section of the sentence preceding the comma while facilitating the processing of the following section, as shown by larger saccades into that region ([65], but see the contradictory results for non-restrictive RCs in Hirotani et al. [66]).

### 2.4. Present Study

Based on these two manipulations—relativizer and RC type—we set out to analyze whether and how frequency and semantic information can determine and guide RC processing in Spanish. To examine this question, we conducted an eye-tracking experiment in which participants read Spanish RCs such as (7) and (11), differing in the heading relativizer (*que* versus other) and the RC type (restrictive versus non-restrictive). Previous studies on RC processing had a different purpose (as reported in Section 2.1), which makes it difficult to anticipate the readers’ behavior, and underscores the exploratory nature of the current study. However, there are still some predictions that can be made taking into account different theoretical accounts. 

First of all, a modular account such as the garden-path model claims that sentence processing is only determined by syntactic principles at early stages (with non-syntactic information playing a role only at late stages, when the sentence structure has already been computed), and particularly in RC processing, by two syntactic strategies: Active Filler and Recent Filler [35]. Once the parser identifies the position of a relativizer, the Active Filler Strategy is applied, so the filler formed by the relativizer needs to be kept active until a gap is found. This would fire a second strategy, Recent Filler, such that the first gap encountered would be filled by the most recent active filler. Concerning our study, this model would predict no differences in the processing of sentences (12a) and (12b), at least at early eye-tracking measures, as a consequence of the kind of relativizer heading the RC, since in both cases the parser would identify a filler (the relativizer) right after the direct object (*el barrio*), and this structure is taken to be common to all conditions regardless of whether the relativizer is *que* or other (*el cual, quien,* or *donde*). The filler would be kept active by the Active Filler Strategy until a gap was found, and the Recent Filler Strategy would try to detect a gap for this filler, which again appears at the same position in all conditions, that is, after the embedded verb. Only at a later stage and, hence, showing up in late eye-tracking measures, could non-syntactic information influence RC processing, so that differences between (12a) and (12b) due to lexical frequency and/or semantic features of the relativizer would only show up at this point.

(12)

- La policía registró el barrio [del que_i_ proceden *h*_i_ los muchachos desaparecidos].
  The police searched the neighborhood [which the missing boys come from h].
- La policía registró el barrio [de donde_i_ proceden *h*_i_ los muchachos desaparecidos].
  The police searched the neighborhood [where the missing boys come from h].

As for the contrast between restrictive and non-restrictive RCs, the predictions of the garden-path model would depend on the relative complexity of either type of RCs: If both types shared the same syntactic configuration, there should be no processing differences, but if restrictive and non-restrictive RCs are shown to attach at different levels—i.e., as a sister of D within the DP in restrictive RCs (see example 13a) and as a sister of DP at a higher structural level in non-restrictive RCs (13b), the parser should have more processing difficulties with the latter.

(13)

a. Restrictive RC: [_DP_ the [_NP_ poets [_CP_ who spoke French]]].
b. Non-restrictive RC: [_DP_ [_DP_ the [_NP_ poets]] [_CP_ who spoke French]].

Other modular accounts, such as the Tuning hypothesis [9], would make moderately different predictions from those of the garden-path model. The Tuning hypothesis would accept that the frequency of a syntactic structure can influence sentence processing at early stages, so differences in the processing of restrictive versus non-restrictive RCs could be found. Particularly, those structures that are more frequent—non-restrictive RCs in most of the cases, as we shall see in the next section—would be easier to process. As for relativizers, this theory predicts no differences in the processing of RCs due to lexical or semantic features, which means that the processing of (12a) and (12b)—or, similarly, (11a) versus (11b)—would not differ, again at least at early stages. In this sense, the Tuning hypothesis can also be considered a modular account, as it preserves the autonomy of syntactic information at early stages, devoid of any semantic features.

On the other hand, interactive accounts claim that non-syntactic information can guide the process of building the syntactic structure of a sentence from the very beginning, and not only at late stages. In particular, usage-based accounts [67,68,69,70,71] argue that sentence processing is determined by distributional patterns learned over time, meaning that those patterns are easier to recognize and hence to process. One of the factors that make a pattern easier to recognize is frequency not only at a syntactic level but at any linguistic level. Consequently, usage-based accounts predict that those RCs which are more frequent and which are introduced by a more frequent relativizers would be easier to recognize and hence to process, showing shorter reading times not only at late stages but from early eye movement measures.

Similarly, usage-based accounts consider that distributional patterns may also capture semantic information, so these features might influence RC processing as well. In this case, two hypotheses could be put forward. On the one hand, it might occur that the activation of the relativizer’s semantic features—if it has any—would take some extra time, so its identification and integration would be more costly as compared to a relativizer lacking semantic features. This would result in longer reading times for those RCs headed by relativizers that possess intrinsic semantic features—*donde* (12b)— versus relativizers lacking semantic information—*que* (12a). On the other hand, it could also be predicted that sharing semantic features between the head noun and the relativizer would facilitate the activation of the latter and its integration into the syntactic structure, leading to shorter reading times in comparison to a relativizer lacking semantic features (12b versus 12a). As for the role of the semantic features of restrictive and non-restrictive RCs, interactive accounts argue that non-syntactic information can influence and constrain sentence building operations from early stages. Bearing in mind that restrictive and non-restrictive RCs perform different semantic functions in fixating the reference of their head noun, it seems plausible to consider that interactive accounts would predict processing differences between restrictive and non-restrictive RCs. However, these models do not specify how semantics constrains RC processing in particular, which makes it difficult to provide detailed predictions. Therefore, our study will hopefully help clarify whether and how the semantic function of each RC type results in processing differences.

Thus, our study purports to find out whether or not lexico-semantic information related to function words such as relativizers, as well as to RC type, can influence RC processing in Spanish. We first provide the results of a corpus study (Section 3), aimed at determining the frequency of both relativizers and RC types in Spanish. Then, we will report the results of an eye-tracking experiment (Section 4), designed to examine RC processing when manipulating relativizers and RC type. We will end up with a discussion of the results (Section 5) and some concluding remarks (Section 6).

## 3. Corpus Study

To control for lexical and structural frequencies, we carried out a corpus study. Previous studies have analyzed the frequency of Spanish relativizers [72,73,74,75]; however, most of them suffer from limitations, such as not discriminating the syntactic contexts in which relativizers vary, not using systematic criteria, or more frequently, not performing quantitative analyses when comparing relativizers’ frequencies [76]. For these reasons, we ran a corpus study in which we analyzed a total number of 134,018 words spanning a period of time from 2000 to 2013. These words were taken from different European Spanish corpora (Table 1) with the aim of analyzing both formal and informal registers, as well as oral and written language. This makes another difference between our study and previous ones, which have generally analyzed a particular register of a particular linguistic variety. Table 1 shows the number of words analyzed for each linguistic variety, how many of those words were relativizers, how many of those relativizers appeared in contexts where variation is allowed in Spanish, and finally, the corpus from which data was taken. Statistical analyses showed no differences in the total number of relativizers, χ^2^(3) = 1.21, *p* > 0.1, or in the total number of relativizers in variation contexts, χ^2^(3) = 2.72, *p* > 0.1, across linguistic varieties.

Table 1 displays absolute and relative frequency for all the Spanish relativizers (*que, el cual, quien, donde, cuando, como, cuanto,* and *cuyo*); however, our study is focused on the processing of RCs introduced by four of these units: *que* (“that”), *el cual* (“which”), *quien* (“who”), and *donde* (“where”). The reason why we decided to analyze only four relativizers was to include a larger number of experimental materials in the eye-tracking experiment in order to gain statistical power. Particularly, we chose the previous four relativizers for two main reasons: (1) as it will be shown, they differ in frequency rates, as well as in semantic features, which would enable us to examine the possible influence of these features on RC processing, and (2) in contrast to other relativizers, these four units make it possible to create experimental materials with the same structure across conditions and which would also sound natural.

Table 2 shows the absolute and relative frequency of the Spanish relativizers *que* (“that”)*, el cual* (“which”)*, quien* (“who”), and *donde* (“where”) in the contexts where they can vary for both types of RCs—restrictive and non-restrictive. As mentioned in Section 2.2, variations are not freely allowed in Spanish between these four relativizers: *Que* can replace the other three relativizers in any context, but these three relativizers cannot always replace *que*, and in addition, they cannot generally substitute each other, e.g., *quien* cannot replace *donde* or vice versa [49,50]. For this reason, we will report the results based on three relativizer contrasts: *que* versus *el cual*, *que* versus *quien*, *que* versus *donde*. In this regard, it is important to note that, in some contexts, *que* may be replaced by both *el cual* or *quien* and, in others, by both *el cual* or *donde*. These data have been added to the frequency of *que* in contrasts of *que* versus *el cual*, as well as in *que* versus *quien* (or *que* versus *donde* when the antecedent was a locative.

Taking into account count data from Table 2, we performed a Poisson regression using the glm function in R [77]. Models were built with RC type, relativizer, and their two-way interaction as fixed effects and linguistic variety as a random effect. As dependent variables, we analyzed relativizers’ absolute frequency. Models were compared conducting likelihood-ratio tests with ANOVA in R (see Tables in Section A of the Supplementary Materials for a full description of the models’ reports), and *p*-values were adjusted using the Holm–Bonferroni method [78].

### 3.1. Que versus el Cual

**Figure 1:**
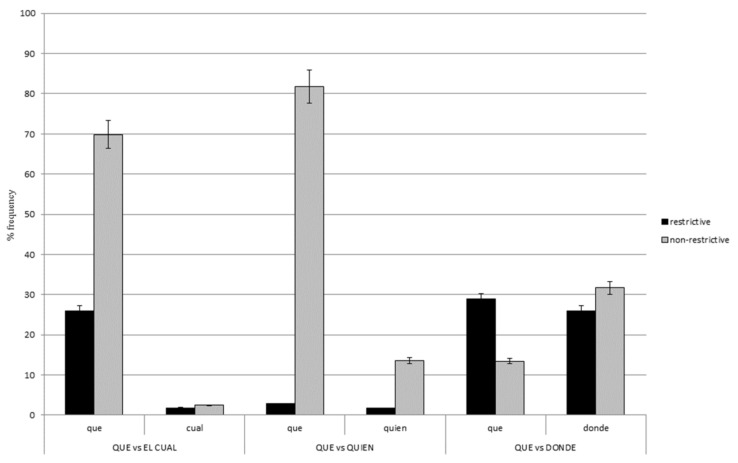
Pairwise comparisons of the relative frequency of the Spanish relativizers *que, el cual, quien,* and *donde* in restrictive and non-restrictive RCs in Spanish corpora.

Figure 1 shows the frequency of *que* and *el cual* in both restrictive and non-restrictive RCs. For this contrast, there was a RC type x relativizer interaction, *z* = 2.89, *p* < 0.01, for although *que* is much more frequent than *el cual* in both types of RCs, this difference turned out to be larger for non-restrictive than restrictive RCs (69.86% versus 25.89%). Similarly, non-restrictive RCs are more frequent than restrictive RCs regardless of the heading relativizer; however, this difference seems to reach significance only for sentences with *que* versus *el cual* (43.97% versus 0.68%).

### 3.2. Que versus Quien

Figure 1 also displays the frequency of *que* and *quien* in both restrictive and non-restrictive RCs. For this contrast, there was a RC type x relativizer interaction, *z* = −2.79, *p* < 0.05, which shows *que* is more frequent than *quien* in both restrictive and non-restrictive RCs, but this difference proved to be much larger for non-restrictive than restrictive RCs (68.27% versus 1.09%). Additionally, and similarly to the previous contrast, non-restrictive RCs are more frequent than restrictive RCs with both relativizers, but this difference was larger for sentences with *que* versus *quien* (79% versus 11.82%).

### 3.3. Que versus Donde

Finally, Figure 1 also shows the frequency of *que* and *donde* in both types of RCs. For this contrast, there was a RC type x relativizer interaction, *z* = −4.87, *p* < 0.001, since *que* is more frequent than *donde* in restrictive RCs, whereas *donde* is more frequent than *que* in non-restrictive RCs. Additionally, restrictive RCs are more frequent than non-restrictive RCs in sentences headed by *que,* whereas the opposite pattern was found for sentences with *donde*: Non-restrictive RCs are more frequent than restrictive ones.

To sum up, the contrasts *que* versus *el cual* and *que* versus *quien* exhibit a similar pattern, since *que* turns out to be more frequent than either *el cual* or *quien* in Spanish, especially in non-restrictive RCs. In addition, non-restrictive RCs are more frequent than restrictive RCs regardless of the heading relativizer but especially when headed by *que*. On the other hand, the contrast *que* versus *donde* shows a different pattern, as now the frequency rate of these two relativizers is somehow determined by RC type: Non-restrictive RCs are more frequently headed by *donde* versus *que,* while restrictive RCs appear more frequently introduced by *que* versus *donde*.

Based on the results of our corpus study, Table 3 summarizes the main differences between Spanish relativizers in terms of lexical frequency and semantic features. These features were then included in our eye-tracking study in order to determine whether or not they influence sentence processing.

## 4. Eye-Tracking Study

### 4.1. Method

#### 4.1.1. Participants

Forty-eight undergraduate students from the Autonomous University of Madrid participated in this experiment in exchange for course credits. All were Spanish native speakers and had normal or corrected to normal vision (mean age = 19.41, *SD* = 0.84; 7 males).

#### 4.1.2. Design

We conducted an eye-tracking experiment with a two-way repeated measures design. Independent variables, both within-subjects, were (a) type of RC (restrictive versus non-restrictive) and (b) relativizer (*que* versus other), resulting in four conditions, as exemplified in Table 4: restrictive *que*, restrictive other, non-restrictive *que*, and non-restrictive other. This experimental design was applied in three contrasts, *que* versus *el cual*, *que* versus *quien*, and *que* versus *donde*, so the other conditions correspond to *el cual*, *quien,* and *donde*, respectively. As dependent variables, six eye movement measures were analyzed [56]: First fixation (the duration of the first fixation on a target region); first-fixation rate (probability of fixating a region during first pass); first-pass duration (the total duration of all fixations on a target region before exiting it for the first time, either in a regressive or progressive manner); first-pass regression (probability of making a regression during first pass); quasi-first-pass reading time (the total duration of all fixations on a target region before exiting it for the first time in a progressive manner) and second-pass duration (the total duration of all refixations on a target region). The first three measures are generally referred to as “early” measures, as they reflect the first contact of the eyes with the text (and in consequence, a first stage of processing), while the last measure (second-pass duration) is considered a “late” measure, as it includes any possible reanalysis of the text. As for first-pass regression and quasi-first-pass reading time, there is some controversy regarding their status: They record the first contact of the eyes with the text but purportedly reflect some difficulty when integrating a word and the resulting reanalysis performed to overcome this difficulty [56]. For this reason, we will refer to these two measures as “intermediate”. Finally, we also took into account parafoveal processing—that is, the processing of the following word (*n* + 1) when the eyes are fixating on the word *n*.

#### 4.1.3. Materials

We created 168 experimental items—56 experimental items per contrast—and 180 filler items. Four lists of experimental items were constructed, each containing one version of each item. Participants were assigned randomly to one of the four lists, so all of them read 42 experimental items under each condition, i.e., 14 for each relativizer contrast, and only one version of each item. Similarly, each version of the items was read by twelve participants, resulting in a within- and between-subject design.

Regarding syntactic structure, experimental items included subject (region 1) + main verb (region 2) + direct object, functioning as antecedent or head noun (region 3) + RC, which was formed by a relativizer (region 4) + a verb (region 5) + a complement (region 6). Table 4 exemplifies how items were divided into six regions according to their syntactic structure in order to perform statistical analyses (see Section B of the Supplementary material for a full description of the experimental stimuli).

Conditions differed in (a) RC type as revealed in the head noun (R3), and (b) the relativizer (R4). The head noun was the same singular noun among conditions; however, in restrictive RCs it was preceded by an article—*el/la* (“the”), which implies that the referent is unknown and will be identified by the RC, whereas, in non-restrictive RCs, it was preceded by a possessive determiner—*mi, su* (“my”, “his/her”), meaning that the referent of the antecedent is already known, and by a comma following the noun, as required by Spanish orthographic rules. Relativizers also differed among conditions (*que* versus *el cual, quien,* and *donde*). In order to avoid large differences in the number of characters in the relativizer region (R4), all relativizers performed a prepositional syntactic function, so they were always preceded by a preposition. This aspect has important consequences for que-conditions, because the presence of a preposition requires also the use of an article before this relativizer. This phenomenon reduces to just one character the difference between *que* versus *el cual*-conditions (9.5 characters versus 10.5), as well as between *que* versus *quien*-conditions (6.03 versus 7.03). As for the contrast *que* versus *donde*, there was a mean difference of two characters between conditions (7.84 characters for que-conditions versus 5.89 for donde). In order to control for differences in the length of a region, some authors recommend the use of reading time per character, that is, the division of the total time it takes to read a region (known as raw data) by the number of characters of that region including spaces [15,79,80,81,82]. Nevertheless, we preferred to use raw data for two reasons. First, conditions differ in one or two characters, which is a negligible difference, especially when comparing short frequent words such as function words [8]. Second, reading time per character assumes a linear relation between fixations and the number of characters, which may not be generally true [8,55].

Filler items had the same length as experimental ones, but did not include RCs. They had the following structure: subject + main verb + direct object + subordinate clause, which was formed by an initial conjunction + a verb + a complement. Both types of items (experimental and filler) were randomized and followed by a comprehension question, which could refer to the content of either the main or the embedded clause.

#### 4.1.4. Procedure

Eyelink 1000 Plus (SR Research, Ottawa, ON, Canada) was used in order to monitor participants’ eye movements while reading both experimental and filler items. The eye-tracker had a 2000 Hz. sample rating, with an angular resolution of 1.46 degrees. Materials were displayed in a MultiSync EA221WM screen, connected to the eye-tracker. Sentences and questions appeared in a single line, in the middle of the screen, on a grey background. Letters were in black, Courier New font, and size 18.

The experiment was conducted individually in two sessions with at least one week interval between sessions in order to avoid priming effects—items were different in each session, but they had the same syntactic structure. Participants sat down in front of a computer screen at an approximate distance of 60 cm. Instructions were explained before starting the task: Participants had to read the sentences on the screen in silence at a normal pace. After each sentence, a comprehension question appeared and participants had to respond “yes” or “no” by pressing a key—“s” or “n”, respectively. They also had to press a key after each sentence in order to visualize the corresponding question. While reading the material, participants placed their heads in a chin rest so as to minimize head movements. Vision was binocular during the whole experiment, but only movements from the right eye were recorded.

Before starting the experiment, the eye-tracker was calibrated in order to obtain the best possible recording of the eye movements. Calibration was repeated in the middle of each session after a short break, and also when the tracker was out of alignment. Similarly, before proceeding to the next trial a fixation point (i.e., drift correct) was displayed on the left side of the screen in order to calibrate the apparatus and to locate participants’ gaze, thus avoiding a previsualization of the sentence. 

### 4.2. Results

#### 4.2.1. Comprehension Task

Participants responded correctly to 95% of the comprehension questions. We performed mixed-effect logistic regression using the glmer function in R [83,84]. Mixed logit models were built with response accuracy as the dependent variable, and RC type, relativizer and their two-way interaction as fixed effects. Subjects and items were added as random effects. In order to control for individual variation in the response-reaction time (RT) relation, a random effect slope for RT was added to subjects, items or both. Models were compared conducting a likelihood-ratio test with the ANOVA function in R (see Tables in Section C of the Supplementary Materials for a full description of the models’ reports), and *p*-values were adjusted using the Holm–Bonferroni method [78]. 

Results showed no significant differences (*p* > 0.05) in the contrasts *que* versus *el cual* and *que* versus *quien*, meaning that participants regularly understood experimental sentences irrespective of the RC type or the relativizer heading it (Table 5). In the contrast *que* versus *donde* there was a significant interaction between the two independent variables, *z* = -2.54, *p* = 0.03, since restrictive RCs presented a higher response accuracy with *que* versus *donde* (96% versus 94%); in contrast, non-restrictive RCs showed a better performance with *donde* versus *que* (96% versus 94%). This latter result suggests participants understood better restrictive RCs with *que* and non-restrictive RCs with *donde*, thus mirroring the frequency rates of the corpus study for this contrast (see Section 2); however, the remaining two conditions (non-restrictive RCs with *que* and restrictive RCs with *donde*) also present a high response accuracy (more than 90%), confirming participants understood most of the experimental sentences in these two conditions as well.

#### 4.2.2. Eye Movement Measures

Before analyzing the eye-tracking record, fixations longer than 2000 ms were removed. Similarly, fixations shorter than 80 ms and located at least 1.46 degrees apart from another fixation were also removed; however, when located at a distance shorter than 1.46 degrees from another fixation, they merged into one. This procedure resulted in 0.16% of the data being excluded.

Eye-tracking data was analyzed using the lme4 package in R [85]. Linear mixed-effect models were built with RC type, relativizer, and their two-way interaction as fixed effects. Additionally, we included random slopes for RC type and relativizer by subject and by item. As dependent variables, we analyzed four of the eye movement measures described above (first fixation, first-pass duration, quasi-first-pass reading time, and second-pass duration) for the three regions of interest (R3, R4, and R5, corresponding to the head noun, the relativizer, and the RC verb, respectively). The remaining two eye movement measures (first-fixation rate and first-pass regression) were analyzed using mixed-effect logistic regression in R, where the presence of a fixation was coded as 1 and its absence as 0. For the three regions of interest mentioned above, mixed logit models were built with RC type, relativizer, and their two-way interaction as fixed effects and subject and item random effect slopes for RC type and relativizer. Both linear mixed-effect models and mixed logit models were compared conducting likelihood-ratio tests with ANOVA in R, and *p*-values were adjusted using the Holm–Bonferroni method.

For ease of presentation, we will report the results based on the three relativizer contrasts explored in our experiment, starting with *que* versus *el cual*, then *que* versus *quien*, and finally, *que* versus *donde*. Additionally, effects will be reported by regions (R3, R4, and R5), as well as by “the way eyes move”, that is, early, intermediate, and late measures. On the other hand, and also for the sake of clarity, the subsequent discussion of the results will be framed according to the two independent variables used in our study, namely, relativizer and RC type. This will allow a clearer assessment and comparison of the role of different relativizers and RC types in the processing of these sentences, the main goal of our current research.

#### 4.2.3. Eye-Tracking Results for Que versus el Cual

**Figure 2:**
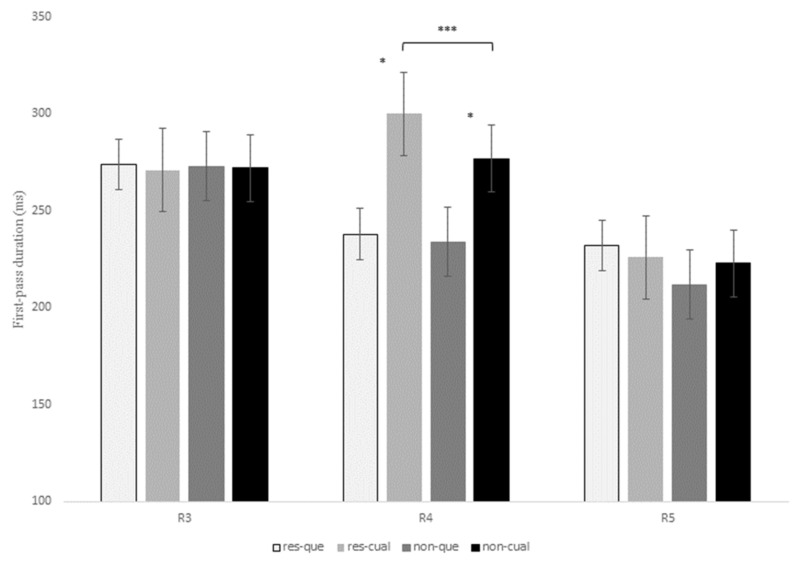
Reading time in milliseconds for first-pass duration at regions 3, 4, and 5 for the contrast *que* versus *el cual* in restrictive and non-restrictive RCs. Asterisks indicate that an effect is significant at the following alpha levels: * = *p* < 0.05; *** = *p* < 0.001.

**Figure 3:**
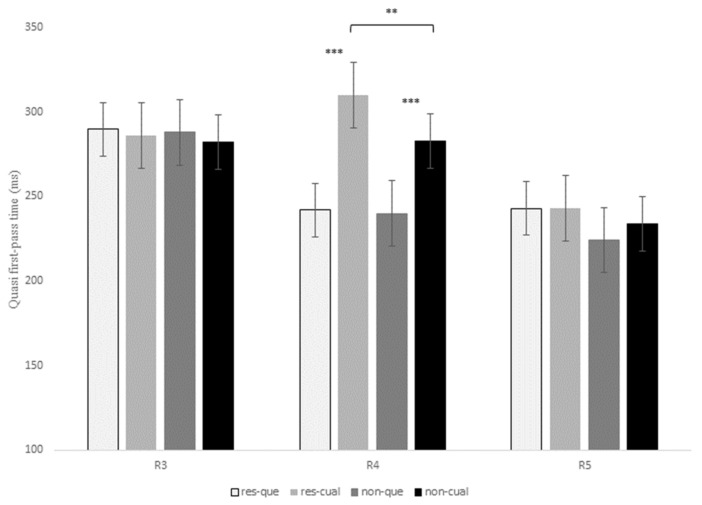
Reading time in milliseconds for quasi-first-pass reading times at regions 3, 4, and 5 for the contrast *que* versus *el cual*, in restrictive and non-restrictive RCs. Asterisks indicate that an effect is significant at the following alpha levels: ** = *p* < 0.01; *** = *p* < 0.001.

**Figure 4:**
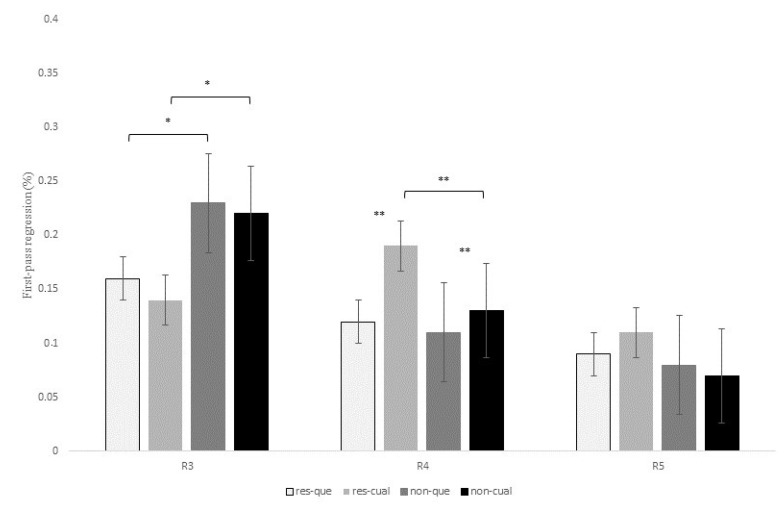
Probability of regression during the first-pass at regions 3, 4, and 5 for the contrast *que* versus *el cual*, in restrictive and non-restrictive RCs. Asterisks indicate that an effect is significant at the following alpha levels: * = *p* < 0.05; ** = *p* < 0.01.

Table 6 shows the statistical effects found in the six eye-tracking measures recorded for the contrast *que* versus *el cual* at R3, R4, and R5. In addition, Figure 2, Figure 3 and Figure 4 display mean reading times of one early reading measure (first-pass duration; Figure 2) and two intermediate reading measures (quasi-first-pass time and first-pass regression; Figure 3 and Figure 4, respectively) across regions 3, 4, and 5 (see also Tables in Section D of the Supplementary Materials for a full description of the models’ reports, as well as for mean reading times of all the eye-tracking measures).

At R3 (that is, the head noun of the RC), there was a main effect of RC type for the intermediate measure first-pass regression, *z* = 2.88, *p* = 0.01, meaning that the probability of regressing from this region to previous words during the first pass was higher for non-restrictive versus restrictive RCs (22.5% versus 15.1%). No other effects were found at this region.

At the relativizer region, R4, there was an interaction of RC type x relativizer for the early measure first-fixation rate, *z* = 3.72, *p* < 0.001, since the probability of fixating this region during the first pass was higher for restrictive RCs headed by *el cual* versus *que*, while there were no differences between non-restrictive RCs (see Table 6 below).

At R4, there was also a marginal interaction of RC type x relativizer for the early measure first-pass duration, *t* = 1.85, *p* = 0.06, for although restrictive RCs received longer fixations than non-restrictive RCs when introduced by both *que* and *el cual*, this difference turned out to be larger for the latter relativizer (22.9 ms versus 3.5 ms). This same interaction was marginal for first-pass regression, *z* = 1.86, *p* = 0.06, and reached significance for quasi-first-pass time, *t* = 2.31, *p* = 0.02, both being intermediate measures (Figure 3 and Figure 4). Finally, this interaction became also marginal for the late measure second-pass duration, *t* = 1.87, *p* = 0.06, since restrictive RCs received longer refixations during second pass than non-restrictive RCs, especially when headed by *el cual*.

On the other hand, there was a main effect of RC type at R4 for the early measure first-pass duration, *t* = −2.62, *p* = 0.01, with longer reading times for restrictive RCs when compared to non-restrictive RCs (Figure 2). This effect was also significant for quasi-first-pass time, *t* = −2.96, *p* = 0.006 (Figure 3), and second-pass duration, *t* = −2.6, *p* = 0.01, which exhibited the same pattern.

There was also a main effect of relativizer at R4 for the first-fixation rate, *z* = −3.94, *p* < 0.001, which means that there was a higher probability of fixating this region when a RC was introduced by *el cual* when compared to *que*. This effect also showed up in reading times for early measures such as first fixation, *t* = −6.08, *p* < 0.001, and first-pass duration, *t* = −6.68, *p* < 0.001, with longer reading times for *el cual* when compared to *que* in both restrictive and non-restrictive RCs (Figure 2).

In addition, there was a main effect of relativizer for first-pass regression at R4, *z* = −3.26, *p* = 0.003, meaning that the probability of regressing form this region to previous words was higher when a RC was introduced by *el cual* versus *que* (Figure 4). This effect for first-pass regression was completed by the above-mentioned interaction of RC type x relativizer for quasi-first-pass time, for despite the fact that *el cual* received longer reading times than *que* for both types of RCs, this difference turned out to be larger for restrictive than non-restrictive RCs (68.07 ms versus 42.78 ms).

Additionally, there was a main effect of relativizer for second-pass duration at R4, *t* = -3.22, *p* = 0.003, with longer reading times for *el cual* versus *que*, especially in restrictive RCs. Altogether, the effect of relativizer at R4 seems to indicate that RCs introduced by *el cual* are more difficult to process than those introduced by the more frequent relativizer *que* in both types of RCs but, especially, in restrictive RCs.

Finally, at R5, which displayed the same RC verb across all conditions, there was a marginal effect of RC type for the intermediate measure first-pass regression, *z* = −2.28, *p* = 0.06, and the late measure second-pass duration, *t* = −2.34, *p* = 0.05, meaning that there was a higher probability of regression backwards, as well as longer reading times for the RC verb of restrictive versus non-restrictive RCs. Interestingly enough, this effect exhibited the same pattern for both R4 and R5, thus suggesting that restrictive RCs seem to impose a greater processing burden than non-restrictive RCs, but was opposite to the pattern previously observed at R3. In the Discussion section, we will try to explain this variation between regions.

To conclude, R3 showed a higher probability of making a regression from this region to previous words during the first pass for non-restrictive versus restrictive RCs. On the contrary, R4 and R5 exhibited longer reading times for restrictive versus non-restrictive RCs and for sentences introduced by *el cual* versus *que* in early measures such as first fixation or first-pass duration. Similarly, once readers reached R4, they tended to make more regressions and longer refixations on this same region before advancing to the following one when a RC was headed by *el cual*, especially for restrictive RCs. This latter pattern was also reflected in late measures such as the second-pass duration, which showed longer reading times for restrictive RCs with *el cual*.

#### 4.2.4. Eye-Tracking Results for Que versus Quien

**Figure 5:**
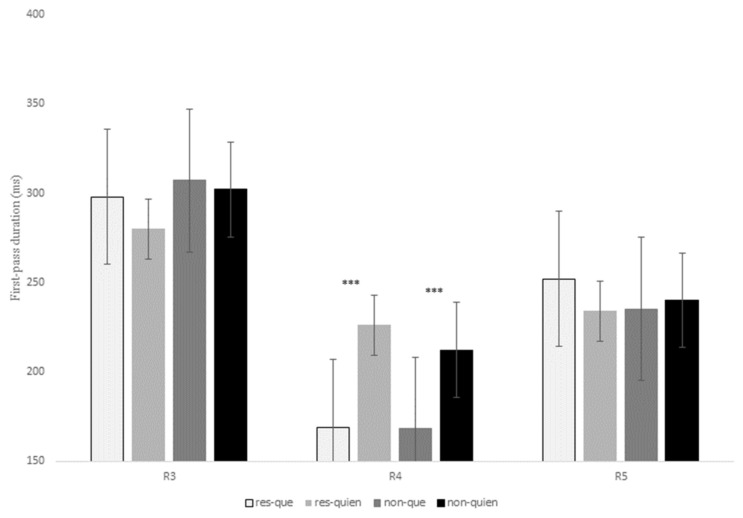
Reading time in milliseconds for first-fixation duration at regions 3, 4, and 5 for the contrast *que* versus *quien* in restrictive and non-restrictive RCs. Asterisks indicate that an effect is significant at *p* < 0.001.

**Figure 6:**
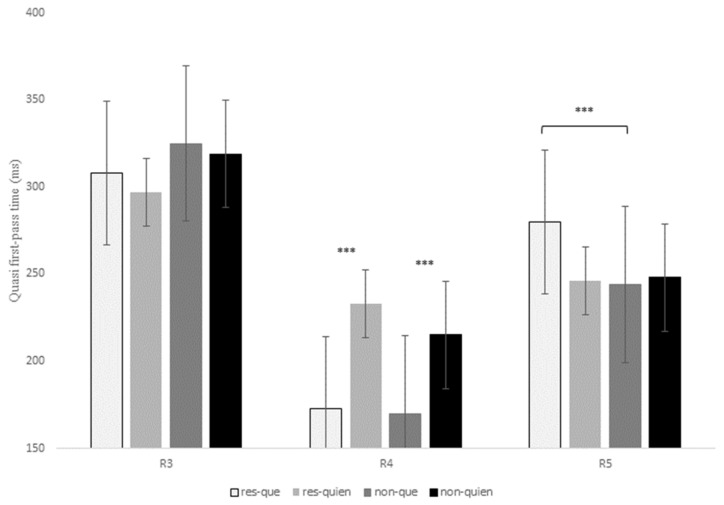
Reading time in milliseconds for quasi-first-pass reading times at regions 3, 4, and 5 for the contrast *que* versus *quien* in restrictive and non-restrictive RCs. Asterisks indicate that an effect is significant at *p* < 0.001.

**Figure 7:**
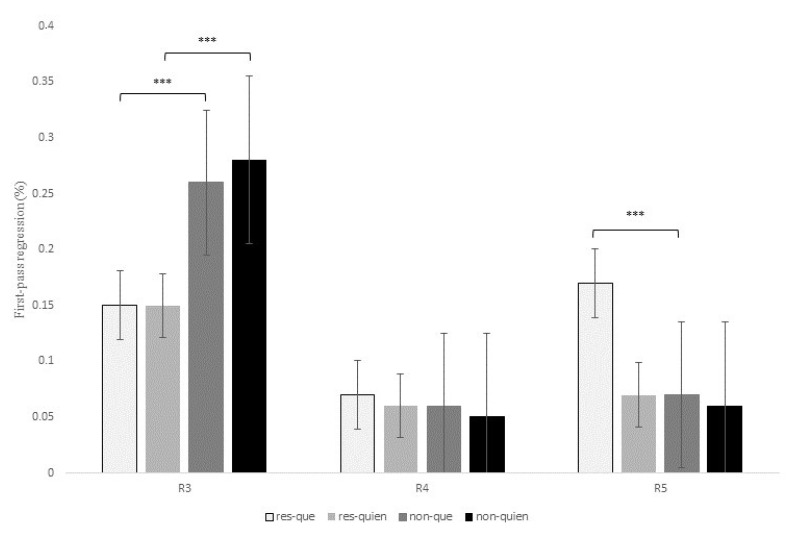
Probability of regression during the first-pass at regions 3, 4, and 5 for the contrast *que* versus *quien* in restrictive and non-restrictive RCs. Asterisks indicate that an effect is significant at *p* < 0.001.

Table 7 displays the statistical effects of the eye-tracking measures for the contrast *que* versus *quien*. Similarly, the data of first-pass duration, quasi-first-pass reading time, and first-pass regression recorded at R3, R4, and R5 are shown in Figure 5, Figure 6 and Figure 7, respectively (see also the tables in Section E of the Supplementary Materials for models’ reports and for mean reading times of all the eye-tracking measures).

At R3, there was a main effect of RC type for the intermediate measure first-pass regression, *z* = 3.64, *p* < 0.001, since non-restrictive RCs exhibited a higher probability of making a regression from the head noun to previous regions in comparison to restrictive RCs (27% versus 15%). This effect was also significant for the late measure second-pass duration, *z* = 2.68, *p* = 0.02, with longer reading times at R3 for non-restrictive versus restrictive RCs (see Table 7 below).

At R4, there was a main effect of relativizer for the early measure first-fixation rate, *z* = 6.40, *p* < 0.001, which means that there was a higher probability of fixating R4 when a RC was introduced by *quien* when compared to *que*. This effect was also significant for first fixation, *t* = 7.80, *p* < 0.001, and first-pass duration, *t* = 8.09, *p* < 0.001, confirming longer reading times for *quien* versus *que* in both restrictive and non-restrictive RCs (see Figure 5 above).

Finally, there was also a main effect of relativizer at R4 for the intermediate measure quasi-first-pass time at R4, *t* = 7.83, *p* < 0.001, showing that, after a regression to a previous word and before advancing to the following region, readers fixated the relativizer region for a longer time when a RC was introduced by *quien* in comparison to *que* regardless of RC type (Figure 6).

As for R5, there was a marginal interaction of RC type x relativizer for the early measure first-pass duration, *t* = 2.16, *p* = 0.09, since restrictive RCs received longer fixations when they were introduced by *que* in comparison to *quien* (Table 7); in contrast, non-restrictive RCs showed slightly longer reading times when introduced by *quien* versus *que*, though this latter comparison was not significant (*p* > 0.1). This interaction did reach significance for first-pass regression, *z* = 3.38, *p* < 0.001, quasi-first-pass time, *t* = 3.24, *p* = 0.001, and second-pass duration, *t* = 2.32, *p* = 0.02, meaning that there was a higher probability of regressing from R5 to previous words, as well as longer (re)fixations on R5 when a restrictive RC was introduced by *que* versus *quien*; however, there were no significant differences for non-restrictive RCs (*p* > 0.1).

In sum, R3 exhibited a higher probability of regressing from the head noun to previous words for non-restrictive RCs in comparison to restrictive RCs, the same pattern that we found in the previous contrast *que* versus *el cual*. At R4 there were no differences between the two types of RCs, but reading times increased when a RC of any type was introduced by *quien* when compared to *que* in early measures such as first fixation or first-pass duration. Similarly, readers tended to make more regressions and longer refixations on the relativizer region before advancing to the following word (i.e., R5) when it was occupied by *quien* versus *que*. Nevertheless, once readers reached R5, they exhibited a different pattern of eye movements, since now, restrictive RCs introduced by *que* received longer reading times when compared to the other three conditions in early (though only marginally), intermediate and late measures.

#### 4.2.5. Eye-Tracking Results for Que versus Donde

**Figure 8:**
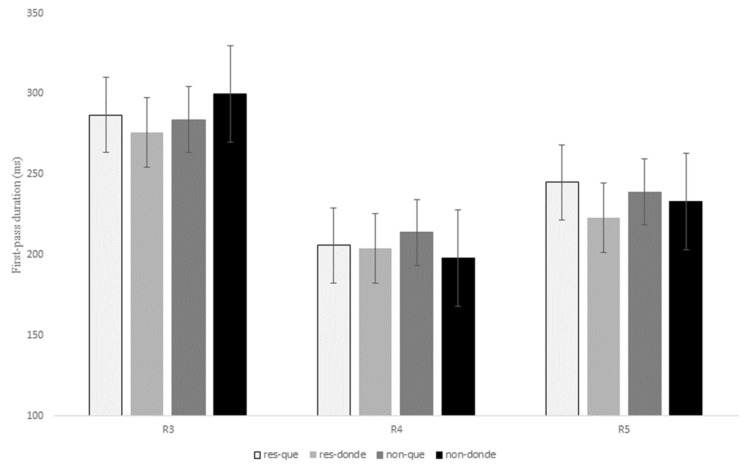
Reading time in milliseconds for the first-pass duration at regions 3, 4, and 5 for the contrast *que* versus *donde* in restrictive and non-restrictive RCs.

**Figure 9:**
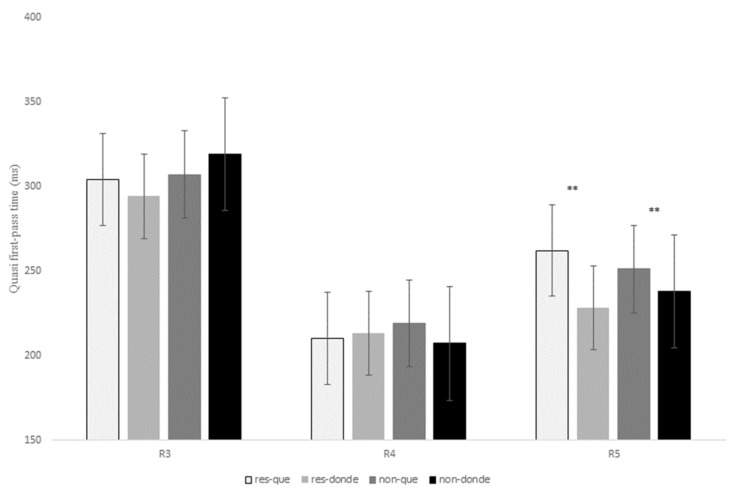
Reading time in milliseconds for the quasi-first-pass reading time at regions 3, 4, and 5 for the contrast of *que* versus *donde* in restrictive and non-restrictive RCs. Asterisks indicate that an effect is significant at *p* < 0.01.

**Figure 10:**
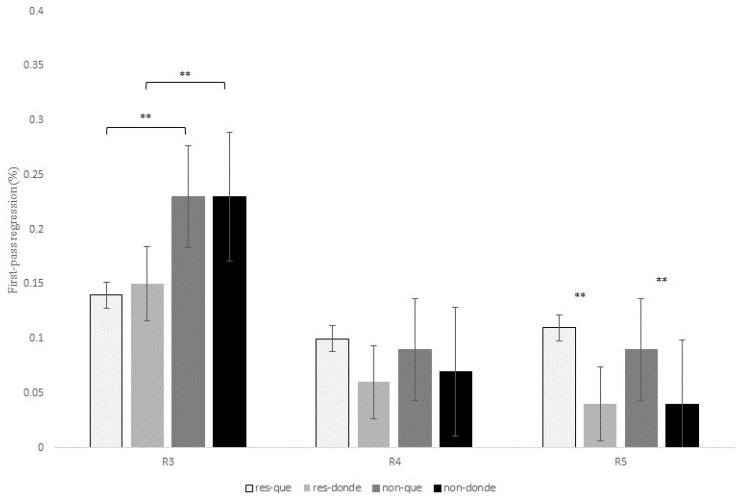
Probability of regression during the first-pass at regions 3, 4, and 5 for the contrast *que* versus *donde* in restrictive and non-restrictive RCs. Asterisks indicate that an effect is significant at *p* < 0.01.

A full presentation of the statistical effects for this contrast is given in Table 8, and displayed for first-pass duration, quasi-first-pass reading time and first-pass regression in Figure 8, Figure 9 and Figure 10 below (see Tables in Section F of Supplementary Materials for models’ reports and for mean reading times of all the eye-tracking measures).

Similar to what was observed in the two previous relativizer contrasts, there was a marginal interaction of RC type x relativizer at R3 for the early measures first fixation, *t* = −2.23, *p* = 0.05, and first-pass duration, *t* = −2.10, *p* = 0.07, which showed longer reading times for non-restrictive RCs in comparison to restrictive RCs when introduced by the relativizer *donde*; however, for sentences with *que* restrictive RCs exhibited slightly longer reading times than non-restrictive RCs (see Table 8 and Figure 8 above).

Additionally, similarly to the previous contrasts, there was a main effect of RC type at R3 for the intermediate measure first-pass regression, *z* = 3.47, *p* = 0.001, since non-restrictive RCs exhibited a higher probability of making a regression from the head noun to previous words in comparison to restrictive RCs (22% versus 14%).

On the other hand, at R4 there was a marginal interaction of RC type x relativizer for first fixation, *t* = 2.17, *p* = 0.09, for although *que* received longer reading times when compared to *donde* for both types of RCs, this difference proved to be larger for non-restrictive RCs (23.33 ms versus 5.62 ms). No other effects were found for the remaining eye movement measures (*p* > 0.1).

As for R5, there was a marginal effect of relativizer for first-pass duration, *t* = 2.16, *p* = 0.09, since the RC verb received longer fixations during the first pass when following *que* versus *donde*. This main effect reached significance for first-pass regression, *z* = 3.40, *p* = 0.001, which indicates that there was a higher probability of making a regression from the RC verb to previous words when a RC was introduced by *que* when compared to *donde* for both types of RCs (Figure 10). This effect was also significant for the intermediate measure quasi-first-pass time, *t* = 3.00, *p* = 0.008 (Figure 9), and the late measure second-pass duration, *t* = 2.41, *p* = 0.04, showing that, after a regression, readers fixated for a longer time the RC verb when it followed *que* versus *donde* (see Table 8 above).

Finally, no main effect of RC type or interaction of RC type x relativizer was found for any of the eye movement measures at R5 (*p* > 0.1).

To sum up, R3 exhibited longer first fixations for non-restrictive RCs with *donde* and for restrictive RCs with *que*, although these differences were only marginal. In addition, readers made more regressions from the head noun (i.e., R3) to previous words for non-restrictive RCs in comparison to restrictive RCs. Unlike the two previous contrasts, once they reached R4, the relativizer region showed longer first fixations when it was occupied by *que* versus *donde*, especially for non-restrictive RCs. Finally, and similarly to the contrast *que* versus *quien*, R5 received longer reading times after a regression when it followed *que* in contrast to *donde* for both types of RCs.

## 5. Discussion

The following discussion of the results reported in the previous section will focus on the effects of each independent variable manipulated in our study (relativizer and RC type) on the relevant eye-tracking measures and also in relation to the data from the corpus study previously reported.

### 5.1. Effect of Relativizer

The results showed a main effect of relativizers at both R4 and R5 for different eye movement measures in all contrasts tested in our experiment. These eye movement measures reflect different aspects of sentence processing, which we will address in turn.

#### 5.1.1. Lexical Activation

First of all, in the contrasts *que* versus *el cual* and *que* versus *quien*, there was a main effect of relativizer at R4 for first fixation and first-fixation rate, measures commonly related to lexical activation. Longer first fixations for *el cual* (“which”) and *quien* (“who”) therefore suggest that these relativizers were more costly to activate than *que* (“that”). Corpus studies showed that the former relativizers were less frequent than the latter, so this higher activation cost might be caused by their lower lexical frequency rate [53,86,87]. In addition, these results were common to both restrictive and non-restrictive RCs, showing that lexical activation cost was consistent across RC type. Nevertheless, in the contrast *que* versus *quien*, the conditions differ not only in relativizers’ lexical frequency but also in their semantic features, so it could be argued that longer first fixations for *quien* versus *que* might be caused by a greater cost when activating the semantic features associated with the former relativizer. Despite the fact that both lexical frequency and semantic feature accounts are compatible, the former seems to have more credence, as the contrast *que* versus *el cual* also showed a main effect of relativizer, and we should recall that these two relativizers lack semantic features. Thus, if lexical frequency determines the activation of *que* and *el cual*, it should also play a role when activating *quien*, regardless of whether semantic features influence its activation as well.

In regard to these results, we could ask whether readers activated relativizers only once they fixated R4 or this process began earlier while fixating R3, that is, whether or not there was parafoveal processing. The parafoveal hypothesis implies that some features of the relativizers were activated while readers were fixating R3, so once they moved forward to R4, the activation process was somehow alleviated. If this hypothesis were true, it should be expected that parafoveal processing would benefit from lexical frequency [88,89]: The fact that *que* is more frequent might have facilitated its lexical activation in the parafovea, so as readers moved to R4, they would not need to fixate it for such a long time, yielding shorter first fixations for *que* versus *el cual* and *quien*. Moreover, parafoveal processing normally involves an increase in reading times at the previous region, that is, the region where parafoveal processing takes place. However, according to our data, there was no effect of relativizer at R3 for the contrasts *que* versus *el cual* and *que* versus *quien*, which makes it difficult to ascertain whether relativizers could be activated foveally at R4 or parafoveally at R3 (at least, some of their features). In this respect, results for the contrast *que* versus *donde* may help clarify this issue.

For the contrast *que* versus *donde*, we found opposite patterns of results at regions 3 and 4 across RC types. Thus, at R3 non-restrictive RCs exhibited longer reading times when the following relativizer was *donde* versus *que*, while the opposite was true for restrictive RCs. As for R4, non-restrictive RCs showed longer reading time measures for sentences headed by *que* versus *donde*. Thus, taking into account the results at regions 3 and 4 together, we could argue for parafoveal processing only in non-restrictive RCs: In this type of RCs, readers spent more time fixating R3 when the following relativizer was *donde* versus *que*, so they gained a more effective preview of the former relativizer than the latter. Consequently, when they moved to R4, fixations at *donde* were shorter in comparison to *que* as it could have been activated, at least partially, at R3. This does not mean that *que* was not activated parafoveally, but its activation level would have been lower, requiring more time to be fully activated later at R4. Nevertheless, these results need to be taken with caution. Firstly, we may ask why evidence in favor of parafoveal processing only shows up in non-restrictive RCs, but not in restrictive RCs: In the latter *que* exhibited longer reading times than *donde* at R3, as well as R4, which downplays the role of parafoveal processing; if readers fixated R3 longer for the *que* condition because they were parafoveally processing this relativizer, we should then expect a decrease in reading times for *que* at R4, which did not occur in our data. Secondly, differences for the contrast *que* versus *donde* were only marginal at both R3 and R4, so new studies are needed to further explore this issue. Finally, previous studies have provided inconsistent evidence about the influence of lexical features on parafoveal word processing: While few studies confirm that reading times on word *n* may vary as a function of the frequency of *n* + 1 [90], most of them deny this possibility [91,92,93,94,95,96]. As previously pointed out, this does not mean there is no parafoveal processing of *n* + 1; however, the influence of lexical variables over this phenomenon seems to be mainly reflected at *n* + 1 rather than at word *n*.

In sum, the eye-tracking measures usually related to lexical activation, i.e., first-fixation rate and first fixation, show differences in the processing of relativizers for the contrasts *que* versus *el cual* and *que* versus *quien*; hence, the relativizer with higher frequency rates (*que*) exhibited shorter reading times than the ones with lower frequency rates (*el cual* and *quien*). These results suggest that the activation of function words such as relativizers is governed, among other features, by their frequency [53,86,87]. Additionally, these results could be interpreted in terms of an influence of either absolute or relative frequency, as *que* is more frequent than *el cual* and *quien* together but also in each individual contrast [52]. Finally, it seems more likely that the influence of lexical frequency upon relativizers’ activation occurs at the relativizer region (that is, foveally), and is not mainly dependent on parafoveal processing.

#### 5.1.2. Syntactic Integration

The main effect of relativizer was also significant at R4 for early and intermediate eye movement measures—first-pass duration and quasi-first-pass reading time—in the contrast *que* versus *el cual* and *que* versus *quien*. First-pass duration shows that, after a first fixation, readers fixate again the target region before exiting it—either in a regressive or progressive manner. Similarly, quasi-first-pass reading time shows that, after a first fixation, readers regress to previous words and then re-fixate the target region before proceeding to subsequent words. Therefore, these measures are generally related to a syntactic integration process: Once a word is activated, readers try to establish a syntactic relationship between it and previous words, leading to new fixations or to refixations when this integration process becomes more difficult [55,56,97]. The results showed longer reading times at R4 for these measures when a RC was introduced by *el cual* or *quien* versus *que*. As shown in the Section 2, the syntactic structure of the experimental items was the same across the four conditions, so longer reading times cannot be explained by a difference in their syntactic structure. The only common difference between conditions lies in the introducing relativizer, and more specifically, in its lexical frequency. Therefore, it seems that establishing a syntactic relationship between a relativizer and previous words is more costly when this unit has a lower lexical frequency rate (*el cual, quien*), leading to longer (re)fixations. These results indicate that lexical frequency related to function words may also influence sentence processing from relatively early stages. Interestingly, this pattern was common to both restrictive and non-restrictive RCs, which indicates that the influence of lexical frequency when integrating a relativizer into a syntactic structure is systematic regardless of RC type [52].

At R5 (that is, the RC verb region), there was also a main effect of relativizer for the contrasts *que* versus *quien* and *que* versus *donde*, which showed that now RC verbs received longer fixations after the relativizer *que* versus *quien* and *donde* for both intermediate and late measures—quasi-first-pass reading time, first-pass regression, and second-pass duration. These results would mean that establishing a syntactic relationship between the RC verb and previous words is more costly when this verb comes after relativizer *que* versus *quien* and *donde*. These results cannot be explained by the relativizers’ lexical frequency, as the opposite pattern should then have ensued (that is, longer reading times, at least after *quien*). Therefore, now differences between conditions might be caused by the semantic features of the relativizers: *quien* and *donde* possess semantic features, while *que* lacks them. These semantic features are shared with the head noun of the RC, so they might have helped to recognize the referent to which the RC verb alluded and, in consequence, to establish a syntactic relationship between these words. Interestingly, for the contrast *que* versus *quien*, longer reading times at the RC verb (i.e., R5) after *que* versus *quien* were only significant for restrictive RCs, and we should recall that the function of this type of RCs is, precisely, to identify the referent of the head noun. Hence, the results at R5 suggest that semantic features related to function words may also guide sentence processing and alleviate its related cost. This hypothesis is not inconsistent with the fact that effects at R5 for the contrast *que* versus *el cual* exhibited the opposite pattern, since neither of these relativizers possesses semantic features (and, therefore, the lexical frequency would keep its influence over RC processing).

Alternatively, the effect brought about by relativizers with semantic features might arise from the adjacent position of the relativizer to the RC verb, as shown by the fact that this facilitatory effect vanishes when an intervening constituent is placed between the constituents with overlapping features [98]. This alternative explanation casts doubt on claims of the influence of semantic features of words on parsing, so there remains an open question for future research whether this alleged semantic effect would remain long enough to facilitate retrieval of the antecedent NP of the RC when disrupted by an intervening constituent. Still, early on in this section, we acknowledged that the lexical frequency in and of itself may account for the immediate effects of the relativizer on RC processing, given the robust effects of semantically underspecified relativizers such as *que* and *el cual*.

### 5.2. Effect of RC Type

Our results show a main effect of RC type at R3 (that is, the head noun) for first-pass regression. This effect was significant across all conditions in the three contrasts, meaning that readers tended to make more regressions from R3 to previous words (and before proceeding to following words) in non-restrictive RCs than in restrictive RCs. This pattern indicates a higher processing cost associated with the head noun of non-restrictive RCs, which could be caused by two factors, not necessarily incompatible. On the one hand, the head noun of non-restrictive RCs was always followed by a comma in contrast to the head noun of restrictive RCs. Previous studies have shown that punctuated words normally exhibit longer reading times than non-punctuated words [65,66,99], so the higher probability of making a regression from the head noun of non-restrictive RCs could be related to the presence of this punctuation mark. Interestingly, our results agree with Grodner et al.’s [64], who also recorded an advantage for non-restrictive RCs in English, but may contradict somehow Hirotani et al.’s results [66]. These authors also compared restrictive versus non-restrictive RCs in English but did not obtain a significant difference between these two types of RCs. Nevertheless, it is important to note that they did not analyze the eye-tracking measure first-pass regression; on the contrary, they focused on other reading measures (first-pass reading time, quasi-first pass reading time and total reading time), and for these measures, neither did we obtain significant results.

At this point, we must recall that the presence of a comma after the head noun of non-restrictive RCs is justified by the role they play. As mentioned in Section 2.3, non-restrictive RCs add an extra meaning to the referent of the head noun, which is already known; in contrast, restrictive RCs are used to identify the referent of the head noun. Therefore, the antecedent of non-restrictive RCs (but not that of restrictive RCs) needs to be identified at R3, and this process could entail a higher cost and, hence, a higher probability of regressing from this region to previous words. In this sense, we could interpret these results as a wrap-up effect—that is, a processing cost associated with syntactic and semantic integration at the end of a clause or sentence [66]. In non-restrictive RCs, readers need to compute a semantic representation of the main clause (and, therefore, of the head noun) at R3, but in restrictive RCs, this process is done while reading the RC itself. In order to substantiate this account, we performed an additional analysis over the total reading times—that is, the sum of all the fixations on a region during both the first and second pass [56]—of R4, R5, and R6, which correspond to the whole structure of the RC. Linear mixed-effect models were built with the RC type, relativizer and their two-way interaction as fixed effects. Random slopes for the RC type and relativizer were also included by subject and by items. As the dependent variable, we analyzed the total reading time over R4, R5, and R6. Linear mixed-effect models were compared conducting likelihood-ratio tests with ANOVA in R, and the *p*-values were adjusted using the Holm–Bonferroni method (the tables in Section G of the Supplementary Materials show a full description of the models’ reports). Table 9 shows the mean reading times for the eye-tracking measure total reading time in the three contrasts.

As can be seen in Table 9, restrictive RCs exhibit longer reading times than non-restrictive RCs when considering all three regions of the RC. This effect was marginal for the contrasts *que* versus *el cual*, *t* = −2.3, *p* = 0.06, and significant for *que* versus *quien*, *t* = −4.19, *p* < 0.001; for the third contrast (*que* versus *donde*), restrictive RCs also show longer reading times than non-restrictive RCs, but this difference did not reach statistical significance (*p* > 0.1). These results suggest that restrictive RCs seem to be harder to process than non-restrictive RCs when reading the full RC, and this extra processing load could also result from a wrap-up effect—that is, readers were computing a syntactic and semantic representation of the restrictive RCs at this point. Obviously, it could be argued that the structure of non-restrictive RCs also needs to be computed at the very end of the RC (e.g., R6); however, and in contrast to restrictive RCs, a part of the structure of non-restrictive RCs has already been computed at R3, thus reducing its processing cost. Taken together, these results suggest that restrictive and non-restrictive RCs are somehow processed in a different way as readers seem to compute a syntactic and semantic structure of each type of RCs at different regions.

To understand this difference, we should also pay attention to the effect caused by punctuated words, which would lead us to reevaluate the parafoveal hypothesis examined and dismissed in the previous section. However, we will reexamine it now with regard to the role of RC types. From this point of view, the claim that the effect of RC type at R3 reflects parafoveal processing—i.e., that readers spend more time at R3 of non-restrictive RCs because they are also processing R4, though plausible, can be ruled out on several grounds. Firstly, parafoveal processing is normally observed in reading measures that report duration data (especially, in early reading measures), but in our study, this effect was significant in first-pass regression—that is, the probability of regressing backward before moving to the next region. Therefore, it does not seem reasonable to relate parafoveal processing of R4 to a measure associated with looking backwards to previous words. Secondly, if readers benefited more from parafoveal processing of R4 when fixating R3 in non-restrictive RCs, it would then be expected that R4 should exhibit shorter reading times for non-restrictive RCs in comparison to restrictive RCs. This effect was significant for the contrast *que* versus *el cual*, where R4 showed shorter reading times for non-restrictive versus restrictive RCs, but it did not yield significant differences for the other two contrasts (*que* versus *quien*, and *que* versus *donde*; see Section 4.2.4 and Section 4.2.5, respectively). If parafoveal processing were the core cause behind the effect of RC type at R3, then the same effect at R4 ought to show up in all contrasts, and not only in one of them. Finally, it could be questioned why readers benefited more from parafoveal processing in non-restrictive RCs than in restrictive RCs, since R3 in non-restrictive RCs always displays a final comma, and this punctuation mark might prevent, rather than promote, parafoveal processing.

On the other hand, in Section 2, we also considered the hypothesis that RC processing may be determined by the frequency rates of RC types. Our corpus study showed that non-restrictive RCs are more frequent than restrictive RCs for the contrasts *que* versus *el cual* and *que* versus *quien*; as for the contrast *que* versus *donde*, there are differences between the two relativizers: Restrictive RCs are more frequent than non-restrictive ones when headed by *que*, whereas non-restrictive RCs are more frequent than restrictive ones when headed by *donde*. The effect of RC type found at R3 cannot be explained by the frequency of RC types because at this point participants have not started reading the RC and therefore cannot know its type. On the contrary, the effect of RC type found at R4, R5, and R6 together for the total reading time could be related to frequency rates, at least for the contrasts *que* versus *el cual* and *que* versus *quien*, as restrictive RCs were less frequent, thus yielding longer reading times (see Table 9). As for *que* versus *donde*, there were no significant differences between the two RC types in any eye-tracking measure, which obviously raises the question why, for this contrast, the frequency rates do not seem to play a role. This latter result downplays the explanation of the effect of RC type being caused by structural frequency patterns.

In sum, our results show that there are processing differences between the two types of RCs: Non-restrictive RCs are harder to process at the head noun region (i.e., R3), while restrictive RCs exhibit a higher processing load at the RC itself (i.e., R4, R5, and R6), at least for two contrasts: *que* versus *el cual* and *que* versus *quien*. Similar results were reported in the study by Grodner et al. [64] described above (see Section 2.3), where a processing advantage for non-restrictive over restrictive RCs was found at the RC verb region when the sentences were presented in isolation, though this study did not test RCs with different relativizers but only the contrast between *that* (in restrictive RCs) versus *who* (in non-restrictive RCs), which somehow parallels the *que* versus *quien* contrast in Spanish. Our results could be explained by the fact that readers compute a representation of the sentence structure at different regions depending on RC type. In non-restrictive RCs, the comma placed after the head noun marks the end of the main clause and therefore the need to establish its syntactic and semantic representation at R3, which might explain the higher probability of making a regression from this region in non-restrictive versus restrictive RCs. On the contrary, the process of computing the structure of restrictive RCs seems to take place while reading the RC itself, yielding longer reading times for restrictive versus non-restrictive RCs at R4, R5, and R6 together. Accordingly, the effects of RC type found in our data seem to reflect *where* along the sentence readers compute a syntactic and semantic interpretation of the clause or sentence.

Obviously, this account is not incompatible with the punctuation hypothesis, according to which the presence of a comma after the head noun of non-restrictive RCs may involve an extra processing cost in comparison to the head noun of restrictive RCs. However, this second hypothesis would only explain the effect of RC type found at R3 for non-restrictive RCs, but it would leave unanswered why restrictive RCs exhibited longer reading times at regions 4, 5, and 6 together. In this regard, we consider that punctuation marks could elicit the identification process associated with non-restrictive RCs at R3, adding some processing load, but this hypothesis by itself cannot explain the effect of RC type found for both restrictive and non-restrictive RCs.

On the other hand, frequency rates could also explain the effect of RC type found at regions 4, 5, and 6 together, since as restrictive RCs are less frequent than non-restrictive RCs (at least for the contrasts *que* versus *el cual* and *que* versus *quien*), they become more difficult to process. Nevertheless, this explanation leaves unanswered why this effect does not arise in the contrast *que* versus *donde* (as restrictive and non-restrictive RCs also exhibit different frequency patterns in this contrast), as well as the effect of RC type found at R3.

Finally, a finding that is hard to accommodate by any of the previous explanations is that the effects of RC type reached statistical significance in different eye-tracking measures. At R3, this effect was significant for first-pass regression, whereas over the whole RC encompassing regions 4, 5, and 6, it was significant for total reading time.

Bringing together the relativizer and RC type effects, we will finally examine our results in the light of the hypotheses put forward by the parsing models reviewed in the opening section of this paper. First, a modular account such as the garden-path theory would have difficulties to account for most of the results reported. On the one hand, this theory would expect no differences in the early processing of RCs due to the heading relativizer and its lexico-semantic features. Therefore, this account cannot explain the effect of relativizer found at different regions. Our results showed differences in both early and intermediate eye-tracking measures, such that RCs introduced by a more frequent relativizer exhibited a lower processing load, both when activating the relativizer and when integrating it into the syntactic structure. In addition, the effect of relativizer at R5 for the contrasts *que* versus *quien* and *que* versus *donde* indicates that the semantic features of the relativizers—where they have—might facilitate sentence processing, so integrating the RC verb with previous constituents is less costly when this verb follows a relativizer that possesses semantic features. Similarly, the garden-path theory would allegedly predict more processing difficulties for non-restrictive RCs, as these RCs are attached to a higher structural level in comparison to restrictive RCs (see example 13 above). It is true that, according to our data, non-restrictive RCs were harder to process than restrictive RCs, but this effect was only significant for first-pass regression at R3, and at this point, participants cannot know they are reading a RC, much less its type. In addition, when considering the structure of the RC (i.e., R4, R5, and R6), the effect of RC type showed longer reading times for restrictive versus non-restrictive RCs, displaying the opposite pattern to the one predicted by the Garden-Path theory.

Alongside the garden-path theory, the Tuning hypothesis would also find it problematic to explain most of the effects of our study. On the one hand, the Tuning hypothesis accepts the influence of coarse-grained frequency at early processing stages, so that those structures that are more frequent would be easier to process. Our corpus study showed that non-restrictive RCs are more frequent than restrictive RCs for the contrasts *que* versus *el cual* and *que* versus *quien* and also for the contrast *que* versus *donde* when these sentences are headed by *donde*. In this regard, the Tuning hypothesis could accommodate the effect of RC type found at R4, R5, and R6, according to which restrictive RCs exhibited longer reading times; however, this account cannot explain why non-restrictive RCs were harder to process at R3. On the other hand, the Tuning hypothesis also leaves unanswered the effect of relativizer found at different regions and for different eye-tracking measures, as this model, similar to the garden-path theory, predicts no differences in sentence processing due to the lexical frequency and the semantic features of the words integrating it, at least at the early stages.

Finally, interactive accounts seem to accommodate most of the effects found in our study. First of all, the effect of relativizer can be interpreted as supporting interactive accounts and, particularly, usage-based accounts. These models claim that those distributional patterns that are more frequent in a language are easier to recognize and, in consequence, to process, which squares with our finding of shorter fixations in early measures for the contrasts *que* versus *el cual* and *que* versus *quien* and the fact that *que* is more frequent (both in absolute and relative terms) [52,67,68,69,70,71]. Similarly, the results at R5 for the contrasts *que* versus *quien* and *que* versus *donde* show that integrating the RC verb with previous constituents is less costly when this verb follows a relativizer that possesses semantic features. These results can be also better accommodated under usage-based accounts, as they claim that the parser records distributional patterns at any linguistic level, so that semantic features may also help to recover the pattern underlying a message. As our results show, semantic features related to function words such as relativizers can also influence the recognition of a pattern and, consequently, the way it is processed. However, we must stress at this point that our results do not support an unconstrained version of interactive models where all extra-syntactic information is brought to bear on the parsing process at once and from the very beginning, for according to our data, the constraints from other sources of information appear to be ranked along a timeline, with the influence of the relativizer frequency coming ahead of that of their semantic features.

Second, the effect of RC type may reflect where along the sentence readers begin to compute a syntactic and semantic interpretation of the sentence. For non-restrictive RCs, this process occurs first at R3—that is, at the end of the main clause, probably sparked by the comma at the end of this region, whereas, for restrictive RCs, it occurs while reading the structure of the RC (R4, R5, and R6). This does not mean that non-restrictive RCs are not computed when reading the structure of the RC (that is, R4, R5, and R6), but in comparison to restrictive RCs, this process seems to be less costly, as a part of the structure of non-restrictive RCs has already been established. In any case, these results suggest that readers are not only considering syntactic information when processing RCs but also their semantic representation, and particularly, this latter information seems to constrain the interpretation of the sentence by showing where certain identification operations need to be completed.

## 6. Conclusions

The aim of our study was to analyze RC processing by taking into account two particular characteristics of these structures in Spanish (and also in other languages): namely, that they can be headed by relativizers with different linguistic features and that they can be of two different types (restrictive or non-restrictive). These questions have been scarcely examined so far in the psycholinguistic literature, hence the exploratory nature of our study. Nevertheless, these questions can be of interest, among other reasons, because they help us to analyze whether non-syntactic information could determine or guide syntactic processing when manipulating certain features of RCs. We were particularly interested in analyzing the influence of two types of non-syntactic information: frequency and semantics. With this aim in mind, we compared, on the one hand, RCs introduced by different relative pronouns, which differ in lexical frequency and/or semantic features, though their variation does not substantially modify the sentence structure nor its general meaning. The second manipulation concerns RC type: restrictive and non-restrictive, as they play different semantic roles regarding the RC head noun and also exhibit different frequency rates.

In the first place, our results show that extra-syntactic information does play a role when computing structure building operations related to RCs. Particularly, the effect of relativizer shows that RC processing differed among conditions, being less costly when the introducing relativizer is more frequent, especially in the relativizer contrasts *que* versus *el cual* and *que* versus *quien* and, to a lesser extent, in the contrast *que* versus *donde*. This pattern was recorded in measures related to lexical activation [53,86,87], as well as related to syntactic integration [51,52,53]. Similarly, there were also differences in the processing of RCs due to the semantic features of the relativizers, such that integrating the RC verb into a sentence was less demanding when following a relativizer that possesses semantic features (*quien* versus *que,* and *donde* versus *que*). Additionally, the effect of RC type found in our data reflects processing difficulties at different regions for restrictive and non-restrictive RCs, which would be caused by the fact that readers are integrating different linguistic information in order to compute the sentence representation. Consequently, our results suggest that both frequency and semantics influence syntactic processing when comprehending RCs in Spanish.

Turning to the order in which this linguistic information becomes available, several of the effects found in our study became significant for early and intermediate eye-tracking measures—that is, before readers had computed the whole structure of the RC. In this regard, our results suggest that lexico-semantic information becomes available at an early processing stage and thus plays a relevant role in the decisions taken by the parser as the sentence unfolds.

Taken together, the results of our experiment seem to be more compatible with an interactive view of language processing [25,43,46,67,100,101], according to which, syntactic processing is not blind to non-syntactic information. On the contrary, non-syntactic information, such as lexical frequency and semantic features, seems to influence and guide syntactic structure building from the beginning and not only at the late stages. Therefore, these results agree with previous studies that have manipulated non-syntactic information related to content words in order to analyze their influence on syntactic processing [31,102,103,104,105]. Thus, the main contribution of our research is the finding that lexico-semantic features of function words are activated at the initial stages of processing and may guide parsing decisions from early on, an issue that has scarcely been investigated so far in the sentence processing literature. Lastly, although the research reported in this paper leaves a few questions open for further research, it provides the first evidence of the online processing of relative clauses based on relativizer and RC-type contrasts in a language with a rich paradigm of relative pronouns such as Spanish.

## Figures and Tables

**Table 1:** Corpus study data by linguistic level. Relative frequency appears in parenthesis. For the total number of relativizers, the relative frequency was calculated over total number of words; for the total number of relativizers in variation contexts, the relative frequency was calculated over the total number of relativizers.

Linguistic Variety	Total Number of Words	Total Number of Relativizers	Total Number of Relativizers in Variation Contexts	Corpus
Oral formal	33,319	1905 (5.72%)	538 (28.24%)	CREA
Oral informal	33,389	1890 (5.66%)	391 (20.69%)	Val.Es.Co and COSER
Written formal	34,862	1812 (5.2%)	790 (43.6%)	CREA
Written informal	32,448	1771 (5.46%)	653 (36.87%)	Blogs

**Table 2:** Absolute and relative frequencies (in parentheses) of relativizers in restrictive and non-restrictive RCs according to the three contrasts of interest: *que* versus *el cual*, *que* versus *quien*, and *que* versus *donde*. Relative frequency was calculated over the total number of relativizers in the contexts where two of them can vary.

Contrast	Relativizer	Restrictive RC	Non-Restrictive RC
*que* versus *el cual*	que	494 (25.89%)	1333 (69.86%)
	cual	34 (1.78%)	47 (2.46%)
*que* versus *quien*	que	13 (2.84%)	374 (81.84%)
	quien	8 (1.75%)	62 (13.57%)
*que* versus *donde*	que	133 (28.85%)	62 (13.45%)
	donde	120 (26.03%)	146 (31.67%)

**Table 3:** Linguistic features of Spanish relativizers according to our corpus study.

Contrast	Relativizer	Lexical Frequency	Semantic Feature
*que* versus *el cual*	que	Higher	No
	el cual	Lower	No
*que* versus *quien*	que	Higher	No
	quien	Lower	Yes
*que* versus *donde*	que	Higher in restrictive RCs	No
	donde	Higher in non-restrictive RCs	Yes

**Table 4:** Division of the experimental items into six regions according to the syntactic structure. “Res” refers to restrictive RCs and “Non” to non-restrictive RCs.

Contrast	Condition	Region 1	Region 2	Region 3	Region 4	Region 5	Region 6
*que* versus *el cual*	Res_Que	Mi madre	perdió	el bolso	en el que	guardaba	las llaves del coche.
	Res_Cual	Mi madre	perdió	el bolso	en el cual	guardaba	las llaves del coche.
	Non_Que	Mi madre	perdió	su bolso	en el que	guardaba	las llaves del coche.
	Non_Cual	Mi madre	perdió	su bolso	en el cual	guardaba	las llaves del coche.
*que* versus *quien*	Res_Que	El entrenador	se enfadó	con el jugador	al que	fichó	al final de la temporada.
	Res_Quien	El entrenador	se enfadó	con el jugador	a quien	fichó	al final de la temporada.
	Non_Que	El entrenador	se enfadó	con su jugador	al que	fichó	al final de la temporada.
	Non_Quien	El entrenador	se enfadó	con su jugador	a quien	fichó	al final de la temporada.
*que* versus *donde*	Res_Que	La policía	registró	el barrio	del que	proceden	los muchachos desaparecidos.
	Res_Donde	La policía	registró	el barrio	de donde	proceden	los muchachos desaparecidos.
	Non_Que	La policía	registró	mi barrio	del que	proceden	los muchachos desaparecidos.
	Non_Donde	La policía	registró	mi barrio	de donde	proceden	los muchachos desaparecidos.

**Table 5:** Mean accuracy rates (%) and *SD* (in parentheses) to comprehension questions by contrast and condition. “Res” refers to restrictive RCs and “Non-Res” to non-restrictive RCs. Similarly, “Other” refers to the relativizer that is compared with *que* in each contrast: *El cual*, *quien,* or *donde*.

Contrast	Res_*Que*	Res_Other	Non-Res_*Que*	Non-Res_Other
*que* versus *el cual*	97.03 (6.85)	95.24 (9.19)	96.58 (7.40)	97.32 (6.33)
*que* versus *quien*	96.58 (6.79)	94.79 (8.06)	95.54 (7.56)	95.39 (7.61)
*que* versus *donde*	96.73 (6.71)	94.94 (9.03)	94.79 (8.32)	96.58 (7.10)

**Table 6:** Estimated values from the linear mixed-effect models and mixed-effect logistic regression for the contrast *que* versus *el cual* at R3, R4, and R5. Eye movement measures are first-fixation rate (FFR), first fixation (FF), first-pass duration (FPD), first-pass regression (FPR), quasi-first-pass reading time (QFP), and second-pass duration (SPD). *t*-values refer to reading time measures, and *z*-values refer to probability measures.

Region	Measure	Factor	Estimated *β*	SD	*t/z*	*p*
R3	FFR	Intercept	3.13	0.31	9.80	<0.001
		RC type	−0.30	0.28	−1.07	0.84
		Relativizer	−0.05	0.27	−0.21	1.00
		RC type x relativizer	0.14	0.30	0.45	1.00
	FF	Intercept	242.68	10.906	22.25	<0.001
		RC type	4.01	7.568	0.53	1.00
		Relativizer	0.99	6.759	0.14	1.00
		RC type x relativizer	−1.30	9.426	−0.13	1.00
	FPD	Intercept	270.78	13.29	20.36	<0.001
		RC type	1.30	9.69	0.13	1.00
		Relativizer	3.19	8.41	0.37	1.00
		RC type x relativizer	−2.22	11.42	−0.19	1.00
	FPR	Intercept	−2.07	0.197	−10.52	<0.001
		RC type	0.50	0.17	2.88	0.01
		Relativizer	0.22	0.17	1.27	0.4
		RC type x relativizer	−0.06	0.21	−0.3	0.76
	QFP	Intercept	285.76	15.45	18.48	<0.001
		RC type	−3.51	10.98	−0.32	1.00
		Relativizer	4.38	9.61	0.45	1.00
		RC type x relativizer	0.92	12.49	0.07	1.00
	SPD	Intercept	55.57	8.91	6.23	<0.001
		RC type	17.59	15.15	1.16	0.73
		Relativizer	11.84	14.84	0.79	0.73
		RC type x relativizer	19.34	17.80	1.08	0.73
R4	FFR	Intercept	3.46	0.31	10.89	<0.001
		RC type	−0.72	0.27	−2.58	0.009
		Relativizer	−1.03	0.26	−3.94	<0.001
		RC type x relativizer	1.08	0.29	3.72	<0.001
	FF	Intercept	281.10	13.14	21.39	<0.001
		RC type	−15.25	7.94	−1.92	0.11
		Relativizer	−51.64	8.48	−6.08	<0.001
		RC type x relativizer	14.38	9.67	1.48	0.13
	FPD	Intercept	299.52	14.65	20.44	<0.001
		RC type	−22.90	8.73	−2.62	0.017
		Relativizer	−61.68	9.22	−6.68	<0.001
		RC type x relativizer	19.40	10.46	1.85	0.06
	FPR	Intercept	−1.69	0.19	−8.83	<0.001
		RC type	−0.65	0.20	−3.10	0.003
		Relativizer	−0.60	0.18	−3.26	0.003
		RC type x relativizer	0.45	0.24	1.86	0.06
	QFP	Intercept	309.75	16.14	19.19	<0.001
		RC type	−26.83	9.05	−2.96	0.006
		Relativizer	−68.06	9.72	−6.99	<0.001
		RC type x relativizer	25.28	10.91	2.31	0.02
	SPD	Intercept	71.15	11.01	6.46	<0.001
		RC type	−24.99	9.58	−2.60	0.01
		Relativizer	−32.48	10.08	−3.22	0.003
		RC type x relativizer	21.89	11.70	1.87	0.06
R5	FFR	Intercept	2.13	0.22	9.66	<0.001
		RC type	0.16	0.21	0.78	0.79
		Relativizer	−0.24	0.20	−1.17	0.71
		RC type x relativizer	−0.19	0.23	−0.84	0.79
	FF	Intercept	184.15	8.63	21.32	<0.001
		RC type	−2.38	5.70	−0.41	1.00
		Relativizer	−4.50	5.19	−0.86	1.00
		RC type x relativizer	−6.29	6.81	−0.92	1.00
	FPD	Intercept	225.74	14.97	15.07	<0.001
		RC type	−2.45	8.60	−0.28	0.88
		Relativizer	6.44	8.34	0.77	0.88
		RC type x relativizer	−17.96	10.35	−1.73	0.24
	FPR	Intercept	−2.47	0.21	−11.26	<0.001
		RC type	−0.54	0.24	−2.28	0.06
		Relativizer	−0.05	0.22	−0.22	0.82
		RC type x relativizer	0.40	0.29	1.37	0.33
	QFP	Intercept	242.69	17.52	13.84	<0.001
		RC type	−8.68	9.66	−0.89	1.00
		Relativizer	−0.06	9.10	−0.007	1.00
		RC type x relativizer	−9.88	11.60	−0.85	1.00
	SPD	Intercept	66.14	10.84	6.10	<0.001
		RC type	−28.88	12.33	−2.34	0.05
		Relativizer	−22.07	12.12	−1.82	0.13
		RC type x relativizer	27.55	15.86	1.73	0.13

**Table 7:** Estimated values from the linear mixed-effect models and mixed-effect logistic regression for the contrast *que* versus *quien* at R3, R4, and R5. Eye movement measures are first-fixation rate (FFR), first fixation (FF), first-pass duration (FPD), first-pass regression (FPR), quasi-first-pass reading time (QFP), and second-pass duration (SPD). *t*-values refer to reading time measures, and *z*-values refer to probability measures.

Region	Measure	Factor	Estimated *β*	SD	*t/z*	*p*
R3	FFR	Intercept	28.4	0.23	11.92	<0.001
		RC type	0.47	0.29	1.60	0.32
		Relativizer	−0.004	0.26	−0.01	0.98
		RC type x relativizer	0.49	0.34	−1.43	0.32
	FF	Intercept	264.76	8.88	29.81	<0.001
		RC type	12.03	7.82	1.53	0.34
		Relativizer	−12.43	7.88	−1.57	0.34
		RC type x relativizer	6.97	10.47	0.66	0.50
	FPD	Intercept	297.77	11.07	26.88	<0.001
		RC type	9.44	9.47	0.99	0.63
		Relativizer	−17.95	9.53	−1.88	0.17
		RC type x relativizer	12.20	12.40	0.98	0.63
	FPR	Intercept	−1.90	0.17	−10.78	<0.001
		RC type	0.65	0.18	3.64	<0.001
		Relativizer	−0.02	0.17	−0.13	0.89
		RC type x relativizer	0.16	0.20	0.81	0.83
	QFP	Intercept	308.31	12.37	24.91	<0.001
		RC type	16.29	10.12	1.60	0.32
		Relativizer	−11.71	10.00	−1.17	0.48
		RC type x relativizer	6.46	13.25	0.48	0.62
	SPD	Intercept	62.34	9.15	6.81	<0.001
		RC type	49.14	18.29	2.68	0.02
		Relativizer	9.05	14.84	0.61	1.00
		RC type x relativizer	1.11	18.30	0.06	1.00
R4	FFR	Intercept	0.95	0.20	4.66	<0.001
		RC type	0.14	0.14	0.97	0.33
		Relativizer	1.15	0.17	6.40	<0.001
		RC type x relativizer	−0.33	0.21	−1.60	0.21
	FF	Intercept	156.47	10.29	15.19	<0.001
		RC type	2.71	6.61	0.41	0.68
		Relativizer	50.81	6.51	7.80	<0.001
		RC type x relativizer	−8.27	8.11	−1.01	0.61
	FPD	Intercept	168.65	11.71	14.39	<0.001
		RC type	−0.79	7.41	−0.10	0.91
		Relativizer	57.68	7.12	8.09	<0.001
		RC type x relativizer	−14.05	9.17	−1.53	0.25
	FPR	Intercept	−2.87	0.23	−12.21	<0.001
		RC type	−0.35	0.31	−1.13	0.77
		Relativizer	−0.14	0.30	−0.47	1.00
		RC type x relativizer	0.21	0.38	0.56	1.00
	QFP	Intercept	172.74	12.10	14.26	<0.001
		RC type	−2.50	7.94	−0.31	0.75
		Relativizer	60.60	7.73	7.83	<0.001
		RC type x relativizer	−15.77	9.64	−1.63	0.20
	SPD	Intercept	25.29	6.25	4.04	<0.001
		RC type	0.74	9.21	0.08	0.93
		Relativizer	7.73	9.76	0.79	0.85
		RC type x relativizer	−12.24	10.99	−1.11	0.79
R5	FFR	Intercept	2.34	0.21	19.78	<0.001
		RC type	−0.32	0.19	−1.67	0.28
		Relativizer	−0.19	0.19	−0.96	0.33
		RC type x relativizer	0.39	0.24	1.62	0.28
	FF	Intercept	191.36	7.25	26.36	<0.001
		RC type	−9.51	4.88	−1.94	0.15
		Relativizer	1.86	5.19	0.35	0.96
		RC type x relativizer	4.58	6.55	0.69	0.96
	FPD	Intercept	251.72	12.55	20.05	<0.001
		RC type	−16.93	8.28	−2.04	0.09
		Relativizer	−18.00	8.50	−2.11	0.09
		RC type x relativizer	23.05	10.67	2.16	0.09
	FPR	Intercept	−1.85	0.18	−10.28	<0.001
		RC type	−1.20	0.28	−4.29	<0.001
		Relativizer	−1.13	0.26	−4.33	<0.001
		RC type x relativizer	1.22	0.36	3.38	<0.001
	QFP	Intercept	280.07	14.05	19.93	<0.001
		RC type	−36.37	10.02	−3.62	<0.001
		Relativizer	−33.97	9.10	−3.73	<0.001
		RC type x relativizer	38.46	11.84	3.24	0.001
	SPD	Intercept	90.34	11.83	7.63	<0.001
		RC type	−57.13	12.87	−4.44	<0.001
		Relativizer	−38.24	12.01	−3.18	0.002
		RC type x relativizer	38.43	16.53	2.32	0.02

**Table 8:** Estimated values from the linear mixed-effect models and mixed-effect logistic regression for the contrast *que* versus *donde* at R3, R4, and R5. Eye movement measures are first-fixation rate (FFR), first fixation (FF), first-pass duration (FPD), first-pass regression (FPR), quasi-first-pass reading time (QFP), and second-pass duration (SPD). *t*-values refer to reading time measures and *z*-values refer to probability measures.

Region	Measure	Factor	Estimated *β*	SD	*t/z*	*p*
R3	FFR	Intercept	2.69	0.19	13.62	<0.001
		RC type	0.11	0.25	0.45	1.00
		Relativizer	0.08	0.24	0.34	1.00
		RC type x relativizer	−0.10	0.30	−0.36	1.00
	FF	Intercept	239.18	8.07	29.61	<0.001
		RC type	19.76	7.65	2.58	0.02
		Relativizer	9.54	6.79	1.40	0.16
		RC type x relativizer	−21.00	9.39	−2.23	0.05
	FPD	Intercept	275.45	13.24	20.79	<0.001
		RC type	24.26	10.92	2.22	0.07
		Relativizer	11.60	9.16	1.26	0.20
		RC type x relativizer	−27.22	12.96	−2.10	0.07
	FPR	Intercept	−1.96	0.17	−10.91	<0.001
		RC type	0.56	0.16	3.47	0.001
		Relativizer	−0.07	0.19	−0.39	1.00
		RC type x relativizer	0.03	0.21	0.17	1.00
	QFP	Intercept	293.65	15.43	19.03	<0.001
		RC type	24.94	12.31	2.02	0.12
		Relativizer	10.58	10.49	1.00	0.31
		RC type x relativizer	−22.53	14.15	−1.59	0.22
	SPD	Intercept	65.36	9.31	7.01	<0.001
		RC type	33.14	16.00	2.07	0.11
		Relativizer	4.53	13.87	0.32	1.00
		RC type x relativizer	−0.01	17.44	−0.001	1.00
R4	FFR	Intercept	1.95	0.19	10.06	<0.001
		RC type	−0.17	0.17	−1.01	0.62
		Relativizer	−0.19	0.23	−0.84	0.62
		RC type x relativizer	0.28	0.22	1.28	0.59
	FF	Intercept	190.21	7.55	25.16	<0.001
		RC type	−6.82	5.86	−1.16	0.49
		Relativizer	5.62	9.77	0.57	0.56
		RC type x relativizer	17.61	8.11	2.17	0.09
	FPD	Intercept	203.56	9.15	22.24	<0.001
		RC type	−5.45	6.78	−0.80	0.84
		Relativizer	2.01	10.21	0.19	0.84
		RC type x relativizer	13.20	9.00	1.46	0.42
	FPR	Intercept	−3.14	0.26	−11.95	<0.001
		RC type	0.34	0.27	1.25	0.41
		Relativizer	0.54	0.26	2.09	0.11
		RC type x relativizer	−0.22	0.31	−0.70	0.48
	QFP	Intercept	212.83	9.42	22.59	<0.001
		RC type	−6.16	7.34	−0.84	0.80
		Relativizer	−2.69	10.39	−0.26	0.80
		RC type x relativizer	14.67	9.73	1.50	0.39
	SPD	Intercept	30.47	8.28	3.67	<0.001
		RC type	11.89	12.01	0.99	0.96
		Relativizer	6.51	11.18	0.58	1.00
		RC type x relativizer	−7.38	14.57	−0.50	1.00
R5	FFR	Intercept	2.15	0.20	10.57	<0.001
		RC type	−0.10	0.19	−0.54	1.00
		Relativizer	−0.01	0.19	−0.07	1.00
		RC type x relativizer	0.02	0.23	0.08	1.00
	FF	Intercept	188.50	7.31	25.78	<0.001
		RC type	−1.57	5.83	−0.26	1.00
		Relativizer	1.28	5.42	0.23	1.00
		RC type x relativizer	−7.20	6.64	−1.08	0.83
	FPD	Intercept	223.20	13.57	16.44	<0.001
		RC type	10.17	8.08	1.25	0.26
		Relativizer	21.90	10.13	2.16	0.09
		RC type x relativizer	−16.57	11.02	−1.50	0.26
	FPR	Intercept	−3.40	0.27	−12.21	<0.001
		RC type	0.07	0.35	0.19	0.89
		Relativizer	1.02	0.30	3.40	0.001
		RC type x relativizer	−0.29	0.39	−0.75	0.89
	QFP	Intercept	228.20	14.46	15.77	<0.001
		RC type	9.68	8.80	1.10	0.27
		Relativizer	33.84	11.27	3.00	0.008
		RC type x relativizer	−20.62	11.53	−1.78	0.14
	SPD	Intercept	27.83	8.05	3.45	0.002
		RC type	−0.90	11.86	−0.07	1.00
		Relativizer	31.49	13.05	2.41	0.04
		RC type x relativizer	2.24	15.90	0.14	1.00

**Table 9:** Mean total reading times (ms) by condition over R4, R5, and R6 for the three contrasts. The numbers in parentheses indicate the standard deviation.

Contrast	RESTRICTIVE RCs		NON-RESTRICTIVE RCs	
	QUE	OTHER	QUE	OTHER
*que* versus *el cual*	525.28 (242.17)	556.45 (224.63)	482.03 (229.80)	520.47 (238.93)
*que* versus *quien*	542.51 (274.79)	534.50 (253.47)	488.12 (271.72)	508.76 (264.02)
*que* versus *donde*	536.34 (213.35)	510.81 (237.39)	516.67 (213.83)	504.99 (246.04)

## Data Availability

The data that support the findings of this study are available from the corresponding author, E.Á.-G., upon request.

## Supplementary Materials

The following supporting information can be downloaded at: https://www.mdpi.com/article/10.3390/brainsci13030409/s1.
