# Supplementary material for: Lexico-Semantic Influence on Syntactic Processing: An Eye-Tracking Study with Spanish Relative Clauses
Source: Brain Sci. 2023 Feb 26;13(3):409. doi: 10.3390/brainsci13030409 (PMC10046643; doi:10.3390/brainsci13030409)
Supplement: Supplementary file 1 [file brainsci-13-00409-s001.zip › brainsci-2219488-supplementary.pdf]

Supplementary Materials

Section A. Supplementary material of the corpus study

Table S1.1. Models' comparisons using Likelihood-ratio test (LRT) for the relative frequency of *Que* versus *El cual* in restrictive and non-restrictive RCs. Asterisks in the last column indicate a *p*-value below the significance level of 0.001 for *** and below 0.01 for **.

Model's selection

Sampling Units	N total obs = 16<br>N Variety = 4									
**Model specification**	**Model name**	**Nested / simpler Model**	**Fixed Effects added**		**Random Effects**<br>**Linguistic Variety**	**Model fit**<br>**AIC**	**BIC**	**LL**	**LRT Test against nested**<br>**df**	**X2**
	Null	-	-		intercept	2570.48	2572.03	-1283.24		
FE main effects	Main effects	Null	RC type + relativizer		intercept	205.53	208.62	-98.77	2	2368.9***
FE two-way interactions	Two-way interaction	Main effects	RC type + relativizer + RCtype*relativizer		intercept	199.55	203.41	-84.77	1	7.98**

Table S1.2. Results of the linear mixed-effect model for the relative frequency of *Que* versus *El cual*. The top of the table reports estimates/beta values, standard error (SE), confidence intervals (95% CI), *z*-values and levels of significance for fixed effects; the bottom of the table reports variance and standard deviation (S.D.) for the random effect.

Fixed Effects						
	Est/Beta	SE	95% CI		z	p
Intercept	2.11	0.2	1.68	2.52	10.3	< .001
RC type	0.32	0.22	-0.11	0.77	1.43	.15
Relativizer	2.67	0.17	2.34	3.04	15.1	< .001
RC type*relativizer	0.66	0.23	0.2	1.11	2.89	< .01
Random Effect						
		Variance		S.D.		
Linguistic variety (intercept)		0.05		0.22		

Table S2.1. Models' comparisons using Likelihood-ratio test (LRT) for the relative frequency of *Que* versus *Quien* in restrictive and non-restrictive RCs. Asterisks in the last column indicate a *p*-value below the significance level of 0.001 for *** and below 0.01 for **.

Model's selection

Sampling Units	N total obs = 16<br>N Variety = 4									
**Model specification**	**Model name**	**Nested / simpler Model**	**Fixed Effects added**		**Random Effects**	**Model fit**			**LRT Test against nested**	
					**Linguistic Variety**	**AIC**	**BIC**	**LL**	**df**	**X2**
RE only	Null	-	-		intercept	856.15	857.69	-426.07		
FE main effects	Main effects	Null	RC type + relativizer		intercept	154.81	157.9	-73.4	2	705.34***
FE two-way interactions	Two-way interaction	Main effects	RC type + relativizer + RCtype*relativizer		Intercept	149.97	153.83	-69.98	1	6.84**

Table S2.2. Results of the linear mixed-effect model for the relative frequency of *Que* versus *Quien*. The top of the table reports estimates/beta values, standard error (SE), confidence intervals (95% CI), *z*-values and levels of significance for fixed effects; the bottom of the table reports variance and standard deviation (S.D.) for the random effect.

Fixed Effects						
	Est/Beta	SE	95% CI		z	p
Intercept	1.15	0.29	0.53	1.7	3.93	< .001
RC type	3.35	0.28	2.84	3.96	11.92	< .001
Relativizer	-0.48	0.44	-1.41	0.37	-1.08	.27
RC type*relativizer	-1.31	0.46	-2.21	-0.34	-2.79	< .05
Random Effect						
		Variance			S.D.	
Linguistic variety (intercept)		0.03			0.19	

Table S3.1. Models' comparisons using Likelihood-ratio test (LRT) for the relative frequency of *Que* versus *Donde* in restrictive and non-restrictive RCs. The asterisk in the last column represents a *p*-value below the significance level of 0.001.

Model's selection

Sampling Units		N total obs = 16<br>N Variety = 4								
**Model specification**	**Model name**	**Nested / simpler Model**	**Fixed Effects added**		**Random Effects**	**Model fit**			**LRT Test against nested**	
					**Linguistic Variety**	**AIC**	**BIC**	**LL**	**df**	**X2**
RE only	Null	-	-		intercept	169.98	171.53	-82.99		
FE main effects	Main effects	Null	RC type + relativizer		intercept	158.6	161.69	-75.3	2	15.37***
FE two-way interactions	Two-way interaction	Main effects	RC type + relativizer + RCtype*relativizer		intercept	136	139.87	-63	1	24.6***

Table S3.2. Results of the linear mixed-effect model for the relative frequency of *Que* versus *Donde*. The top of the table reports estimates/beta values, standard error (SE), confidence intervals (95% CI), *z*-values and levels of significance for fixed effects; the bottom of the table reports variance and standard deviation (S.D.) for the random effect.

Fixed Effects						
	Est/Beta	SE	95% CI		z	p
Intercept	3.37	0.15	3.01	3.72	22.2	< .001
RC type	0.19	0.12	-0.04	0.43	1.59	.22
Relativizer	0.1	0.12	-0.14	0.35	0.81	.41
RC type*relativizer	-0.95	0.19	-1.34	-0.57	-4.87	< .001
Random Effect						
			Variance		S.D.	
Linguistic variety (intercept)			0.05		0.24	

Section B. Experimental stimuli

1. Experimental stimuli of the contrast *Que* versus *El cual*

**1**	RES-QUE	*El profesor impartió el curso en el que explicaba los problemas en educación.*
	RES-CUAL	*El profesor impartió el curso en el cual explicaba los problemas en educación.*
	NON-QUE	*El profesor impartió un curso, en el que explicaba los problemas en educación.*
	NON-CUAL	*El profesor impartió un curso, en el cual explicaba los problemas en educación.*
**2**	RES-QUE	*Los ministros pactaron la alianza con la que finalizaron la guerra entre sus países.*
	RES-CUAL	*Los ministros pactaron la alianza con la cual finalizaron la guerra entre sus países.*
	NON-QUE	*Los ministros pactaron su alianza, con la que finalizaron la guerra entre sus países.*
	NON-CUAL	*Los ministros pactaron su alianza, con la cual finalizaron la guerra entre sus países.*
**3**	RES-QUE	*El abogado redactó el informe en el que demostraba la inocencia del acusado.*
	RES-CUAL	*El abogado redactó el informe en el cual demostraba la inocencia del acusado.*
	NON-QUE	*El abogado redactó su informe, en el que demostraba la inocencia del acusado.*
	NON-CUAL	*El abogado redactó su informe, en el cual demostraba la inocencia del acusado.*
**4**	RES-QUE	*La revista publicó un artículo en el que describía las tendencias en moda.*
	RES-CUAL	*La revista publicó un artículo en el cual describía las tendencias en moda.*
	NON-QUE	*La revista publicó mi artículo, en el que describo las tendencias en moda.*
	NON-CUAL	*La revista publicó mi artículo, en el cual describo las tendencias en moda.*
**5**	RES-QUE	*La universidad finalizó la reforma para la que contrató una empresa de Alemania.*
	RES-CUAL	*La universidad finalizó la reforma para la cual contrató una empresa de Alemania.*
	NON-QUE	*La universidad finalizó su reforma, para la que contrató una empresa de Alemania.*

	NON-CUAL	*La universidad finalizó su reforma, para la cual contrató una empresa de Alemania.*
**6**	RES-QUE	*El presidente inauguró el barco en el que realizaría su viaje a México.*
	RES-CUAL	*El presidente inauguró el barco en el cual realizaría su viaje a México.*
	NON-QUE	*El presidente inauguró su barco, en el que realizaría su viaje a México.*
	NON-CUAL	*El presidente inauguró su barco, en el cual realizaría su viaje a México.*
**7**	RES-QUE	*Pedro ha olvidado la contraseña con la que abría su cuenta de correo.*
	RES-CUAL	*Pedro ha olvidado la contraseña con la cual abría su cuenta de correo.*
	NON-QUE	*Pedro ha olvidado su contraseña, con la que abría su cuenta de correo.*
	NON-CUAL	*Pedro ha olvidado su contraseña, con la cual abría su cuenta de correo.*
**8**	RES-QUE	*El congresista habló en el idioma en el que tiene una mayor competencia.*
	RES-CUAL	*El congresista habló en el idioma en el cual tiene una mayor competencia.*
	NON-QUE	*El congresista habló en su idioma, en el que tiene una mayor competencia.*
	NON-CUAL	*El congresista habló en su idioma, en el cual tiene una mayor competencia.*
**9**	RES-QUE	*Mi amiga recibió el premio con el que realizará sus estudios de doctorado.*
	RES-CUAL	*Mi amiga recibió el premio con el cual realizará sus estudios de doctorado.*
	NON-QUE	*Mi amiga recibió un premio, con el que realizará sus estudios de doctorado.*
	NON-CUAL	*Mi amiga recibió un premio, con el cual realizará sus estudios de doctorado.*
**10**	RES-QUE	*El cantante compuso la canción con la que abrirá el concierto de mañana.*
	RES-CUAL	*El cantante compuso la canción con la cual abrirá el concierto de mañana.*
	NON-QUE	*El cantante compuso una canción, con la que abrirá el concierto de mañana.*
	NON-CUAL	*El cantante compuso una canción, con la cual abrirá el concierto de mañana.*
**11**	RES-QUE	*El guerrero guardó la espada con la que venció a sus enemigos en la batalla.*
	RES-CUAL	*El guerrero guardó la espada con la cual venció a sus enemigos en la batalla.*
	NON-QUE	*El guerrero guardó su espada, con la que venció a sus enemigos en la batalla.*
	NON-CUAL	*El guerrero guardó su espada, con la cual venció a sus enemigos en la batalla.*
**12**	RES-QUE	*Mi madre perdió el bolso en el que guardaba las llaves del coche.*
	RES-CUAL	*Mi madre perdió el bolso en el cual guardaba las llaves del coche.*
	NON-QUE	*Mi madre perdió su bolso, en el que guardaba las llaves del coche.*
	NON-CUAL	*Mi madre perdió su bolso, en el cual guardaba las llaves del coche.*
**13**	RES-QUE	*El director envió el comunicado en el que dimitía de su puesto en la empresa.*
	RES-CUAL	*El director envió el comunicado en el cual dimitía de su puesto en la empresa.*
	NON-QUE	*El director envió un comunicado, en el que dimitía de su puesto en la empresa.*
	NON-CUAL	*El director envió un comunicado, en el cual dimitía de su puesto en la empresa.*
**14**	RES-QUE	*El profesor inauguró la charla en la que presentó su teoría sobre la gravedad.*
	RES-CUAL	*El profesor inauguró la charla en la cual presentó su teoría sobre la gravedad.*
	NON-QUE	*El profesor inauguró su charla, en la que presentó su teoría sobre la gravedad.*
	NON-CUAL	*El profesor inauguró su charla, en la cual presentó su teoría sobre la gravedad.*
**15**	RES-QUE	*La banda premió al cantante con el que realizó la gira por España.*
	RES-CUAL	*La banda premió al cantante con el cual realizó la gira por España.*
	NON-QUE	*La banda premió a su cantante, con el que realizó la gira por España.*
	NON-CUAL	*La banda premió a su cantante, con el cual realizó la gira por España.*
**16**	RES-QUE	*El partido elegirá al candidato con el que espera ganar las elecciones.*
	RES-CUAL	*El partido elegirá al candidato con el cual espera ganar las elecciones.*
	NON-QUE	*El partido elegirá a su candidato, con el que espera ganar las elecciones.*
	NON-CUAL	*El partido elegirá a su candidato, con el cual espera ganar las elecciones.*

**17**	RES-QUE	*Mi hermano se reunió con el amigo con el que compartirá el piso este año.*
	RES-CUAL	*Mi hermano se reunió con el amigo con el cual compartirá el piso este año.*
	NON-QUE	*Mi hermano se reunió con su amigo, con el que compartirá el piso este año.*
	NON-CUAL	*Mi hermano se reunió con su amigo, con el cual compartirá el piso este año.*
**18**	RES-QUE	*Mi amiga no conocía al profesor con el que haría las prácticas de la asignatura.*
	RES-CUAL	*Mi amiga no conocía al profesor con el cual haría las prácticas de la asignatura.*
	NON-QUE	*Mi amiga no conocía a su profesor, con el que haría las prácticas de la asignatura.*
	NON-CUAL	*Mi amiga no conocía a su profesor, con el cual haría las prácticas de la asignatura.*
**19**	RES-QUE	*El ejército venció al enemigo contra el que libraron las peores batallas.*
	RES-CUAL	*El ejército venció al enemigo contra el cual libraron las peores batallas.*
	NON-QUE	*El ejército venció a su enemigo, contra el que libraron las peores batallas.*
	NON-CUAL	*El ejército venció a su enemigo, contra el cual libraron las peores batallas.*
**20**	RES-QUE	*Alfonso se citó con el abogado con el que colaboró en el juicio contra su jefe.*
	RES-CUAL	*Alfonso se citó con el abogado con el cual colaboró en el juicio contra su jefe.*
	NON-QUE	*Alfonso se citó con su abogado, con el que colaboró en el juicio contra su jefe.*
	NON-CUAL	*Alfonso se citó con su abogado, con el cual colaboró en el juicio contra su jefe.*
**21**	RES-QUE	*El equipo despidió al entrenador con el que perdieron el campeonato nacional.*
	RES-CUAL	*El equipo despidió al entrenador con el cual perdieron el campeonato nacional.*
	NON-QUE	*El equipo despidió a su entrenador, con el que perdieron el campeonato nacional.*
	NON-CUAL	*El equipo despidió a su entrenador, con el cual perdieron el campeonato nacional.*
**22**	RES-QUE	*El rey recibió al conde con el que establecería un acuerdo de gobierno.*
	RES-CUAL	*El rey recibió al conde con el cual establecería un acuerdo de gobierno.*
	NON-QUE	*El rey recibió a un conde, con el que establecería un acuerdo de gobierno.*
	NON-CUAL	*El rey recibió a un conde, con el cual establecería un acuerdo de gobierno.*
**23**	RES-QUE	*Manuel no conoce a la hija a la que abandonó de niña en un orfanato.*
	RES-CUAL	*Manuel no conoce a la hija a la cual abandonó de niña en un orfanato.*
	NON-QUE	*Manuel no conoce a su hija, a la que abandonó de niña en un orfanato.*
	NON-CUAL	*Manuel no conoce a su hija, a la cual abandonó de niña en un orfanato.*
**24**	RES-QUE	*Marta discutió con el vecino con el que compartía el patio de la casa.*
	RES-CUAL	*Marta discutió con el vecino con el cual compartía el patio de la casa.*
	NON-QUE	*Marta discutió con su vecino, con el que compartía el patio de la casa.*
	NON-CUAL	*Marta discutió con su vecino, con el cual compartía el patio de la casa.*
**25**	RES-QUE	*El presidente confiaba en el ministro al que encargó la redacción de la ley.*
	RES-CUAL	*El presidente confiaba en el ministro al cual encargó la redacción de la ley.*
	NON-QUE	*El presidente confiaba en su ministro, al que encargó la redacción de la ley.*
	NON-CUAL	*El presidente confiaba en su ministro, al cual encargó la redacción de la ley.*
**26**	RES-QUE	*Marisa visitó a la amiga a la que ayudó con la mudanza de los muebles.*
	RES-CUAL	*Marisa visitó a la amiga a la cual ayudó con la mudanza de los muebles.*
	NON-QUE	*Marisa visitó a su amiga, a la que ayudó con la mudanza de los muebles.*
	NON-CUAL	*Marisa visitó a su amiga, a la cual ayudó con la mudanza de los muebles.*
**27**	RES-QUE	*Francisco discutió con el hermano con el que trabaja en la oficina de correos.*
	RES-CUAL	*Francisco discutió con el hermano con el cual trabaja en la oficina de correos.*
	NON-QUE	*Francisco discutió con su hermano, con el que trabaja en la oficina de correos.*
	NON-CUAL	*Francisco discutió con su hermano, con el cual trabaja en la oficina de correos.*
**28**	RES-QUE	*El médico visitó al paciente al que trataba por sus problemas de insomnio.*

	RES-CUAL	*El médico visitó al paciente al cual trataba por sus problemas de insomnio.*
	NON-QUE	*El médico visitó a su paciente, al que trataba por sus problemas de insomnio.*
	NON-CUAL	*El médico visitó a su paciente, al cual trataba por sus problemas de insomnio.*
**29**	RES-QUE	*Fernando perdió al perro al que adoptó en una protectora de animales.*
	RES-CUAL	*Fernando perdió al perro al cual adoptó en una protectora de animales.*
	NON-QUE	*Fernando perdió a su perro, al que adoptó en una protectora de animales.*
	NON-CUAL	*Fernando perdió a su perro, al cual adoptó en una protectora de animales.*
**30**	RES-QUE	*Juan arregló el coche con el que reparte la comida del restaurante.*
	RES-CUAL	*Juan arregló el coche con el cual reparte la comida del restaurante.*
	NON-QUE	*Juan arregló su coche, con el que reparte la comida del restaurante.*
	NON-CUAL	*Juan arregló su coche, con el cual reparte la comida del restaurante.*
**31**	RES-QUE	*Miguel perdió el empleo por el que viajaba a menudo al extranjero.*
	RES-CUAL	*Miguel perdió el empleo por el cual viajaba a menudo al extranjero.*
	NON-QUE	*Miguel perdió su empleo, por el que viajaba a menudo al extranjero.*
	NON-CUAL	*Miguel perdió su empleo, por el cual viajaba a menudo al extranjero.*
**32**	RES-QUE	*El cantante perdió la guitarra con la que tocó en el concierto de anoche.*
	RES-CUAL	*El cantante perdió la guitarra con la cual tocó en el concierto de anoche.*
	NON-QUE	*El cantante perdió su guitarra, con la que tocó en el concierto de anoche.*
	NON-CUAL	*El cantante perdió su guitarra, con la cual tocó en el concierto de anoche.*
**33**	RES-QUE	*Francisco firmó el contrato por el que comenzaría a trabajar en el banco.*
	RES-CUAL	*Francisco firmó el contrato por el cual comenzaría a trabajar en el banco.*
	NON-QUE	*Francisco firmó su contrato, por el que comenzaría a trabajar en el banco.*
	NON-CUAL	*Francisco firmó su contrato, por el cual comenzaría a trabajar en el banco.*
**34**	RES-QUE	*El rey perdió la corona con la que desfilaría el día de su coronación.*
	RES-CUAL	*El rey perdió la corona con la cual desfilaría el día de su coronación.*
	NON-QUE	*El rey perdió su corona, con la que desfiló el día de su coronación.*
	NON-CUAL	*El rey perdió su corona, con la cual desfiló el día de su coronación.*
**35**	RES-QUE	*Mi primo rompió la copa con la que brindó en la fiesta de cumpleaños.*
	RES-CUAL	*Mi primo rompió la copa con la cual brindó en la fiesta de cumpleaños.*
	NON-QUE	*Mi primo rompió su copa, con la que brindó en la fiesta de cumpleaños.*
	NON-CUAL	*Mi primo rompió su copa, con la cual brindó en la fiesta de cumpleaños.*
**36**	RES-QUE	*Marta me devolvió el dinero con el que compró la televisión del salón.*
	RES-CUAL	*Marta me devolvió el dinero con el cual compró la televisión del salón.*
	NON-QUE	*Marta me devolvió mi dinero, con el que compró la televisión del salón.*
	NON-CUAL	*Marta me devolvió mi dinero, con el cual compró la televisión del salón.*
**37**	RES-QUE	*El profesor me dejó el libro con el que preparaba las clases de física.*
	RES-CUAL	*El profesor me dejó el libro con el cual preparaba las clases de física.*
	NON-QUE	*El profesor me dejó su libro, con el que preparaba las clases de física.*
	NON-CUAL	*El profesor me dejó su libro, con el cual preparaba las clases de física.*
**38**	RES-QUE	*El asesino ocultó el arma con la que cometió el crimen en el banco.*
	RES-CUAL	*El asesino ocultó el arma con la cual cometió el crimen en el banco.*
	NON-QUE	*El asesino ocultó su arma, con la que cometió el crimen en el banco.*
	NON-CUAL	*El asesino ocultó su arma, con la cual cometió el crimen en el banco.*
**39**	RES-QUE	*La universidad firmó el convenio con el que esperaba mejorar su educación.*
	RES-CUAL	*La universidad firmó el convenio con el cual esperaba mejorar su educación.*

	NON-QUE	*La universidad firmó un convenio, con el que esperaba mejorar su educación.*
	NON-CUAL	*La universidad firmó un convenio, con el cual esperaba mejorar su educación.*
**40**	RES-QUE	*El capitán reparó el barco con el que viajaría alrededor del mundo.*
	RES-CUAL	*El capitán reparó el barco con el cual viajaría alrededor del mundo.*
	NON-QUE	*El capitán reparó su barco, con el que viajaría alrededor del mundo.*
	NON-CUAL	*El capitán reparó su barco, con el cual viajaría alrededor del mundo.*
**41**	RES-QUE	*La enfermera perdió la agenda en la que anotaba las consultas del médico.*
	RES-CUAL	*La enfermera perdió la agenda en la cual anotaba las consultas del médico.*
	NON-QUE	*La enfermera perdió su agenda, en la que anotaba las consultas del médico.*
	NON-CUAL	*La enfermera perdió su agenda, en la cual anotaba las consultas del médico.*
**42**	RES-QUE	*El pintor inauguró la exposición en la que presentó su colección de arte.*
	RES-CUAL	*El pintor inauguró la exposición en la cual presentó su colección de arte.*
	NON-QUE	*El pintor inauguró su exposición, en la que presentó su colección de arte.*
	NON-CUAL	*El pintor inauguró su exposición, en la cual presentó su colección de arte.*
**43**	RES-QUE	*El paciente visitó al médico con el que realizaba las pruebas del corazón.*
	RES-CUAL	*El paciente visitó al médico con el cual realizaba las pruebas del corazón.*
	NON-QUE	*El paciente visitó a su médico, con el que realizaba las pruebas del corazón.*
	NON-CUAL	*El paciente visitó a su médico, con el cual realizaba las pruebas del corazón.*
**44**	RES-QUE	*La afición homenajeó al entrenador con el que ganó la liga de fútbol.*
	RES-CUAL	*La afición homenajeó al entrenador con el cual ganó la liga de fútbol.*
	NON-QUE	*La afición homenajeó a su entrenador, con el que ganó la liga de fútbol.*
	NON-CUAL	*La afición homenajeó a su entrenador, con el cual ganó la liga de fútbol.*
**45**	RES-QUE	*Elena discutió con la compañera con la que comparte el despacho de la facultad.*
	RES-CUAL	*Elena discutió con la compañera con la cual comparte el despacho de la facultad.*
	NON-QUE	*Elena discutió con su compañera, con la que comparte el despacho de la facultad.*
	NON-CUAL	*Elena discutió con su compañera, con la cual comparte el despacho de la facultad.*
**46**	RES-QUE	*El equipo derrotó al rival contra el que jugó el partido de la eliminatoria.*
	RES-CUAL	*El equipo derrotó al rival contra el cual jugó el partido de la eliminatoria.*
	NON-QUE	*El equipo derrotó a su rival, contra el que jugó el partido de la eliminatoria.*
	NON-CUAL	*El equipo derrotó a su rival, contra el cual jugó el partido de la eliminatoria.*
**47**	RES-QUE	*El detective felicitó al compañero con el que resolvió el robo de la joyería.*
	RES-CUAL	*El detective felicitó al compañero con el cual resolvió el robo de la joyería.*
	NON-QUE	*El detective felicitó a su compañero, con el que resolvió el robo de la joyería.*
	NON-CUAL	*El detective felicitó a su compañero, con el cual resolvió el robo de la joyería.*
**48**	RES-QUE	*Pablo visitó a la familia a la que conoció en su viaje a España.*
	RES-CUAL	*Pablo visitó a la familia a la cual conoció en su viaje a España.*
	NON-QUE	*Pablo visitó a mi familia, a la que conoció en su viaje a España.*
	NON-CUAL	*Pablo visitó a mi familia, a la cual conoció en su viaje a España.*
**49**	RES-QUE	*La orquesta contrató al director con el que trabajaría en la nueva ópera.*
	RES-CUAL	*La orquesta contrató al director con el cual trabajaría en la nueva ópera.*
	NON-QUE	*La orquesta contrató a un director, con el que trabajaría en la nueva ópera.*
	NON-CUAL	*La orquesta contrató a un director, con el cual trabajaría en la nueva ópera.*
**50**	RES-QUE	*María me presentó a la hermana con la que compartía su habitación de pequeña.*
	RES-CUAL	*María me presentó a la hermana con la cual compartía su habitación de pequeña.*
	NON-QUE	*María me presentó a su hermana, con la que compartía su habitación de pequeña.*

	NON-CUAL	*María me presentó a su hermana, con la cual compartía su habitación de pequeña.*
**51**	RES-QUE	*Patricia perdió al hijo con el que salió de excursión al campo.*
	RES-CUAL	*Patricia perdió al hijo con el cual salió de excursión al campo.*
	NON-QUE	*Patricia perdió a su hijo, con el que salió de excursión al campo.*
	NON-CUAL	*Patricia perdió a su hijo, con el cual salió de excursión al campo.*
**52**	RES-QUE	*El diseñador viajó con la modelo a la que contrató para el desfile de moda.*
	RES-CUAL	*El diseñador viajó con la modelo a la cual contrató para el desfile de moda.*
	NON-QUE	*El diseñador viajó con su modelo, a la que contrató para el desfile de moda.*
	NON-CUAL	*El diseñador viajó con su modelo, a la cual contrató para el desfile de moda.*
**53**	RES-QUE	*El ladrón se disculpó con la víctima a la que robó el colgante de su abuela.*
	RES-CUAL	*El ladrón se disculpó con la víctima a la cual robó el colgante de su abuela.*
	NON-QUE	*El ladrón se disculpó con su víctima, a la que robó el colgante de su abuela.*
	NON-CUAL	*El ladrón se disculpó con su víctima, a la cual robó el colgante de su abuela.*
**54**	RES-QUE	*El cantante visitó al compositor con el que compone muchas de sus canciones.*
	RES-CUAL	*El cantante visitó al compositor con el cual compone muchas de sus canciones.*
	NON-QUE	*El cantante visitó a su compositor, con el que compone muchas de sus canciones.*
	NON-CUAL	*El cantante visitó a su compositor, con el cual compone muchas de sus canciones.*
**55**	RES-QUE	*El actor recordó al maestro con el que realizó sus primeras interpretaciones.*
	RES-CUAL	*El actor recordó al maestro con el cual realizó sus primeras interpretaciones.*
	NON-QUE	*El actor recordó a su maestro, con el que realizó sus primeras interpretaciones.*
	NON-CUAL	*El actor recordó a su maestro, con el cual realizó sus primeras interpretaciones.*
**56**	RES-QUE	*El presidente destituyó al ministro con el que discutió en la rueda de prensa.*
	RES-CUAL	*El presidente destituyó al ministro con el cual discutió en la rueda de prensa.*
	NON-QUE	*El presidente destituyó a su ministro, con el que discutió en la rueda de prensa.*
	NON-CUAL	*El presidente destituyó a su ministro, con el cual discutió en la rueda de prensa.*

2. Experimental stimuli of the contrast *Que* vs *Quien*

**1**	RES-QUE	*La actriz se reconcilió con el novio al que abandonó tras el rodaje de la película.*
	RES-QUIEN	*La actriz se reconcilió con el novio a quien abandonó tras el rodaje de la película.*
	NON-QUE	*La actriz se reconcilió con su novio, al que abandonó tras el rodaje de la película.*
	NON-QUIEN	*La actriz se reconcilió con su novio, a quien abandonó tras el rodaje de la película.*
**2**	RES-QUE	*El gobierno destituyó al diputado al que acusaron de aliarse con la oposición.*
	RES-QUIEN	*El gobierno destituyó al diputado a quien acusaron de aliarse con la oposición.*
	NON-QUE	*El gobierno destituyó a su diputado, al que acusaron de aliarse con la oposición.*
	NON-QUIEN	*El gobierno destituyó a su diputado, a quien acusaron de aliarse con la oposición.*
**3**	RES-QUE	*El teatro presentó al artista al que contrató para el nuevo espectáculo.*
	RES-QUIEN	*El teatro presentó al artista a quien contrató para el nuevo espectáculo.*
	NON-QUE	*El teatro presentó a su artista, al que contrató para el nuevo espectáculo.*
	NON-QUIEN	*El teatro presentó a su artista, a quien contrató para el nuevo espectáculo.*
**4**	RES-QUE	*El sindicato presentará al presidente al que eligieron en la votación de ayer.*
	RES-QUIEN	*El sindicato presentará al presidente a quien eligieron en la votación de ayer.*
	NON-QUE	*El sindicato presentará a su presidente, al que eligieron en la votación de ayer.*
	NON-QUIEN	*El sindicato presentará a su presidente, a quien eligieron en la votación de ayer.*
**5**	RES-QUE	*Miguel me presentó al jefe al que acompañó en un viaje de negocios.*

	RES-QUIEN	*Miguel me presentó al jefe a quien acompañó en un viaje de negocios.*
	NON-QUE	*Miguel me presentó a su jefe, al que acompañó en un viaje de negocios.*
	NON-QUIEN	*Miguel me presentó a su jefe, a quien acompañó en un viaje de negocios.*
**6**	RES-QUE	*El director felicitó al trabajador al que concedieron un premio por su trabajo.*
	RES-QUIEN	*El director felicitó al trabajador a quien concedieron un premio por su trabajo.*
	NON-QUE	*El director felicitó a su trabajador, al que concedieron un premio por su trabajo.*
	NON-QUIEN	*El director felicitó a su trabajador, a quien concedieron un premio por su trabajo.*
**7**	RES-QUE	*Mi amiga vive con el hermano al que acompañaba al colegio de pequeña.*
	RES-QUIEN	*Mi amiga vive con el hermano a quien acompañaba al colegio de pequeña.*
	NON-QUE	*Mi amiga vive con su hermano, al que acompañaba al colegio de pequeña.*
	NON-QUIEN	*Mi amiga vive con su hermano, a quien acompañaba al colegio de pequeña.*
**8**	RES-QUE	*El granjero llamó al veterinario del que dependía el cuidado de los animales.*
	RES-QUIEN	*El granjero llamó al veterinario de quien dependía el cuidado de los animales.*
	NON-QUE	*El granjero llamó a su veterinario, del que dependía el cuidado de los animales.*
	NON-QUIEN	*El granjero llamó a su veterinario, de quien dependía el cuidado de los animales.*
**9**	RES-QUE	*El entrenador se enfadó con el jugador al que fichó al final de la temporada.*
	RES-QUIEN	*El entrenador se enfadó con el jugador a quien fichó al final de la temporada.*
	NON-QUE	*El entrenador se enfadó con su jugador, al que fichó al final de la temporada.*
	NON-QUIEN	*El entrenador se enfadó con su jugador, a quien fichó al final de la temporada.*
**10**	RES-QUE	*El taxista no encontraba al pasajero al que esperaba en el arcén de la carretera.*
	RES-QUIEN	*El taxista no encontraba al pasajero a quien esperaba en el arcén de la carretera.*
	NON-QUE	*El taxista no encontraba a su pasajero, al que esperaba en el arcén de la carretera.*
	NON-QUIEN	*El taxista no encontraba a su pasajero, a quien esperaba en el arcén de la carretera.*
**11**	RES-QUE	*Los ciudadanos criticaron al alcalde al que votaron en las últimas elecciones.*
	RES-QUIEN	*Los ciudadanos criticaron al alcalde a quien votaron en las últimas elecciones.*
	NON-QUE	*Los ciudadanos criticaron a su alcalde, al que votaron en las últimas elecciones.*
	NON-QUIEN	*Los ciudadanos criticaron a su alcalde, a quien votaron en las últimas elecciones.*
**12**	RES-QUE	*Mi amiga se enamoró del médico al que conoció en mi fiesta de navidad.*
	RES-QUIEN	*Mi amiga se enamoró del médico a quien conoció en mi fiesta de navidad.*
	NON-QUE	*Mi amiga se enamoró de mi médico, al que conoció en mi fiesta de navidad.*
	NON-QUIEN	*Mi amiga se enamoró de mi médico, a quien conoció en mi fiesta de navidad.*
**13**	RES-QUE	*La revista despidió al periodista al que descubrieron mintiendo en sus artículos.*
	RES-QUIEN	*La revista despidió al periodista a quien descubrieron mintiendo en sus artículos.*
	NON-QUE	*La revista despidió a un periodista, al que descubrieron mintiendo en sus artículos.*
	NON-QUIEN	*La revista despidió a un periodista, a quien descubrieron mintiendo en sus artículos.*
**14**	RES-QUE	*Clara visitó al amigo al que conoció de joven en Londres.*
	RES-QUIEN	*Clara visitó al amigo a quien conoció de joven en Londres.*
	NON-QUE	*Clara visitó a su amigo, al que conoció de joven en Londres.*
	NON-QUIEN	*Clara visitó a su amigo, a quien conoció de joven en Londres.*
**15**	RES-QUE	*El actor trabajará con el director al que admira desde hace varios años.*
	RES-QUIEN	*El actor trabajará con el director a quien admira desde hace varios años.*
	NON-QUE	*El actor trabajará con su director, al que admira desde hace varios años.*
	NON-QUIEN	*El actor trabajará con su director, a quien admira desde hace varios años.*
**16**	RES-QUE	*La modelo discutió con el asesor al que contrató para la campaña publicitaria.*
	RES-QUIEN	*La modelo discutió con el asesor a quien contrató para la campaña publicitaria.*

	NON-QUE	*La modelo discutió con su asesor, al que contrató para la campaña publicitaria.*
	NON-QUIEN	*La modelo discutió con su asesor, a quien contrató para la campaña publicitaria.*
**17**	RES-QUE	*El presentador criticó al rey al que entrevistó para el reportaje de este mes.*
	RES-QUIEN	*El presentador criticó al rey a quien entrevistó para el reportaje de este mes.*
	NON-QUE	*El presentador criticó al rey, al que entrevistó para el reportaje de este mes.*
	NON-QUIEN	*El presentador criticó al rey, a quien entrevistó para el reportaje de este mes.*
**18**	RES-QUE	*El banco contactó con el cliente al que ayudaba a gestionar su patrimonio.*
	RES-QUIEN	*El banco contactó con el cliente a quien ayudaba a gestionar su patrimonio.*
	NON-QUE	*El banco contactó con su cliente, al que ayudaba a gestionar su patrimonio.*
	NON-QUIEN	*El banco contactó con su cliente, a quien ayudaba a gestionar su patrimonio.*
**19**	RES-QUE	*Mi primo me presentó al amigo al que ayuda a estudiar para los exámenes.*
	RES-QUIEN	*Mi primo me presentó al amigo a quien ayuda a estudiar para los exámenes.*
	NON-QUE	*Mi primo me presentó a su amigo, al que ayuda a estudiar para los exámenes.*
	NON-QUIEN	*Mi primo me presentó a su amigo, a quien ayuda a estudiar para los exámenes.*
**20**	RES-QUE	*El profesor se reunió con el estudiante al que aconsejaría en su trabajo final.*
	RES-QUIEN	*El profesor se reunió con el estudiante a quien aconsejaría en su trabajo final.*
	NON-QUE	*El profesor se reunió con un estudiante, al que aconsejaría en su trabajo final.*
	NON-QUIEN	*El profesor se reunió con un estudiante, a quien aconsejaría en su trabajo final.*
**21**	RES-QUE	*El museo despidió al pintor al que encargaba la reparación de los cuadros.*
	RES-QUIEN	*El museo despidió al pintor a quien encargaba la reparación de los cuadros.*
	NON-QUE	*El museo despidió a un pintor, al que encargaba la reparación de los cuadros.*
	NON-QUIEN	*El museo despidió a un pintor, a quien encargaba la reparación de los cuadros.*
**22**	RES-QUE	*El decano despidió al secretario del que dependía la organización de las clases.*
	RES-QUIEN	*El decano despidió al secretario de quien dependía la organización de las clases.*
	NON-QUE	*El decano despidió a su secretario, del que dependía la organización de las clases.*
	NON-QUIEN	*El decano despidió a su secretario, de quien dependía la organización de las clases.*
**23**	RES-QUE	*Mi hermana cenó con un amigo al que llevaba mucho tiempo sin ver.*
	RES-QUIEN	*Mi hermana cenó con un amigo a quien llevaba mucho tiempo sin ver.*
	NON-QUE	*Mi hermana cenó con su amigo, al que llevaba mucho tiempo sin ver.*
	NON-QUIEN	*Mi hermana cenó con su amigo, a quien llevaba mucho tiempo sin ver.*
**24**	RES-QUE	*Los soldados homenajearon al coronel al que debían el éxito de la batalla.*
	RES-QUIEN	*Los soldados homenajearon al coronel a quien debían el éxito de la batalla.*
	NON-QUE	*Los soldados homenajearon a su coronel, al que debían el éxito de la batalla.*
	NON-QUIEN	*Los soldados homenajearon a su coronel, a quien debían el éxito de la batalla.*
**25**	RES-QUE	*El cocinero contrató al ayudante al que encargaría preparar los postres.*
	RES-QUIEN	*El cocinero contrató al ayudante a quien encargaría preparar los postres.*
	NON-QUE	*El cocinero contrató a su ayudante, al que encargaría preparar los postres.*
	NON-QUIEN	*El cocinero contrató a su ayudante, a quien encargaría preparar los postres.*
**26**	RES-QUE	*Los diputados apoyaron al líder al que eligieron para representar al partido.*
	RES-QUIEN	*Los diputados apoyaron al líder a quien eligieron para representar al partido.*
	NON-QUE	*Los diputados apoyaron a su líder, al que eligieron para representar al partido.*
	NON-QUIEN	*Los diputados apoyaron a su líder, a quien eligieron para representar al partido.*
**27**	RES-QUE	*El senado elegirá al portavoz al que encargarán los comunicados de prensa.*
	RES-QUIEN	*El senado elegirá al portavoz a quien encargarán los comunicados de prensa.*
	NON-QUE	*El senado elegirá a su portavoz, al que encargarán los comunicados de prensa.*

	NON-QUIEN	*El senado elegirá a su portavoz, a quien encargarán los comunicados de prensa.*
**28**	RES-QUE	*Vicente me presentó al camarero al que contrató para el nuevo restaurante.*
	RES-QUIEN	*Vicente me presentó al camarero a quien contrató para el nuevo restaurante.*
	NON-QUE	*Vicente me presentó a su camarero, al que contrató para el nuevo restaurante.*
	NON-QUIEN	*Vicente me presentó a su camarero, a quien contrató para el nuevo restaurante.*
**29**	RES-QUE	*La policía interrogó al vecino al que encontró en la escena del crimen.*
	RES-QUIEN	*La policía interrogó al vecino a quien encontró en la escena del crimen.*
	NON-QUE	*La policía interrogó a mi vecino, al que encontró en la escena del crimen.*
	NON-QUIEN	*La policía interrogó a mi vecino, a quien encontró en la escena del crimen.*
**30**	RES-QUE	*El duque confiaba en el rey al que ayudaba en la administración del reino.*
	RES-QUIEN	*El duque confiaba en el rey a quien ayudaba en la administración del reino.*
	NON-QUE	*El duque confiaba en su rey, al que ayudaba en la administración del reino.*
	NON-QUIEN	*El duque confiaba en su rey, a quien ayudaba en la administración del reino.*
**31**	RES-QUE	*Rebeca añoraba al amigo al que conoció en su viaje por África.*
	RES-QUIEN	*Rebeca añoraba al amigo a quien conoció en su viaje por África.*
	NON-QUE	*Rebeca añoraba a su amigo, al que conoció en su viaje por África.*
	NON-QUIEN	*Rebeca añoraba a su amigo, a quien conoció en su viaje por África.*
**32**	RES-QUE	*El acusado se alió con el abogado al que contrató para defenderlo en el juicio.*
	RES-QUIEN	*El acusado se alió con el abogado a quien contrató para defenderlo en el juicio.*
	NON-QUE	*El acusado se alió con su abogado, al que contrató para defenderlo en el juicio.*
	NON-QUIEN	*El acusado se alió con su abogado, a quien contrató para defenderlo en el juicio.*
**33**	RES-QUE	*La facultad recibió al decano al que votaron en la reunión del jueves.*
	RES-QUIEN	*La facultad recibió al decano a quien votaron en la reunión del jueves.*
	NON-QUE	*La facultad recibió a su decano, al que votaron en la reunión del jueves.*
	NON-QUIEN	*La facultad recibió a su decano, a quien votaron en la reunión del jueves.*
**34**	RES-QUE	*Rodrigo llamó al dentista al que acudía desde hacía varios años.*
	RES-QUIEN	*Rodrigo llamó al dentista a quien acudía desde hacía varios años.*
	NON-QUE	*Rodrigo llamó a su dentista, al que acudía desde hacía varios años.*
	NON-QUIEN	*Rodrigo llamó a su dentista, a quien acudía desde hacía varios años.*
**35**	RES-QUE	*El pasajero esperaba al taxista al que llamó desde la recepción del hotel.*
	RES-QUIEN	*El pasajero esperaba al taxista a quien llamó desde la recepción del hotel.*
	NON-QUE	*El pasajero esperaba a su taxista, al que llamó desde la recepción del hotel.*
	NON-QUIEN	*El pasajero esperaba a su taxista, a quien llamó desde la recepción del hotel.*
**36**	RES-QUE	*Jorge se despidió del amigo al que acompañó a la estación del tren.*
	RES-QUIEN	*Jorge se despidió del amigo a quien acompañó a la estación del tren.*
	NON-QUE	*Jorge se despidió de su amigo, al que acompañó a la estación del tren.*
	NON-QUIEN	*Jorge se despidió de su amigo, a quien acompañó a la estación del tren.*
**37**	RES-QUE	*El psicólogo visitó al paciente al que ayudaba con sus problemas de memoria.*
	RES-QUIEN	*El psicólogo visitó al paciente a quien ayudaba con sus problemas de memoria.*
	NON-QUE	*El psicólogo visitó a su paciente, al que ayudaba con sus problemas de memoria.*
	NON-QUIEN	*El psicólogo visitó a su paciente, a quien ayudaba con sus problemas de memoria.*
**38**	RES-QUE	*Mi hermana discutió con el maestro al que veía en las clases de matemáticas.*
	RES-QUIEN	*Mi hermana discutió con el maestro a quien veía en las clases de matemáticas.*
	NON-QUE	*Mi hermana discutió con su maestro, al que veía en las clases de matemáticas.*
	NON-QUIEN	*Mi hermana discutió con su maestro, a quien veía en las clases de matemáticas.*

**39**	RES-QUE	*Paula se reunió con el asesor al que contrató para administrar su herencia.*
	RES-QUIEN	*Paula se reunió con el asesor a quien contrató para administrar su herencia.*
	NON-QUE	*Paula se reunió con su asesor, al que contrató para administrar su herencia.*
	NON-QUIEN	*Paula se reunió con su asesor, a quien contrató para administrar su herencia.*
**40**	RES-QUE	*Carmen se mudó con el hijo al que adoptó de pequeño en un orfanato.*
	RES-QUIEN	*Carmen se mudó con el hijo a quien adoptó de pequeño en un orfanato.*
	NON-QUE	*Carmen se mudó con su hijo, al que adoptó de pequeño en un orfanato.*
	NON-QUIEN	*Carmen se mudó con su hijo, a quien adoptó de pequeño en un orfanato.*
**41**	RES-QUE	*El entrenador premió al atleta al que ayudaba para ganar la competición.*
	RES-QUIEN	*El entrenador premió al atleta a quien ayudaba para ganar la competición.*
	NON-QUE	*El entrenador premió a su atleta, al que ayudaba para ganar la competición.*
	NON-QUIEN	*El entrenador premió a su atleta, a quien ayudaba para ganar la competición.*
**42**	RES-QUE	*La cafetería despidió al camarero al que contrató para la temporada de verano.*
	RES-QUIEN	*La cafetería despidió al camarero a quien contrató para la temporada de verano.*
	NON-QUE	*La cafetería despidió a su camarero, al que contrató para la temporada de verano.*
	NON-QUIEN	*La cafetería despidió a su camarero, a quien contrató para la temporada de verano.*
**43**	RES-QUE	*La actriz se comprometió con el novio al que conoció en la nueva película.*
	RES-QUIEN	*La actriz se comprometió con el novio a quien conoció en la nueva película.*
	NON-QUE	*La actriz se comprometió con su novio, al que conoció en la nueva película.*
	NON-QUIEN	*La actriz se comprometió con su novio, a quien conoció en la nueva película.*
**44**	RES-QUE	*El estudiante discutió con el profesor al que criticó por poner notas bajas.*
	RES-QUIEN	*El estudiante discutió con el profesor a quien criticó por poner notas bajas.*
	NON-QUE	*El estudiante discutió con su profesor, al que criticó por poner notas bajas.*
	NON-QUIEN	*El estudiante discutió con su profesor, a quien criticó por poner notas bajas.*
**45**	RES-QUE	*El árbitro discutió con el jugador al que expulsó tras meter el gol.*
	RES-QUIEN	*El árbitro discutió con el jugador a quien expulsó tras meter el gol.*
	NON-QUE	*El árbitro discutió con un jugador, al que expulsó tras meter el gol.*
	NON-QUIEN	*El árbitro discutió con un jugador, a quien expulsó tras meter el gol.*
**46**	RES-QUE	*La editorial presentó al escritor al que ayudaron a publicar su novela.*
	RES-QUIEN	*La editorial presentó al escritor a quien ayudaron a publicar su novela.*
	NON-QUE	*La editorial presentó a su escritor, al que ayudaron a publicar su novela.*
	NON-QUIEN	*La editorial presentó a su escritor, a quien ayudaron a publicar su novela.*
**47**	RES-QUE	*Jesús cenó con el jefe al que contrataron antes de las vacaciones.*
	RES-QUIEN	*Jesús cenó con el jefe a quien contrataron antes de las vacaciones.*
	NON-QUE	*Jesús cenó con su jefe, al que contrataron antes de las vacaciones.*
	NON-QUIEN	*Jesús cenó con su jefe, a quien contrataron antes de las vacaciones.*
**48**	RES-QUE	*La radio felicitó al periodista al que concedieron el premio por su trabajo.*
	RES-QUIEN	*La radio felicitó al periodista a quien concedieron el premio por su trabajo.*
	NON-QUE	*La radio felicitó a su periodista, al que concedieron el premio por su trabajo.*
	NON-QUIEN	*La radio felicitó a su periodista, a quien concedieron el premio por su trabajo.*
**49**	RES-QUE	*El ejército homenajeó al soldado al que perdieron en la última batalla.*
	RES-QUIEN	*El ejército homenajeó al soldado a quien perdieron en la última batalla.*
	NON-QUE	*El ejército homenajeó a un soldado, al que perdieron en la última batalla.*
	NON-QUIEN	*El ejército homenajeó a un soldado, a quien perdieron en la última batalla.*
**50**	RES-QUE	*La empresa despidió al trabajador al que culpaban del robo del dinero.*

	RES-QUIEN	*La empresa despidió al trabajador a quien culpaban del robo del dinero.*
	NON-QUE	*La empresa despidió a su trabajador, al que culpaban del robo del dinero.*
	NON-QUIEN	*La empresa despidió a su trabajador, a quien culpaban del robo del dinero.*
**51**	RES-QUE	*La afición aclamó al jugador al que dedicaron una calle de la ciudad.*
	RES-QUIEN	*La afición aclamó al jugador a quien dedicaron una calle de la ciudad.*
	NON-QUE	*La afición aclamó a su jugador, al que dedicaron una calle de la ciudad.*
	NON-QUIEN	*La afición aclamó a su jugador, a quien dedicaron una calle de la ciudad.*
**52**	RES-QUE	*El abogado visitó al cliente al que defenderá en el juicio de mañana.*
	RES-QUIEN	*El abogado visitó al cliente a quien defenderá en el juicio de mañana.*
	NON-QUE	*El abogado visitó a su cliente, al que defenderá en el juicio de mañana.*
	NON-QUIEN	*El abogado visitó a su cliente, a quien defenderá en el juicio de mañana.*
**53**	RES-QUE	*Mateo se reunió con el compañero al que ayudaba a estudiar para el examen.*
	RES-QUIEN	*Mateo se reunió con el compañero a quien ayudaba a estudiar para el examen.*
	NON-QUE	*Mateo se reunió con su compañero, al que ayudaba a estudiar para el examen.*
	NON-QUIEN	*Mateo se reunió con su compañero, a quien ayudaba a estudiar para el examen.*
**54**	RES-QUE	*Guillermo encubrió al amigo al que inculparon del robo en el banco.*
	RES-QUIEN	*Guillermo encubrió al amigo a quien inculparon del robo en el banco.*
	NON-QUE	*Guillermo encubrió a su amigo, al que inculparon del robo en el banco.*
	NON-QUIEN	*Guillermo encubrió a su amigo, a quien inculparon del robo en el banco.*
**55**	RES-QUE	*El gobierno destituyó al presidente al que acusó de mentir en las elecciones.*
	RES-QUIEN	*El gobierno destituyó al presidente a quien acusó de mentir en las elecciones.*
	NON-QUE	*El gobierno destituyó a su presidente, al que acusó de mentir en las elecciones.*
	NON-QUIEN	*El gobierno destituyó a su presidente, a quien acusó de mentir en las elecciones.*
**56**	RES-QUE	*El departamento contrató al profesor al que encargaría organizar los cursos.*
	RES-QUIEN	*El departamento contrató al profesor a quien encargaría organizar los cursos.*
	NON-QUE	*El departamento contrató a un profesor, al que encargaría organizar los cursos.*
	NON-QUIEN	*El departamento contrató a un profesor, a quien encargaría organizar los cursos.*

3. Experimental stimuli of the contrast *Que* vs *Donde*

**1**	RES-QUE	*Mis amigos fueron al festival en el que tocaban sus cantantes favoritos.*
	RES-DONDE	*Mis amigos fueron al festival donde tocaban sus cantantes favoritos.*
	NON-QUE	*Mis amigos fueron a un festival, en el que tocaban sus cantantes favoritos.*
	NON-DONDE	*Mis amigos fueron a un festival, donde tocaban sus cantantes favoritos.*
**2**	RES-QUE	*Juan se mudó al país del que procedía su madre biológica.*
	RES-DONDE	*Juan se mudó al país de donde procedía su madre biológica.*
	NON-QUE	*Juan se mudó a su país, del que procedía su madre biológica.*
	NON-DONDE	*Juan se mudó a su país, de donde procedía su madre biológica.*
**3**	RES-QUE	*La policía registró el barrio del que escaparon unos criminales anoche.*
	RES-DONDE	*La policía registró el barrio de donde escaparon unos criminales anoche.*
	NON-QUE	*La policía registró mi barrio, del que escaparon unos criminales anoche.*
	NON-DONDE	*La policía registró mi barrio, de donde escaparon unos criminales anoche.*
**4**	RES-QUE	*La facultad cerró la biblioteca en la que estudiaban los alumnos del máster.*
	RES-DONDE	*La facultad cerró la biblioteca donde estudiaban los alumnos del máster.*
	NON-QUE	*La facultad cerró su biblioteca, en la que estudiaban los alumnos del máster.*

	NON-DONDE	*La facultad cerró su biblioteca, donde estudiaban los alumnos del máster.*
**5**	RES-QUE	*El pueblo podó el bosque del que emergían las plagas de insectos.*
	RES-DONDE	*El pueblo podó el bosque de donde emergían las plagas de insectos.*
	NON-QUE	*El pueblo podó su bosque, del que emergían las plagas de insectos.*
	NON-DONDE	*El pueblo podó su bosque, de donde emergían las plagas de insectos.*
**6**	RES-QUE	*El equipo reformó el estadio en el que hubo una avalancha el año pasado.*
	RES-DONDE	*El equipo reformó el estadio donde hubo una avalancha el año pasado.*
	NON-QUE	*El equipo reformó su estadio, en el que hubo una avalancha el año pasado.*
	NON-DONDE	*El equipo reformó su estadio, donde hubo una avalancha el año pasado.*
**7**	RES-QUE	*Elena dirige la empresa en la que empezó mi hermana a trabajar.*
	RES-DONDE	*Elena dirige la empresa donde empezó mi hermana a trabajar.*
	NON-QUE	*Elena dirige su empresa, en la que empezó mi hermana a trabajar.*
	NON-DONDE	*Elena dirige su empresa, donde empezó mi hermana a trabajar.*
**8**	RES-QUE	*Mis amigos abrieron el restaurante en el que celebraron mis padres una cena.*
	RES-DONDE	*Mis amigos abrieron el restaurante donde celebraron mis padres una cena.*
	NON-QUE	*Mis amigos abrieron un restaurante, en el que celebraron mis padres una cena.*
	NON-DONDE	*Mis amigos abrieron un restaurante, donde celebraron mis padres una cena.*
**9**	RES-QUE	*La ciudad inauguró el puerto del que partirían los barcos de mercancías.*
	RES-DONDE	*La ciudad inauguró el puerto de donde partirían los barcos de mercancías.*
	NON-QUE	*La ciudad inauguró su puerto, del que partirían los barcos de mercancías.*
	NON-DONDE	*La ciudad inauguró su puerto, de donde partirían los barcos de mercancías.*
**10**	RES-QUE	*La universidad valló el terreno al que acudían los jóvenes de fiesta.*
	RES-DONDE	*La universidad valló el terreno donde acudían los jóvenes de fiesta.*
	NON-QUE	*La universidad valló su terreno, al que acudían los jóvenes de fiesta.*
	NON-DONDE	*La universidad valló su terreno, donde acudían los jóvenes de fiesta.*
**11**	RES-QUE	*El príncipe regresó al imperio del que huyeron sus padres tras la rebelión.*
	RES-DONDE	*El príncipe regresó al imperio de donde huyeron sus padres tras la rebelión.*
	NON-QUE	*El príncipe regresó a su imperio, del que huyeron sus padres tras la rebelión.*
	NON-DONDE	*El príncipe regresó a su imperio, de donde huyeron sus padres tras la rebelión.*
**12**	RES-QUE	*El director premió al colegio en el que estudiaban los alumnos más brillantes.*
	RES-DONDE	*El director premió al colegio donde estudiaban los alumnos más brillantes.*
	NON-QUE	*El director premió a su colegio, en el que estudiaban los alumnos más brillantes.*
	NON-DONDE	*El director premió a su colegio, donde estudiaban los alumnos más brillantes.*
**13**	RES-QUE	*Mis padres visitaron la exposición en la que participan artistas extranjeros.*
	RES-DONDE	*Mis padres visitaron la exposición donde participan artistas extranjeros.*
	NON-QUE	*Mis padres visitaron una exposición, en la que participan artistas extranjeros.*
	NON-DONDE	*Mis padres visitaron una exposición, donde participan artistas extranjeros.*
**14**	RES-QUE	*Fernando visitó el pueblo en el que crecieron sus abuelos durante la guerra.*
	RES-DONDE	*Fernando visitó el pueblo donde crecieron sus abuelos durante la guerra.*
	NON-QUE	*Fernando visitó su pueblo, en el que crecieron sus abuelos durante la guerra.*
	NON-DONDE	*Fernando visitó su pueblo, donde crecieron sus abuelos durante la guerra.*
**15**	RES-QUE	*El granjero cultivó el campo en el que había abundante agua para regar.*
	RES-DONDE	*El granjero cultivó el campo donde había abundante agua para regar.*
	NON-QUE	*El granjero cultivó su campo, en el que había abundante agua para regar.*
	NON-DONDE	*El granjero cultivó su campo, donde había abundante agua para regar.*

**16**	RES-QUE	*El casero limpió el apartamento al que llegarían los nuevos inquilinos.*
	RES-DONDE	*El casero limpió el apartamento donde llegarían los nuevos inquilinos.*
	NON-QUE	*El casero limpió su apartamento, al que llegarían los nuevos inquilinos.*
	NON-DONDE	*El casero limpió su apartamento, donde llegarían los nuevos inquilinos.*
**17**	RES-QUE	*La escuela reformó el patio al que salían los niños en el recreo.*
	RES-DONDE	*La escuela reformó el patio donde salían los niños en el recreo.*
	NON-QUE	*La escuela reformó su patio, al que salían los niños en el recreo.*
	NON-DONDE	*La escuela reformó su patio, donde salían los niños en el recreo.*
**18**	RES-QUE	*El médico trabajaba en el hospital en el que estaban los enfermos más graves.*
	RES-DONDE	*El médico trabajaba en el hospital donde estaban los enfermos más graves.*
	NON-QUE	*El médico trabajaba en un hospital, en el que estaban los enfermos más graves.*
	NON-DONDE	*El médico trabajaba en un hospital, donde estaban los enfermos más graves.*
**19**	RES-QUE	*El fontanero visitó el edificio del que provenía la fuga de agua.*
	RES-DONDE	*El fontanero visitó el edificio de donde provenía la fuga de agua.*
	NON-QUE	*El fontanero visitó mi edificio, del que provenía una fuga de agua.*
	NON-DONDE	*El fontanero visitó mi edificio, de donde provenía una fuga de agua.*
**20**	RES-QUE	*El alcalde cerró el parque en el que jugaban los niños por las tardes.*
	RES-DONDE	*El alcalde cerró el parque donde jugaban los niños por las tardes.*
	NON-QUE	*El alcalde cerró un parque, en el que jugaban los niños por las tardes.*
	NON-DONDE	*El alcalde cerró el parque, donde jugaban los niños por las tardes.*
**21**	RES-QUE	*Mis abuelos vivían en el barrio al que iban los universitarios de fiesta.*
	RES-DONDE	*Mis abuelos vivían en el barrio donde iban los universitarios de fiesta.*
	NON-QUE	*Mis abuelos vivían en su barrio, al que iban los universitarios de fiesta.*
	NON-DONDE	*Mis abuelos vivían en su barrio, donde iban los universitarios de fiesta.*
**22**	RES-QUE	*El teatro reparó el escenario en el que ensayaban los actores los domingos.*
	RES-DONDE	*El teatro reparó el escenario donde ensayaban los actores los domingos.*
	NON-QUE	*El teatro reparó su escenario, en el que ensayaban los actores los domingos.*
	NON-DONDE	*El teatro reparó su escenario, donde ensayaban los actores los domingos.*
**23**	RES-QUE	*El pueblo celebrará el mercado al que asisten los comerciantes de la región.*
	RES-DONDE	*El pueblo celebrará el mercado donde asisten los comerciantes de la región.*
	NON-QUE	*El pueblo celebrará un mercado, al que asistirán los comerciantes de la región.*
	NON-DONDE	*El pueblo celebrará un mercado, donde asistirán los comerciantes de la región.*
**24**	RES-QUE	*La policía registró el pueblo del que procedía el hombre asesinado.*
	RES-DONDE	*La policía registró el pueblo de donde procedía el hombre asesinado.*
	NON-QUE	*La policía registró mi pueblo, del que procedía el hombre asesinado.*
	NON-DONDE	*La policía registró mi pueblo, de donde procedía el hombre asesinado.*
**25**	RES-QUE	*Mi hermana decoró el piso al que fueron sus amigos a vivir.*
	RES-DONDE	*Mi hermana decoró el piso donde fueron sus amigos a vivir.*
	NON-QUE	*Mi hermana decoró su piso, al que fueron sus amigos a vivir.*
	NON-DONDE	*Mi hermana decoró su piso, donde fueron sus amigos a vivir.*
**26**	RES-QUE	*El incendio arrasó el monte en el que paseaban los pastores con sus ovejas.*
	RES-DONDE	*El incendio arrasó el monte donde paseaban los pastores con sus ovejas.*
	NON-QUE	*El incendio arrasó el monte, en el que paseaban los pastores con sus ovejas.*
	NON-DONDE	*El incendio arrasó el monte, donde paseaban los pastores con sus ovejas.*
**27**	RES-QUE	*El ayuntamiento arreglará la carretera en la que sufrió mi vecino un accidente.*

	RES-DONDE	*El ayuntamiento arreglará la carretera donde sufrió mi vecino un accidente.*
	NON-QUE	*El ayuntamiento arreglará mi carretera, en la que sufrió mi vecino un accidente.*
	NON-DONDE	*El ayuntamiento arreglará mi carretera, donde sufrió mi vecino un accidente.*
**28**	RES-QUE	*El decano organizó el congreso en el que participaron investigadores famosos.*
	RES-DONDE	*El decano organizó el congreso donde participaron investigadores famosos.*
	NON-QUE	*El decano organizó un congreso, en el que participaron investigadores famosos.*
	NON-DONDE	*El decano organizó un congreso, donde participaron investigadores famosos.*
**29**	RES-QUE	*La policía cortó la calle por la que atravesaría el desfile de carnaval.*
	RES-DONDE	*La policía cortó la calle por donde atravesaría el desfile de carnaval.*
	NON-QUE	*La policía cortó una calle, por la que atravesaría el desfile de carnaval.*
	NON-DONDE	*La policía cortó una calle, por donde atravesaría el desfile de carnaval.*
**30**	RES-QUE	*La ciudad inaugurará el museo al que asistirán los pintores más conocidos.*
	RES-DONDE	*La ciudad inaugurará el museo donde asistirán los pintores más conocidos.*
	NON-QUE	*La ciudad inaugurará un museo, al que asistirán los pintores más conocidos.*
	NON-DONDE	*La ciudad inaugurará un museo, donde asistirán los pintores más conocidos.*
**31**	RES-QUE	*El arquitecto inspeccionó el piso del que procedía la avería en la electricidad.*
	RES-DONDE	*El arquitecto inspeccionó el piso de donde procedía la avería en la electricidad.*
	NON-QUE	*El arquitecto inspeccionó mi piso, del que procedía la avería en la electricidad.*
	NON-DONDE	*El arquitecto inspeccionó mi piso, de donde procedía la avería en la electricidad.*
**32**	RES-QUE	*El alcalde cerró el instituto en el que estudiaban pocos alumnos de bachiller.*
	RES-DONDE	*El alcalde cerró el instituto donde estudiaban pocos alumnos de bachiller.*
	NON-QUE	*El alcalde cerró el instituto, en el que estudiaban pocos alumnos de bachiller.*
	NON-DONDE	*El alcalde cerró el instituto, donde estudiaban pocos alumnos de bachiller.*
**33**	RES-QUE	*El jardinero arregló el jardín en el que celebraríamos la fiesta de cumpleaños.*
	RES-DONDE	*El jardinero arregló el jardín donde celebraríamos la fiesta de cumpleaños.*
	NON-QUE	*El jardinero arregló mi jardín, en el que celebraríamos la fiesta de cumpleaños.*
	NON-DONDE	*El jardinero arregló mi jardín, donde celebraríamos la fiesta de cumpleaños.*
**34**	RES-QUE	*La biblioteca cerró la sala en la que estaban los estudiantes hablando.*
	RES-DONDE	*La biblioteca cerró la sala donde estaban los estudiantes hablando.*
	NON-QUE	*La biblioteca cerró una sala, en la que estaban los estudiantes hablando.*
	NON-DONDE	*La biblioteca cerró una sala, donde estaban los estudiantes hablando.*
**35**	RES-QUE	*Laura visitó la prisión en la que estaba su hermano encarcelado.*
	RES-DONDE	*Laura visitó la prisión donde estaba su hermano encarcelado.*
	NON-QUE	*Laura visitó la prisión, en la que estaba su hermano encarcelado.*
	NON-DONDE	*Laura visitó la prisión, donde estaba su hermano encarcelado.*
**36**	RES-QUE	*La banda canceló el concierto al que asistirían sus seguidores más fieles.*
	RES-DONDE	*La banda canceló el concierto donde asistirían sus seguidores más fieles.*
	NON-QUE	*La banda canceló su concierto, al que asistirían sus seguidores más fieles.*
	NON-DONDE	*La banda canceló su concierto, donde asistirían sus seguidores más fieles.*
**37**	RES-QUE	*Cristian limpió el piso al que trasladarían sus padres los muebles.*
	RES-DONDE	*Cristian limpió el piso donde trasladarían sus padres los muebles.*
	NON-QUE	*Cristian limpió su piso, al que trasladarían sus padres los muebles.*
	NON-DONDE	*Cristian limpió su piso, donde trasladarían sus padres los muebles.*
**38**	RES-QUE	*La policía registró el barrio del que proceden los muchachos desaparecidos.*
	RES-DONDE	*La policía registró el barrio de donde proceden los muchachos desaparecidos.*

	NON-QUE	*La policía registró mi barrio, del que proceden los muchachos desaparecidos.*
	NON-DONDE	*La policía registró mi barrio, de donde proceden los muchachos desaparecidos.*
**39**	RES-QUE	*Los estudiantes decoraron la residencia en la que pasarían los días de navidad.*
	RES-DONDE	*Los estudiantes decoraron la residencia donde pasarían los días de navidad.*
	NON-QUE	*Los estudiantes decoraron su residencia, en la que pasarían los días de navidad.*
	NON-DONDE	*Los estudiantes decoraron su residencia, donde pasarían los días de navidad.*
**40**	RES-QUE	*La universidad cerró el laboratorio del que provenía la fuga radioactiva.*
	RES-DONDE	*La universidad cerró el laboratorio de donde provenía la fuga radioactiva.*
	NON-QUE	*La universidad cerró su laboratorio, del que provenía la fuga radioactiva.*
	NON-DONDE	*La universidad cerró su laboratorio, de donde provenía la fuga radioactiva.*
**41**	RES-QUE	*La ciudad cerró el teatro al que acudían pocos espectadores al año.*
	RES-DONDE	*La ciudad cerró el teatro donde acudían pocos espectadores al año.*
	NON-QUE	*La ciudad cerró su teatro, al que acudían pocos espectadores al año.*
	NON-DONDE	*La ciudad cerró su teatro, donde acudían pocos espectadores al año.*
**42**	RES-QUE	*Pedro regresó al país en el que creció su padre de niño.*
	RES-DONDE	*Pedro regresó al país donde creció su padre de niño.*
	NON-QUE	*Pedro regresó a su país, en el que creció su padre de niño.*
	NON-DONDE	*Pedro regresó a su país, donde creció su padre de niño.*
**43**	RES-QUE	*Javier incendió la academia en la que estudiaba mi primo alemán.*
	RES-DONDE	*Javier incendió la academia donde estudiaba mi primo alemán.*
	NON-QUE	*Javier incendió su academia, en la que estudiaba mi primo alemán.*
	NON-DONDE	*Javier incendió su academia, donde estudiaba mi primo alemán.*
**44**	RES-QUE	*El pueblo valló la carretera por la que cruzaban los animales del monte.*
	RES-DONDE	*El pueblo valló la carretera por donde cruzaban los animales del monte.*
	NON-QUE	*El pueblo valló su carretera, por la que cruzaban los animales del monte.*
	NON-DONDE	*El pueblo valló su carretera, por donde cruzaban los animales del monte.*
**45**	RES-QUE	*La policía registró el restaurante del que escaparon los miembros de la mafia.*
	RES-DONDE	*La policía registró el restaurante de donde escaparon los miembros de la mafia.*
	NON-QUE	*La policía registró mi restaurante, del que escaparon los miembros de la mafia.*
	NON-DONDE	*La policía registró mi restaurante, de donde escaparon los miembros de la mafia.*
**46**	RES-QUE	*La ciudad inauguró el aeropuerto del que partirán los vuelos internacionales.*
	RES-DONDE	*La ciudad inauguró el aeropuerto de donde partirán los vuelos internacionales.*
	NON-QUE	*La ciudad inauguró su aeropuerto, del que partirán los vuelos internacionales.*
	NON-DONDE	*La ciudad inauguró su aeropuerto, de donde partirán los vuelos internacionales.*
**47**	RES-QUE	*El pueblo limpiará el río en el que nadan los niños en verano.*
	RES-DONDE	*El pueblo limpiará el río donde nadan los niños en verano.*
	NON-QUE	*El pueblo limpiará su río, en el que nadan los niños en verano.*
	NON-DONDE	*El pueblo limpiará su río, donde nadan los niños en verano.*
**48**	RES-QUE	*Mis amigos visitaron el zoológico del que escaparon varios animales este mes.*
	RES-DONDE	*Mis amigos visitaron el zoológico de donde escaparon varios animales este mes.*
	NON-QUE	*Mis amigos visitaron el zoológico, del que escaparon varios animales este mes.*
	NON-DONDE	*Mis amigos visitaron el zoológico, de donde escaparon varios animales este mes.*
**49**	RES-QUE	*El panadero cerró el comercio al que acudían los ciudadanos a comprar.*
	RES-DONDE	*El panadero cerró el comercio donde acudían los ciudadanos a comprar.*
	NON-QUE	*El panadero cerró su comercio, al que acudían los ciudadanos a comprar.*

	NON-DONDE	*El panadero cerró su comercio, donde acudían los ciudadanos a comprar.*
**50**	RES-QUE	*El pueblo cerrará el hotel al que iban los turistas de la ciudad.*
	RES-DONDE	*El pueblo cerrará el hotel donde iban los turistas de la ciudad.*
	NON-QUE	*El pueblo cerrará su hotel, al que iban los turistas de la ciudad.*
	NON-DONDE	*El pueblo cerrará su hotel, donde iban los turistas de la ciudad.*
**51**	RES-QUE	*El maestro registró el colegio del que escaparon los niños durante el recreo.*
	RES-DONDE	*El maestro registró el colegio de donde escaparon los niños durante el recreo.*
	NON-QUE	*El maestro registró su colegio, del que escaparon los niños durante el recreo.*
	NON-DONDE	*El maestro registró su colegio, de donde escaparon los niños durante el recreo.*
**52**	RES-QUE	*El ayuntamiento reformó el patio en el que tenían los vecinos sus reuniones.*
	RES-DONDE	*El ayuntamiento reformó el patio donde tenían los vecinos sus reuniones.*
	NON-QUE	*El ayuntamiento reformó su patio, en el que tenían los vecinos sus reuniones.*
	NON-DONDE	*El ayuntamiento reformó su patio, donde tenían los vecinos sus reuniones.*
**53**	RES-QUE	*El baile se organizará en el salón en el que colocaron los camareros las mesas.*
	RES-DONDE	*El baile se organizará en el salón donde colocaron los camareros las mesas.*
	NON-QUE	*El baile se organizará en un salón, en el que colocaron los camareros las mesas.*
	NON-DONDE	*El baile se organizará en un salón, donde colocaron los camareros las mesas.*
**54**	RES-QUE	*Alba vendió el apartamento en el que vivieron sus padres de jóvenes.*
	RES-DONDE	*Alba vendió el apartamento donde vivieron sus padres de jóvenes.*
	NON-QUE	*Alba vendió su apartamento, en el que vivieron sus padres de jóvenes.*
	NON-DONDE	*Alba vendió su apartamento, donde vivieron sus padres de jóvenes.*
**55**	RES-QUE	*La policía registró la vivienda en la que robaron unos ladrones anoche.*
	RES-DONDE	*La policía registró la vivienda donde robaron unos ladrones anoche.*
	NON-QUE	*La policía registró mi vivienda, en la que robaron unos ladrones anoche.*
	NON-DONDE	*La policía registró mi vivienda, donde robaron unos ladrones anoche.*
**56**	RES-QUE	*El príncipe vivía en el castillo al que huyeron sus padres durante el exilio.*
	RES-DONDE	*El príncipe vivía en el castillo donde huyeron sus padres durante el exilio.*
	NON-QUE	*El príncipe vivía en un castillo, al que huyeron sus padres durante el exilio.*
	NON-DONDE	*El príncipe vivía en un castillo, donde huyeron sus padres durante el exilio.*

Section C. Supplementary material for the comprehension questions

Table S1.1. Models' comparisons using Likelihood-ratio test (LRT) for response accuracy in the contrast *Que* versus *El cual*. The asterisk in the last column represents a *p*-value below the significance level of 0.05.

Model's selection

Sampling Units		N total obs = 2688<br>N Subjects = 48; N items = 28					
**Model**	**Model**	**Nested /**	**Fixed**		**Random Effects**	**Model fit**	**LRT Test**

specification	name	simpler Model	Effects added							against nested	
					Subjects	Items	AIC	BIC	LL	df	X2
RE only	Null	-	-		intercept	intercept	770.25	787.94	-382.13		
FE main effects	Main effects	Null	RC type + relativizer		intercept	intercept	772.35	801.83	-381.17	2	1.90
FE two-way interactions	Two-way interaction	Main effects	RC type + relativizer + RCtype*relativizer		intercept	intercept	770.51	805.89	-379.26	1	3.83*
FE two-way interactions	Two-way interaction 2	Two-way interaction	RC type + relativizer + RCtype*relativizer		intercept	Slope for RT	737.42	784.60	-360.71	2	37.08*
FE two-way interactions	Two-way interaction 3	Two-way interaction 2	RC type + relativizer + RCtype*relativizer		Slope for RT	Slope for RT	742.60	801.56	-361.3	2	0

Table S1.2. Results of the linear mixed-effect model for response accuracy in the contrast *Que* versus *El cual*. The top of the table reports estimates/beta values, standard error (SE), confidence intervals (95% CI), *z*-values and levels of significance for fixed effects; the bottom of the table reports variance and standard deviation (S.D.) for the random effects, as well as their correlation.

Fixed Effects						
	Est/Beta	SE	95% CI		z	p
Intercept	3.87	0.34	3.19	4.55	11.22	< .001
RC type	0.69	0.32	0.05	1.34	2.12	.10
Relativizer	0.60	0.31	-0.01	1.21	1.90	.10
RC type*relativizer	-0.95	0.47	-1.87	-0.02	-2.01	.10

Random Effects			
	Variance	S.D.	Correlation
Participant (intercept)	4.823 e-01	0.69	

Items (slope for RT)	1.272 e-06	0.001	-0.9

Table S2.1. Models' comparisons using Likelihood-ratio test (LRT) for response accuracy in the contrast *Que* versus *Quien*. The asterisk in the last column represents a *p*-value below the significance level of 0.05.

Model's selection

Sampling Units	N total obs = 2688<br>N Subjects = 48; N items = 28									
**Model specification**	**Model name**	**Nested / simpler Model**	**Fixed Effects added**	**Random Effects**		**Model fit**			**LRT Test against nested**	
				**Subjects**	**Items**	**AIC**	**BIC**	**LL**	**df**	**X2**
RE only	Null	-	-	intercept	intercept	907.57	925.26	-450.78		
FE main effects	Main effects	Null	RC type + relativizer	intercept	intercept	910.28	939.76	-450.14	2	1.29
FE two-way interactions	Two-way interaction	Main effects	RC type + relativizer + RCtype*relativizer	intercept	intercept	911.22	946.60	-449.61	1	1.05
FE two-way interactions	Two-way interaction 2	Two-way interaction	RC type + relativizer + RCtype*relativizer	intercept	Slope for RT	862.62	909.79	-423.31	2	52.60*
FE two-way interactions	Two-way interaction 3	Two-way interaction 2	RC type + relativizer + RCtype*relativizer	Slope for RT	Slope for RT	859.45	918.41	-419.72	2	7.17*

Table S2.2. Results of the linear mixed-effect model for response accuracy in the contrast *Que* versus *Quien*. The top of the table reports estimates/beta values, standard error (SE), confidence intervals (95% CI), *z*-values and levels of significance for fixed effects; the bottom of the table reports variance and standard deviation (S.D.) for the random effects, as well as their correlation.

Fixed Effects						
	Est/Beta	SE	95% CI		z	p
Intercept	4.65	0.38	3.90	5.41	12.17	< .001
RC type	-0.49	0.32	-1.14	0.14	-1.51	.25
Relativizer	-0.72	0.32	-1.35	-0.09	-2.25	.07
RC type*relativizer	0.66	0.43	-0.18	1.50	1.53	.25
Random Effects						
	Variance	S.D.	Correlation			
Participant (slope for RT)	3.860 e-07	0.0006				
Items (slope for RT)	9.961 e-07	0.0009	-0.77			

Table S3.1. Models' comparisons using Likelihood-ratio test (LRT) for response accuracy in the contrast *Que* versus *Donde*. The asterisk in the last column represents a *p*-value below the significance level of 0.05.

Model's selection

Sampling Units	N total obs = 2688<br>N Subjects = 48; N items = 28									
**Model specification**	**Model name**	**Nested / simpler Model**	**Fixed Effects added**	**Random Effects**		**Model fit**			**LRT Test against nested**	
				**Subjects**	**Items**	**AIC**	**BIC**	**LL**	**df**	**X2**
RE only	Null	-	-	intercept	intercept	929.29	946.98	-461.64		
FE main effects	Main effects	Null	RC type + relativizer	intercept	intercept	933.21	962.69	-461.61	2	0.07
FE two-way interactions	Two-way interaction	Main effects	RC type + relativizer + RCtype*relativizer	intercept	intercept	929.70	965.08	-458.85	1	5.51*
FE two-way interactions	Two-way interaction 2	Two-way interaction	RC type + relativizer + RCtype*relativizer	intercept	Slope for RT	881.88	929.05	-432.94	2	51.81*

FE two-way interactions	Two-way interaction 3	Two-way interaction 2	RC type + relativizer + RCtype*relativizer		Slope for RT	Slope for RT	881.36	940.33	-430.68	2	4.51

Table S3.2. Results of the linear mixed-effect model for response accuracy in the contrast *Que* versus *Donde*. The top of the table reports estimates/beta values, standard error (SE), confidence intervals (95% CI), *z*-values and levels of significance for fixed effects; the bottom of the table reports variance and standard deviation (S.D.) for the random effects, as well as their correlation.

Fixed Effects						
	Est/Beta	SE	95% CI		z	p
Intercept	3.91	0.33	3.25	4.56	11.66	< .001
RC type	0.51	0.30	-0.08	1.12	1.68	.18
Relativizer	0.38	0.30	-0.20	0.98	1.28	.19
RC type*relativizer	-1.09	0.43	-1.94	-0.25	-2.54	.03

Random Effects			
	Variance	S.D.	Correlation
Participant (slope for RT)	5.529 e-07	0.0007	
Items (slope for RT)	1.057 e-06	0.001	-0.91

Section D. Supplementary material of the eye-movement measures for the contrast *Que* vs. *El cual*

Table S1.1. Mean reading times by condition at R3, R4 and R5 for the contrast *Que* vs. *El cual*. Eye-movement measures are first-fixation rate (FFR), first fixation (FF), first-pass duration (FPD), first-pass regression (FPR), quasi first-pass reading time (QFP), second-pass duration (SPD). The numbers in parentheses indicate standard deviation.

Region	Measure	RESTRICTIVE		NON-RESTRICTIVE	
		QUE	*EL CUAL*	*QUE*	*EL CUAL*
R3	FFR (%)	0.92 (0.27)	0.91 (0.29)	0.92 (0.28)	0.90 (0.30)

	FF (ms)	243.68 (128.79)	242.68 (132.78)	246.38 (141.75)	246.69 (149.88)
	FPD (ms)	273.97 (166.53)	270.78 (162.18)	273.04 (170.03)	272.08 (176.35)
	FPR (%)	0.16 (0.36)	0.14 (0.35)	0.23 (0.42)	0.22 (0.42)
	QFP (ms)	290.15 (194.9)	285.77 (182.53)	287.56 (187.82)	282.25 (186.29)
	SPD (ms)	67.4 (199.71)	55.57 (180.25)	104.33 (357.39)	73.16 (173.58)
R4	FFR (%)	0.86 (0.35)	0.94 (0.24)	0.90 (0.30)	0.90 (0.30)
	FF (ms)	229.46 (147.31)	281.1 (152.9)	228.59 (136.15)	265.84 (160.27)
	FPD (ms)	237.84 (159.98)	299.52 (167.85)	234.34 (147.04)	276.62 (170.37)
	FPR (%)	0.12 (0.33)	0.19 (0.39)	0.11 (0.32)	0.13 (0.33)
	QFP (ms)	241.69 (162.75)	309.75 (180.8)	240.13 (156.78)	282.91 (177.19)
	SPD (ms)	38.66 (131.26)	71.15 (198.74)	35.57 (129.77)	46.16 (162.04)
R5	FFR (%)	0.82 (0.39)	0.85 (0.35)	0.81 (0.40)	0.85 (0.35)
	FF (ms)	179.65 (114.04)	184.15 (98.68)	170.97 (98.51)	181.77 (96.96)
	FPD (ms)	232.18 (184.32)	225.74 (155.37)	211.76 (149.14)	223.29 (154.24)
	FPR (%)	0.09 (0.29)	0.11 (0.31)	0.08 (0.28)	0.07 (0.25)
	QFP (ms)	242.63 (198.97)	242.69 (185.79)	224.06 (168.22)	234.01 (170.31)
	SPD (ms)	44.07 (167.33)	66.14 (263.69)	42.74 (194.29)	37.26 (198.78)

Section S1.2. Models' reports for eye-movement measures at R3

Table S2.1. Models' comparisons using Likelihood-ratio test (LRT) for first fixation at R3 in the contrast *Que* versus *El cual*. The asterisk in the last column represents a *p*-value below the significance level of 0.05.

Model's selection

Sampling Units	N total obs = 2688<br>N Subjects = 48; N items = 28										
**Model specification**	**Model name**	**Nested / simpler Model**	**Fixed Effects added**		**Random Effects**		**Model fit**			**LRT Test against nested**	
					**Subjects**	**Items**	**AIC**	**BIC**	**LL**	**df**	**X2**
RE only	Null	-	-		Slope for RC type + relativizer	Slope for RC type + relativizer	33686	33769	-16829		
FE main effects	Main effects	Null	RC type + relativizer		Slope for RC type + relativizer	Slope for RC type + relativizer	33690	33784	-16829	2	0.32

FE two-way interactions	Two-way interaction	Main effects	RC type + relativizer + RCtype*relativizer		Slope for RC type + relativizer	Slope for RC type + relativizer	33692	33792	-16829	1	0

Table S2.2. Results of the linear mixed-effect model for first fixation at R3 in the contrast *Que* versus *El cual*. The top of the table reports estimates/beta values, standard error (SE), confidence intervals (95% CI), *t*-values and levels of significance for fixed effects; the bottom of the table reports variance and standard deviation (S.D.) for the random effects, as well as their correlation.

Fixed Effects						
	Est/Beta	SE	95% CI		t	p
Intercept	242.68	10.906	221.30	264.06	22.25	< .001
RC type	4.01	7.568	-10.82	18.84	0.53	1.00
Relativizer	0.99	6.759	-12.25	14.24	0.14	1.00
RC type*relativizer	-1.30	9.426	-19.78	17.16	-0.13	1.00
Random Effects						
				Variance	S.D.	Correlation
Participant (slope for RC type)				593.40	24.36	
Participant (slope for relativizer)				37.79	6.14	0.39
Items (slope for RC type)				13.52	3.67	
Items (slope for relativizer)				13.23	3.63	-1.00

Table S2.3. Models' comparisons using Likelihood-ratio test (LRT) for the first-fixation rate at R3 in the contrast *Que* versus *El cual*. The asterisk in the last column represents a *p*-value below the significance level of 0.05.

Model's selection

Sampling Units	N total obs = 2688<br>N Subjects = 48; N items = 28										
**Model specification**	**Model name**	**Nested / simpler Model**	**Fixed Effects added**		**Random Effects**		**Model fit**			**LRT Test against nested**	
					**Subjects**	**Items**	**AIC**	**BIC**	**LL**	**df**	**X2**

RE only	Null	-	-		Slope for RC type + relativizer	Slope for RC type + relativizer	1466.8	1543.5	-720.41		
FE main effects	Main effects	Null	RC type + relativizer		Slope for RC type + relativizer	Slope for RC type + relativizer	1470.0	1558.4	-719.98	2	0.85
FE two-way interactions	Two-way interaction	Main effects	RC type + relativizer + RCtype*relativizer		Slope for RC type + relativizer	Slope for RC type + relativizer	1471.8	1566.1	-719.88	1	0.20

Table S2.4. Results of the linear mixed-effect model for first-fixation rate at R3 in the contrast *Que* versus *El cual*. The top of the table reports estimates/beta values, standard error (SE), confidence intervals (95% CI), *z*-values and levels of significance for fixed effects; the bottom of the table reports variance and standard deviation (S.D.) for the random effects, as well as their correlation.

Fixed Effects						
	Est/Beta	SE	95% CI		z	p
Intercept	3.13	0.31	2.50	3.76	9.80	< .001
RC type	-0.30	0.28	-0.85	0.24	-1.07	.84
Relativizer	-0.05	0.27	-0.58	0.47	-0.21	1.00
RC type*relativizer	0.14	0.30	-0.46	0.74	0.45	1.00
Random Effects						
				Variance	S.D.	Correlation
Participant (slope for RC type)				0.53	0.72	
Participant (slope for relativizer)				0.20	0.45	0.65
Items (slope for RC type)				0.004	0.06	
Items (slope for relativizer)				0.03	0.18	-1.00

Table S2.5. Models' comparisons using Likelihood-ratio test (LRT) for first-pass duration at R3 in the contrast *Que* versus *El cual*. The asterisk in the last column represents a *p*-value below the significance level of 0.05.

Model's selection

Sampling Units	N total obs = 2688<br>N Subjects = 48; N items = 28										
**Model specification**	**Model name**	**Nested / simpler Model**	**Fixed Effects added**		**Random Effects**		**Model fit**			**LRT Test against nested**	
					**Subjects**	**Items**	**AIC**	**BIC**	**LL**	**df**	**X2**
RE only	Null	-	-		Slope for RC type + relativizer	Slope for RC type + relativizer	34731	34814	-17352		
FE main effects	Main effects	Null	RC type + relativizer		Slope for RC type + relativizer	Slope for RC type + relativizer	34735	34830	-17352	2	0.11
FE two-way interactions	Two-way interaction	Main effects	RC type + relativizer + RCtype*relativizer		Slope for RC type + relativizer	Slope for RC type + relativizer	34737	34837	-17352	1	0.03

Table S2.6. Results of the linear mixed-effect model for first-pass duration at R3 in the contrast *Que* versus *El cual*. The top of the table reports estimates/beta values, standard error (SE), confidence intervals (95% CI), *t*-values and levels of significance for fixed effects; the bottom of the table reports variance and standard deviation (S.D.) for the random effects, as well as their correlation.

Fixed Effects						
	Est/Beta	SE	95% CI		t	p
Intercept	270.78	13.29	244.71	296.84	20.36	< .001
RC type	1.30	9.69	-17.69	20.30	0.13	1.00
Relativizer	3.19	8.41	-13.29	19.67	0.37	1.00
RC type*relativizer	-2.22	11.42	-24.60	20.15	-0.19	1.00
Random Effects						
				Variance	S.D.	Correlation
Participant (slope for RC type)				1066.62	32.66	
Participant (slope for relativizer)				71.23	8.44	0.76
Items (slope for RC type)				183.61	13.55	
Items (slope for relativizer)				113.90	10.67	-1.00

Table S2.7. Models' comparisons using Likelihood-ratio test (LRT) for first-pass regression at R3 in the contrast *Que* versus *El cual*. The asterisk in the last column represents a *p*-value below the significance level of 0.05.

Model's selection

Sampling Units	N total obs = 2688<br>N Subjects = 48; N items = 28										
**Model specification**	**Model name**	**Nested / simpler Model**	**Fixed Effects added**		**Random Effects**		**Model fit**			**LRT Test against nested**	
					**Subjects**	**Items**	**AIC**	**BIC**	**LL**	**df**	**X2**
RE only	Null	-	-		Slope for RC type + relativizer	Slope for RC type + relativizer	2447.9	2524.6	-1211.0		
FE main effects	Main effects	Null	RC type + relativizer		Slope for RC type + relativizer	Slope for RC type + relativizer	2439.4	2527.9	-1204.7	2	12.48*
FE two-way interactions	Two-way interaction	Main effects	RC type + relativizer + RCtype*relativizer		Slope for RC type + relativizer	Slope for RC type + relativizer	2441.4	2535.8	-1204.7	1	0

Table S2.8. Results of the linear mixed-effect model for first-pass regression at R3 in the contrast *Que* versus *El cual*. The top of the table reports estimates/beta values, standard error (SE), confidence intervals (95% CI), *z*-values and levels of significance for fixed effects; the bottom of the table reports variance and standard deviation (S.D.) for the random effects, as well as their correlation.

Fixed Effects						
	Est/Beta	SE	95% CI		z	p
Intercept	-2.07	0.197	-2.46	-1.68	-10.52	< .001
RC type	0.50	0.17	0.16	0.84	2.88	.01
Relativizer	0.22	0.17	-0.12	0.57	1.27	.4
RC type*relativizer	-0.06	0.21	-0.48	0.35	-0.3	.76
Random Effects						

	Variance	S.D.	Correlation
Participant (slope for RC type)	0.02	0.16	
Participant (slope for relativizer)	0.08	0.29	-0.9
Items (slope for RC type)	0.04	0.21	
Items (slope for relativizer)	0.0008	0.02	-0.88

Table S2.9. Models' comparisons using Likelihood-ratio test (LRT) for quasi first-pass reading time at R3 in the contrast *Que* versus *El cual*. The asterisk in the last column represents a *p*-value below the significance level of 0.05.

Model's selection

Sampling Units	N total obs = 2688<br>N Subjects = 48; N items = 28										
**Model specification**	**Model name**	**Nested / simpler Model**	**Fixed Effects added**		**Random Effects**		**Model fit**			**LRT Test against nested**	
					**Subjects**	**Items**	**AIC**	**BIC**	**LL**	**df**	**X2**
RE only	Null	-	-		Slope for RC type + relativizer	Slope for RC type + relativizer	35232	35315	-17602		
FE main effects	Main effects	Null	RC type + relativizer		Slope for RC type + relativizer	Slope for RC type + relativizer	35235	35330	-17602	2	0.52
FE two-way interactions	Two-way interaction	Main effects	RC type + relativizer + RCtype*relativizer		Slope for RC type + relativizer	Slope for RC type + relativizer	35237	35338	-17602	1	0.005

Table S2.10. Results of the linear mixed-effect model for quasi first-pass reading time at R3 in the contrast *Que* versus *El cual*. The top of the table reports estimates/beta values, standard error (SE), confidence intervals (95% CI), *t*-values and levels of significance for fixed effects; the bottom of the table reports variance and standard deviation (S.D.) for the random effects, as well as their correlation.

Fixed Effects					
	Est/Beta	SE	95% CI	t	p

Intercept	285.76	15.45	255.47	316.06	18.48	< .001
RC type	-3.51	10.98	-25.04	18.00	-0.32	1.00
Relativizer	4.38	9.61	-14.45	23.22	0.45	1.00
RC type*relativizer	0.92	12.49	-23.56	25.41	0.07	1.00

Random Effects			
	Variance	S.D.	Correlation
Participant (slope for RC type)	1460.1	38.21	
Participant (slope for relativizer)	26.3	5.12	0.26
Items (slope for RC type)	338.3	18.39	
Items (slope for relativizer)	384.7	19.61	-1.00

Table S2.11. Models' comparisons using Likelihood-ratio test (LRT) for second-pass duration at R3 in the contrast *Que* versus *El cual*. The asterisk in the last column represents a *p*-value below the significance level of 0.05.

Model's selection

Sampling Units	N total obs = 2688<br>N Subjects = 48; N items = 28									
**Model specification**	**Model name**	**Nested / simpler Model**	**Fixed Effects added**	**Random Effects**		**Model fit**			**LRT Test against nested**	
				**Subjects**	**Items**	**AIC**	**BIC**	**LL**	**df**	**X2**
RE only	Null	-	-	Slope for RC type + relativizer	Slope for RC type + relativizer	37009	37092	-18491		
FE main effects	Main effects	Null	RC type + relativizer	Slope for RC type + relativizer	Slope for RC type + relativizer	37008	37102	-18488	2	5.50
FE two-way interactions	Two-way interaction	Main effects	RC type + relativizer + RCtype*relativizer	Slope for RC type + relativizer	Slope for RC type + relativizer	37009	37109	-18487	1	1.18

Table S2.12. Results of the linear mixed-effect model for second-pass duration at R3 in the contrast *Que* versus *El cual*. The top of the table reports estimates/beta values,

standard error (SE), confidence intervals (95% CI), *t*-values and levels of significance for fixed effects; the bottom of the table reports variance and standard deviation (S.D.) for the random effects, as well as their correlation.

Fixed Effects						
	Est/Beta	SE	95% CI		t	p
Intercept	55.57	8.91	38.10	73.03	6.23	< .001
RC type	17.59	15.15	-12.10	47.29	1.16	.73
Relativizer	11.84	14.84	-17.25	40.92	0.79	.73
RC type*relativizer	19.34	17.80	-15.54	54.22	1.08	.73
Random Effects						
				Variance	S.D.	Correlation
Participant (slope for RC type)				2701.13	51.97	
Participant (slope for relativizer)				2302.32	47.98	1.00
Items (slope for RC type)				418.30	20.45	
Items (slope for relativizer)				387.32	19.68	1.00

Section D.3. Models' reports for eye-movement measures at R4

Table S3.1. Models' comparisons using Likelihood-ratio test (LRT) for first fixation at R4 in the contrast *Que* versus *El cual*. The asterisk in the last column represents a *p*-value below the significance level of 0.05.

Model's selection

Sampling Units	**N total obs = 2688**<br>**N Subjects = 48; N items = 28**									
**Model specification**	**Model name**	**Nested / simpler Model**	**Fixed Effects added**	**Random Effects**		**Model fit**			**LRT Test against nested**	
				**Subjects**	**Items**	**AIC**	**BIC**	**LL**	**df**	**X2**
RE only	Null	-	**-**	**Slope for RC type + relativizer**	Slope for RC type + relativizer	33827	33910	-16900		
FE main effects	Main effects	Null	**RC type + relativizer**	**Slope for RC type + relativizer**	Slope for RC type + relativizer	33844	33938	-16906	2	0

FE two-way interactions	Two-way interaction	Main effects	**RC type + relativizer + RCtype*relativizer**		**Slope for RC type + relativizer**	Slope for RC type + relativizer	33844	33944	-16905	1	2.20

Table S3.2. Results of the linear mixed-effect model for first fixation at R4 in the contrast *Que* versus *El cual*. The top of the table reports estimates/beta values, standard error (SE), confidence intervals (95% CI), *t*-values and levels of significance for fixed effects; the bottom of the table reports variance and standard deviation (S.D.) for the random effects, as well as their correlation.

Fixed Effects						
	Est/Beta	SE	95% CI		t	p
Intercept	281.10	13.14	255.34	306.85	21.39	< .001
RC type	-15.25	7.94	-30.82	0.32	-1.92	.11
Relativizer	-51.64	8.48	-68.27	-35.00	-6.08	< .001
RC type*relativizer	14.38	9.67	-4.58	33.35	1.48	.13
Random Effects						
				Variance	S.D.	Correlation
Participant (slope for RC type)				342.0	18.49	
Participant (slope for relativizer)				360.7	18.99	0.54
Items (slope for RC type)				256.9	16.03	
Items (slope for relativizer)				495.7	22.26	0.68

Table S3.3. Models' comparisons using Likelihood-ratio test (LRT) for first-fixation rate at R4 in the contrast *Que* versus *El cual*. The asterisk in the last column represents a *p*-value below the significance level of 0.05.

Model's selection

Sampling Units	N total obs = 2688<br>N Subjects = 48; N items = 28										
**Model specification**	**Model name**	**Nested / simpler Model**	**Fixed Effects added**		**Random Effects**		**Model fit**			**LRT Test against nested**	
					**Subjects**	**Items**	**AIC**	**BIC**	**LL**	**df**	**X2**

RE only	Null	-	-		Slope for RC type + relativizer	Slope for RC type + relativizer	1568.1	1644.8	-771.05		
FE main effects	Main effects	Null	RC type + relativizer		Slope for RC type + relativizer	Slope for RC type + relativizer	1567.7	1656.1	-768.84	2	4.42
FE two-way interactions	Two-way interaction	Main effects	RC type + relativizer + RCtype*relativizer		Slope for RC type + relativizer	Slope for RC type + relativizer	1556.5	1650.9	-762.26	1	13.15*

Table S3.4. Results of the linear mixed-effect model for first-fixation rate at R4 in the contrast *Que* versus *El cual*. The top of the table reports estimates/beta values, standard error (SE), confidence intervals (95% CI), *z*-values and levels of significance for fixed effects; the bottom of the table reports variance and standard deviation (S.D.) for the random effects, as well as their correlation.

Fixed Effects						
	Est/Beta	SE	95% CI		z	p
Intercept	3.46	0.31	2.83	4.08	10.89	< .001
RC type	-0.72	0.27	-1.26	-0.17	-2.58	.009
Relativizer	-1.03	0.26	-1.55	-0.52	-3.94	< .001
RC type*relativizer	1.08	0.29	0.51	1.65	3.72	< .001
Random Effects						
				Variance	S.D.	Correlation
Participant (slope for RC type)				0.15	0.39	
Participant (slope for relativizer)				0.01	0.14	0.57
Items (slope for RC type)				0.01	0.11	
Items (slope for relativizer)				0.01	0.13	-0.92

Table S3.5. Models' comparisons using Likelihood-ratio test (LRT) for first-pass duration at R4 in the contrast *Que* versus *El cual*. The asterisk in the last column represents a *p*-value below the significance level of 0.05.

Model's selection

Sampling Units	N total obs = 2688

	N Subjects = 48; N items = 28									
**Model specification**	**Model name**	**Nested / simpler Model**	**Fixed Effects added**	**Random Effects**		**Model fit**			**LRT Test against nested**	
				**Subjects**	**Items**	**AIC**	**BIC**	**LL**	**df**	**X2**
RE only	Null	-	-	Slope for RC type + relativizer	Slope for RC type + relativizer	34269	34352	-17121		
FE main effects	Main effects	Null	RC type + relativizer	Slope for RC type + relativizer	Slope for RC type + relativizer	34270	34364	-17119	2	3.22
FE two-way interactions	Two-way interaction	Main effects	RC type + relativizer + RCtype*relativizer	Slope for RC type + relativizer	Slope for RC type + relativizer	34268	34369	-17117	1	3.43

Table S3.6. Results of the linear mixed-effect model for first-pass duration at R4 in the contrast *Que* versus *El cual*. The top of the table reports estimates/beta values, standard error (SE), confidence intervals (95% CI), *t*-values and levels of significance for fixed effects; the bottom of the table reports variance and standard deviation (S.D.) for the random effects, as well as their correlation.

Fixed Effects						
	Est/Beta	SE	95% CI		t	p
Intercept	299.52	14.65	270.80	328.24	20.44	< .001
RC type	-22.90	8.73	-40.02	-5.77	-2.62	.017
Relativizer	-61.68	9.22	-79.77	-43.59	-6.68	< .001
RC type*relativizer	19.40	10.46	-1.11	39.91	1.85	.06

Random Effects			
	Variance	S.D.	Correlation
Participant (slope for RC type)	552.2	23.50	
Participant (slope for relativizer)	379.0	19.47	0.46
Items (slope for RC type)	281.2	16.77	
Items (slope for relativizer)	629.5	25.09	0.52

Table S3.7. Models' comparisons using Likelihood-ratio test (LRT) for first-pass regression at R4 in the contrast *Que* versus *El cual*. The asterisk in the last column represents a *p*-value below the significance level of 0.05.

Model's selection

Sampling Units	N total obs = 2688<br>N Subjects = 48; N items = 28									
**Model specification**	**Model name**	**Nested / simpler Model**	**Fixed Effects added**	**Random Effects**		**Model fit**			**LRT Test against nested**	
				**Subjects**	**Items**	**AIC**	**BIC**	**LL**	**df**	**X2**
RE only	Null	-	-	Slope for RC type + relativizer	Slope for RC type + relativizer	1997.3	2074.0	-985.66		
FE main effects	Main effects	Null	RC type + relativizer	Slope for RC type + relativizer	Slope for RC type + relativizer	1991.3	2079.8	-980.65	2	10.01*
FE two-way interactions	Two-way interaction	Main effects	RC type + relativizer + RCtype*relativizer	Slope for RC type + relativizer	Slope for RC type + relativizer	1990.4	2084.8	-979.21	1	2.87

Table S3.8. Results of the linear mixed-effect model for first-pass regression at R4 in the contrast *Que* versus *El cual*. The top of the table reports estimates/beta values, standard error (SE), confidence intervals (95% CI), *z*-values and levels of significance for fixed effects; the bottom of the table reports variance and standard deviation (S.D.) for the random effects, as well as their correlation.

Fixed Effects						
	Est/Beta	SE	95% CI		z	p
Intercept	-1.69	0.19	-2.06	-1.31	-8.83	< .001
RC type	-0.65	0.20	-1.06	-0.24	-3.10	.003
Relativizer	-0.60	0.18	-0.97	-0.24	-3.26	.003
RC type*relativizer	0.45	0.24	-0.02	0.94	1.86	.06
Random Effects						

	Variance	S.D.	Correlation
Participant (slope for RC type)	0.08	0.29	
Participant (slope for relativizer)	0.03	0.17	-0.84
Items (slope for RC type)	0.23	0.48	
Items (slope for relativizer)	0.07	0.27	0.27

Table S3.9. Models' comparisons using Likelihood-ratio test (LRT) for quasi first-pass reading time at R4 in the contrast *Que* versus *El cual*. The asterisk in the last column represents a *p*-value below the significance level of 0.05.

Model's selection

Sampling Units	N total obs = 2688<br>N Subjects = 48; N items = 28										
**Model specification**	**Model name**	**Nested / simpler Model**	**Fixed Effects added**		**Random Effects**		**Model fit**			**LRT Test against nested**	
					**Subjects**	**Items**	**AIC**	**BIC**	**LL**	**df**	**X2**
RE only	Null	-	-		Slope for RC type + relativizer	Slope for RC type + relativizer	34524	34607	-17248		
FE main effects	Main effects	Null	RC type + relativizer		Slope for RC type + relativizer	Slope for RC type + relativizer	34495	34590	-17232	2	32.74*
FE two-way interactions	Two-way interaction	Main effects	RC type + relativizer + RCtype*relativizer		Slope for RC type + relativizer	Slope for RC type + relativizer	34492	34592	-17229	1	5.36*

Table S3.10. Results of the linear mixed-effect model for quasi first-pass reading time at R4 in the contrast *Que* versus *El cual*. The top of the table reports estimates/beta values, standard error (SE), confidence intervals (95% CI), *t*-values and levels of significance for fixed effects; the bottom of the table reports variance and standard deviation (S.D.) for the random effects, as well as their correlation.

Fixed Effects					
	Est/Beta	SE	95% CI	t	p

Intercept	309.75	16.14	278.11	341.39	19.19	< .001
RC type	-26.83	9.05	-44.57	-9.09	-2.96	.006
Relativizer	-68.06	9.72	-87.12	-48.99	-6.99	< .001
RC type*relativizer	25.28	10.91	3.89	46.66	2.31	.02
Random Effects						
				Variance	S.D.	Correlation
Participant (slope for RC type)				827.2	28.76	
Participant (slope for relativizer)				704.5	26.54	0.47
Items (slope for RC type)				144.9	12.04	
Items (slope for relativizer)				571.1	23.91	0.93

Table S3.11. Models' comparisons using Likelihood-ratio test (LRT) for second-pass duration at R4 in the contrast *Que* versus *El cual*. The asterisk in the last column represents a *p*-value below the significance level of 0.05.

Model's selection

Sampling Units	N total obs = 2688<br>N Subjects = 48; N items = 28									
**Model specification**	**Model name**	**Nested / simpler Model**	**Fixed Effects added**	**Random Effects**		**Model fit**			**LRT Test against nested**	
				**Subjects**	**Items**	**AIC**	**BIC**	**LL**	**df**	**X2**
RE only	Null	-	-	Slope for RC type + relativizer	Slope for RC type + relativizer	34783	34865	-17377		
FE main effects	Main effects	Null	RC type + relativizer	Slope for RC type + relativizer	Slope for RC type + relativizer	34778	34873	-17373	2	8.50*
FE two-way interactions	Two-way interaction	Main effects	RC type + relativizer + RCtype*relativizer	Slope for RC type + relativizer	Slope for RC type + relativizer	34777	34877	-17372	1	3.46

Table S3.12. Results of the linear mixed-effect model for second-pass duration at R4 in the contrast *Que* versus *El cual*. The top of the table reports estimates/beta values, standard error (SE), confidence intervals (95% CI), *t*-values and levels of significance

for fixed effects; the bottom of the table reports variance and standard deviation (S.D.) for the random effects, as well as their correlation.

Fixed Effects						
	Est/Beta	SE	95% CI		t	p
Intercept	71.15	11.01	49.57	92.74	6.46	< .001
RC type	-24.99	9.58	-43.77	-6.20	-2.60	.01
Relativizer	-32.48	10.08	-52.26	-12.71	-3.22	.003
RC type*relativizer	21.89	11.70	-1.04	44.84	1.87	.06

Random Effects			
	Variance	S.D.	Correlation
Participant (slope for RC type)	331.8	18.21	
Participant (slope for relativizer)	953.1	30.87	-0.13
Items (slope for RC type)	460.0	21.45	
Items (slope for relativizer)	375.5	19.38	0.75

Section D.4. Models' reports for eye-movement measures at R5

Table S4.1. Models' comparisons using Likelihood-ratio test (LRT) for first fixation at R5 in the contrast *Que* versus *El cual*. The asterisk in the last column represents a *p*-value below the significance level of 0.05.

Model's selection

Sampling Units	N total obs = 2688<br>N Subjects = 48; N items = 28										
**Model specification**	**Model name**	**Nested / simpler Model**	**Fixed Effects added**		**Random Effects**		**Model fit**			**LRT Test against nested**	
					**Subjects**	**Items**	**AIC**	**BIC**	**LL**	**df**	**X2**
RE only	Null	-	-		Slope for RC type + relativizer	Slope for RC type + relativizer	31956	32038	-15964		
FE main effects	Main effects	Null	RC type + relativizer		Slope for RC type + relativizer	Slope for RC type + relativizer	31954	32049	-15961	2	5.30

FE two-way interactions	Two-way interaction	Main effects	RC type + relativizer + RCtype*relativizer	Slope for RC type + relativizer	Slope for RC type + relativizer	31956	32056	-15961	1	0.85

Table S4.2. Results of the linear mixed-effect model for first fixation at R5 in the contrast *Que* versus *El cual*. The top of the table reports estimates/beta values, standard error (SE), confidence intervals (95% CI), *t*-values and levels of significance for fixed effects; the bottom of the table reports variance and standard deviation (S.D.) for the random effects, as well as their correlation.

Fixed Effects						
	Est/Beta	SE	95% CI		t	p
Intercept	184.15	8.63	167.23	201.08	21.32	< .001
RC type	-2.38	5.70	-13.56	8.79	-0.41	1
Relativizer	-4.50	5.19	-14.67	5.66	-0.86	1
RC type*relativizer	-6.29	6.81	-19.65	7.07	-0.92	1
Random Effects						
				Variance	S.D.	Correlation
Participant (slope for RC type)				47.96	6.92	
Participant (slope for relativizer)				27.78	5.27	1.00
Items (slope for RC type)				232.22	15.23	
Items (slope for relativizer)				87.05	9.33	-0.34

Table S4.3. Models' comparisons using Likelihood-ratio test (LRT) for first-fixation rate at R5 in the contrast *Que* versus *El cual*. The asterisk in the last column represents a *p*-value below the significance level of 0.05.

Model's selection

Sampling Units	N total obs = 2688<br>N Subjects = 48; N items = 28									
**Model specification**	**Model name**	**Nested / simpler Model**	**Fixed Effects added**	**Random Effects**		**Model fit**			**LRT Test against nested**	
				**Subjects**	**Items**	**AIC**	**BIC**	**LL**	**df**	**X2**

RE only	Null	-	-		Slope for RC type + relativizer	Slope for RC type + relativizer	2147.1	2223.7	-1060.5		
FE main effects	Main effects	Null	RC type + relativizer		Slope for RC type + relativizer	Slope for RC type + relativizer	2147.4	2235.9	-1058.7	2	3.64
FE two-way interactions	Two-way interaction	Main effects	RC type + relativizer + RCtype*relativizer		Slope for RC type + relativizer	Slope for RC type + relativizer	2148.8	2243.1	-1058.4	1	0.69

Table S4.4. Results of the linear mixed-effect model for first-fixation rate at R5 in the contrast *Que* versus *El cual*. The top of the table reports estimates/beta values, standard error (SE), confidence intervals (95% CI), *z*-values and levels of significance for fixed effects; the bottom of the table reports variance and standard deviation (S.D.) for the random effects, as well as their correlation.

Fixed Effects						
	Est/Beta	SE	95% CI		z	p
Intercept	2.13	0.22	1.69	2.56	9.66	< .001
RC type	0.16	0.21	-0.25	0.58	0.78	.79
Relativizer	-0.24	0.20	-0.64	0.16	-1.17	.71
RC type*relativizer	-0.19	0.23	-0.66	0.26	-0.84	.79
Random Effects						
	Variance	S.D.	Correlation			
Participant (slope for RC type)	0.09	0.31				
Participant (slope for relativizer)	0.14	0.37	0.34			
Items (slope for RC type)	0.19	0.44				
Items (slope for relativizer)	0.18	0.42	-0.99			

Table S4.5. Models' comparisons using Likelihood-ratio test (LRT) for first-pass duration at R5 in the contrast *Que* versus *El cual*. The asterisk in the last column represents a *p*-value below the significance level of 0.05.

Model's selection

Sampling Units	N total obs = 2688

	N Subjects = 48; N items = 28											
**Model specification**	**Model name**	**Nested / simpler Model**	**Fixed Effects added**		**Random Effects**		**Model fit**			**LRT Test against nested**	
					**Subjects**	**Items**	**AIC**	**BIC**	**LL**	**df**	**X2**
RE only	Null	-	-		Slope for RC type + relativizer	Slope for RC type + relativizer	34234	34317	-17103		
FE main effects	Main effects	Null	RC type + relativizer		Slope for RC type + relativizer	Slope for RC type + relativizer	34235	34329	-17101	2	3.72
FE two-way interactions	Two-way interaction	Main effects	RC type + relativizer + RCtype*relativizer		Slope for RC type + relativizer	Slope for RC type + relativizer	34234	34334	-17100	1	3.01

Table S4.6. Results of the linear mixed-effect model for first-pass duration at R5 in the contrast *Que* versus *El cual*. The top of the table reports estimates/beta values, standard error (SE), confidence intervals (95% CI), *t*-values and levels of significance for fixed effects; the bottom of the table reports variance and standard deviation (S.D.) for the random effects, as well as their correlation.

Fixed Effects						
	Est/Beta	SE	95% CI		t	p
Intercept	225.74	14.97	196.39	255.10	15.07	< .001
RC type	-2.45	8.60	-19.32	14.41	-0.28	.88
Relativizer	6.44	8.34	-9.92	22.80	0.77	.88
RC type*relativizer	-17.96	10.35	-38.25	2.32	-1.73	.24

Random Effects			
	Variance	S.D.	Correlation
Participant (slope for RC type)	209.4	14.47	
Participant (slope for relativizer)	226.9	15.06	-0.95
Items (slope for RC type)	452.3	21.27	
Items (slope for relativizer)	318.8	17.85	-0.84

Table S4.7. Models' comparisons using Likelihood-ratio test (LRT) for first-pass regression at R5 in the contrast *Que* versus *El cual*. The asterisk in the last column represents a *p*-value below the significance level of 0.05.

Model's selection

Sampling Units	N total obs = 2688<br>N Subjects = 48; N items = 28										
**Model specification**	**Model name**	**Nested / simpler Model**	**Fixed Effects added**		**Random Effects**		**Model fit**			**LRT Test against nested**	
					**Subjects**	**Items**	**AIC**	**BIC**	**LL**	**df**	**X2**
RE only	Null	-	-		Slope for RC type + relativizer	Slope for RC type + relativizer	1538.8	1615.5	-756.42		
FE main effects	Main effects	Null	RC type + relativizer		Slope for RC type + relativizer	Slope for RC type + relativizer	1539.1	1627.6	-754.57	2	3.69
FE two-way interactions	Two-way interaction	Main effects	RC type + relativizer + RCtype*relativizer		Slope for RC type + relativizer	Slope for RC type + relativizer	1539.4	1633.8	-753.72	1	1.70

Table S4.8. Results of the linear mixed-effect model for first-pass regression at R5 in the contrast *Que* versus *El cual*. The top of the table reports estimates/beta values, standard error (SE), confidence intervals (95% CI), *z*-values and levels of significance for fixed effects; the bottom of the table reports variance and standard deviation (S.D.) for the random effects, as well as their correlation.

Fixed Effects						
	Est/Beta	SE	95% CI		z	p
Intercept	-2.47	0.21	-2.90	-2.04	-11.26	< .001
RC type	-0.54	0.24	-1.02	-0.07	-2.28	.06
Relativizer	-0.05	0.22	-0.49	0.38	-0.22	.82
RC type*relativizer	0.40	0.29	-0.17	0.97	1.37	.33
Random Effects						

	Variance	S.D.	Correlation
Participant (slope for RC type)	0.09	0.31	
Participant (slope for relativizer)	0.23	0.48	0.51
Items (slope for RC type)	0.003	0.06	
Items (slope for relativizer)	0.004	0.06	0.99

Table S4.9. Models' comparisons using Likelihood-ratio test (LRT) for quasi first-pass reading time at R5 in the contrast *Que* versus *El cual*. The asterisk in the last column represents a *p*-value below the significance level of 0.05.

Model's selection

Sampling Units	N total obs = 2688<br>N Subjects = 48; N items = 28										
**Model specification**	**Model name**	**Nested / simpler Model**	**Fixed Effects added**		**Random Effects**		**Model fit**			**LRT Test against nested**	
					**Subjects**	**Items**	**AIC**	**BIC**	**LL**	**df**	**X2**
RE only	Null	-	-		Slope for RC type + relativizer	Slope for RC type + relativizer	34842	34924	-17407		
FE main effects	Main effects	Null	RC type + relativizer		Slope for RC type + relativizer	Slope for RC type + relativizer	34841	34936	-17405	2	4.05
FE two-way interactions	Two-way interaction	Main effects	RC type + relativizer + RCtype*relativizer		Slope for RC type + relativizer	Slope for RC type + relativizer	34843	34943	-17404	1	0.72

Table S4.10. Results of the linear mixed-effect model for quasi first-pass reading time at R5 in the contrast *Que* versus *El cual*. The top of the table reports estimates/beta values, standard error (SE), confidence intervals (95% CI), *t*-values and levels of significance for fixed effects; the bottom of the table reports variance and standard deviation (S.D.) for the random effects, as well as their correlation.

Fixed Effects					
	Est/Beta	SE	95% CI	t	p

Intercept	242.69	17.52	208.34	277.04	13.84	< .001
RC type	-8.68	9.66	-27.63	10.26	-0.89	1.00
Relativizer	-0.06	9.10	-17.91	17.77	-0.007	1.00
RC type*relativizer	-9.88	11.60	-32.62	12.85	-0.85	1.00
Random Effects						
				Variance	S.D.	Correlation
Participant (slope for RC type)				351.04	18.73	
Participant (slope for relativizer)				89.24	9.44	0.42
Items (slope for RC type)				528.24	22.98	
Items (slope for relativizer)				384.46	19.60	0.76

Table S4.11. Models' comparisons using Likelihood-ratio test (LRT) for second-pass duration at R5 in the contrast *Que* versus *El cual*. The asterisk in the last column represents a *p*-value below the significance level of 0.05.

Model's selection

Sampling Units	N total obs = 2688<br>N Subjects = 48; N items = 28										
**Model specification**	**Model name**	**Nested / simpler Model**	**Fixed Effects added**		**Random Effects**		**Model fit**			**LRT Test against nested**	
					**Subjects**	**Items**	**AIC**	**BIC**	**LL**	**df**	**X2**
RE only	Null	-	-		Slope for RC type + relativizer	Slope for RC type + relativizer	36359	36441	-18165		
FE main effects	Main effects	Null	RC type + relativizer		Slope for RC type + relativizer	Slope for RC type + relativizer	36360	36454	-18164	2	2.97
FE two-way interactions	Two-way interaction	Main effects	RC type + relativizer + RCtype*relativizer		Slope for RC type + relativizer	Slope for RC type + relativizer	36359	36459	-18162	1	3.01

Table S4.12. Results of the linear mixed-effect model for second-pass duration at R5 in the contrast *Que* versus *El cual*. The top of the table reports estimates/beta values, standard error (SE), confidence intervals (95% CI), *zt*values and levels of significance

for fixed effects; the bottom of the table reports variance and standard deviation (S.D.) for the random effects, as well as their correlation.

Fixed Effects						
	Est/Beta	SE	95% CI		t	p
Intercept	66.14	10.84	44.89	87.38	6.10	< .001
RC type	-28.88	12.33	-53.04	-4.70	-2.34	.05
Relativizer	-22.07	12.12	-45.82	1.69	-1.82	.13
RC type*relativizer	27.55	15.86	-3.54	58.63	1.73	.13
Random Effects						
				Variance	S.D.	Correlation
Participant (slope for RC type)				480.81	21.92	
Participant (slope for relativizer)				960.89	30.99	1.00
Items (slope for RC type)				454.34	21.31	
Items (slope for relativizer)				31.69	5.62	-1.00

Section E. Supplementary material of the eye-movement measures for the contrast *Que* vs. *Quien*

Table S1. Mean reading times by condition at R3, R4 and R5 for the contrast *Que* vs. *Quien*. Eye-movement measures are first-fixation rate (FFR), first fixation (FF), first-pass duration (FPD), first-pass regression (FPR), quasi first-pass reading time (QFP), second-pass duration (SPD). The numbers in parentheses indicate standard deviation.

Region	Measure	RESTRICTIVE		NON-RESTRICTIVE	
		QUE	*QUIEN*	*QUE*	*QUIEN*
R3	FFR (%)	0.92 (0.27)	0.92 (0.27)	0.95 (0.22)	0.92 (0.28)
	FF (ms)	264.76 (140.7)	252.32 (138.34)	276.79 (146.44)	271.33 (164.08)
	FPD (ms)	297.77 (173.88)	279.82 (159.73)	307.22 (178.94)	301.47 (193.14)
	FPR (%)	0.15 (0.36)	0.15 (0.36)	0.26 (0.44)	0.28 (0.45)
	QFP (ms)	308.31 (184.2)	296.6 (174.78)	324.61 (196.88)	319.35 (209.23)
	SPD (ms)	62.34 (186.4)	71.39 (217.75)	111.48 (286.31)	121.65 (290.77)
R4	FFR (%)	0.68 (0.47)	0.85 (0.36)	0.71 (0.46)	0.83 (0.38)
	FF (ms)	156.47 (125.74)	207.29 (117.24)	159.19 (121.07)	201.73 (118.45)
	FPD (ms)	168.65 (139.95)	226.33 (144.09)	167.85 (132.99)	211.48 (129.5)
	FPR (%)	0.07 (0.26)	0.06 (0.24)	0.06 (0.23)	0.05 (0.23)

	QFP (ms)	172.74 (146.33)	233.34 (153.24)	170.23 (136.41)	215.05 (135.7)
	SPD (ms)	25.29 (114.21)	33.03 (180.33)	26.04 (153.73)	21.53 (123.42)
R5	FFR (%)	0.87 (0.33)	0.87 (0.34)	0.84 (0.37)	0.86 (0.34)
	FF (ms)	191.36 (96.31)	193.22 (97.29)	181.84 (98.43)	188.29 (93.24)
	FPD (ms)	251.72 (162.43)	233.72 (149.3)	234.78 (166.98)	239.84 (166.74)
	FPR (%)	0.17 (0.37)	0.07 (0.25)	0.07 (0.26)	0.06 (0.24)
	QFP (ms)	280.07 (192.02)	246.09 (168.13)	243.69 (174.05)	248.18 (179.61)
	SPD (ms)	90.33 (267.82)	52.09 (252.16)	33.2 (164.11)	33.39 (170.29)

Section E.2. Models' reports for eye-movement measures at R3

Table S2.1. Models' comparisons using Likelihood-ratio test (LRT) for first fixation at R3 in the contrast *Que* versus *Quien*. The asterisk in the last column represents a *p*-value below the significance level of 0.05.

Model's selection

Sampling Units	N total obs = 2688<br>N Subjects = 48; N items = 28										
**Model specification**	**Model name**	**Nested / simpler Model**	**Fixed Effects added**		**Random Effects**		**Model fit**			**LRT Test against nested**	
					**Subjects**	**Items**	**AIC**	**BIC**	**LL**	**df**	**X2**
RE only	Null	-	-		Slope for RC type + relativizer	Slope for RC type + relativizer	34214	34297	-17093		
FE main effects	Main effects	Null	RC type + relativizer		Slope for RC type + relativizer	Slope for RC type + relativizer	34210	34304	-17089	2	8.31*
FE two-way interactions	Two-way interaction	Main effects	RC type + relativizer + RCtype*relativizer		Slope for RC type + relativizer	Slope for RC type + relativizer	34212	34312	-17089	1	0.44

Table S2.2. Results of the linear mixed-effect model for first fixation at R3 in the contrast *Que* versus *Quien*. The top of the table reports estimates/beta values, standard error (SE), confidence intervals (95% CI), *t*-values and levels of significance for fixed

effects; the bottom of the table reports variance and standard deviation (S.D.) for the random effects, as well as their correlation.

Fixed Effects						
	Est/Beta	SE	95% CI		t	p
Intercept	264.76	8.88	247.35	282.16	29.81	< .001
RC type	12.03	7.82	-3.29	27.37	1.53	.34
Relativizer	-12.43	7.88	-27.89	3.02	-1.57	.34
RC type*relativizer	6.97	10.47	-13.55	27.49	0.66	.50
Random Effects						
				Variance	S.D.	Correlation
Participant (slope for RC type)				3.058 e+02	17.48	
Participant (slope for relativizer)				6.597 e+01	8.12	-0.54
Items (slope for RC type)				1.236 e-03	0.03	
Items (slope for relativizer)				1.684 e+02	12.97	-1.00

Table S2.3. Models' comparisons using Likelihood-ratio test (LRT) for first-fixation rate at R3 in the contrast *Que* versus *Quien*. The asterisk in the last column represents a *p*-value below the significance level of 0.05.

Model's selection

Sampling Units	N total obs = 2688<br>N Subjects = 48; N items = 28										
**Model specification**	**Model name**	**Nested / simpler Model**	**Fixed Effects added**	**Random Effects**		**Model fit**			**LRT Test against nested**	
				**Subjects**	**Items**	**AIC**	**BIC**	**LL**	**df**	**X2**
RE only	Null	-	-	Slope for RC type + relativizer	Slope for RC type + relativizer	1350.6	1427.2	-662.30		
FE main effects	Main effects	Null	RC type + relativizer	Slope for RC type + relativizer	Slope for RC type + relativizer	1352.8	1441.3	-661.42	2	1.75
FE two-way interactions	Two-way interaction	Main effects	RC type + relativizer + RCtype*relativizer	Slope for RC type + relativizer	Slope for RC type + relativizer	1353.1	1447.4	-660.53	1	1.76

Table S2.4. Results of the linear mixed-effect model for first-fixation rate at R3 in the contrast *Que* versus *Quien*. The top of the table reports estimates/beta values, standard error (SE), confidence intervals (95% CI), *z*-values and levels of significance for fixed effects; the bottom of the table reports variance and standard deviation (S.D.) for the random effects, as well as their correlation.

Fixed Effects						
	Est/Beta	SE	95% CI		z	p
Intercept	28.4	0.23	2.37	3.30	11.92	< .001
RC type	0.47	0.29	-0.10	1.04	1.60	.32
Relativizer	-0.004	0.26	-0.53	0.52	-0.01	.98
RC type*relativizer	0.49	0.34	-1.15	0.17	-1.43	.32
Random Effects						
				Variance	S.D.	Correlation
Participant (slope for RC type)				0.13	0.37	
Participant (slope for relativizer)				0.12	0.34	-0.17
Items (slope for RC type)				0.09	0.30	
Items (slope for relativizer)				0.10	0.31	0.81

Table S2.5. Models' comparisons using Likelihood-ratio test (LRT) for first-pass duration at R3 in the contrast *Que* versus *Quien*. The asterisk in the last column represents a *p*-value below the significance level of 0.05.

Model's selection

Sampling Units	N total obs = 2688<br>N Subjects = 48; N items = 28											
**Model specification**	**Model name**	**Nested / simpler Model**	**Fixed Effects added**		**Random Effects**		**Model fit**			**LRT Test against nested**	
					**Subjects**	**Items**	**AIC**	**BIC**	**LL**	**df**	**X2**
RE only	Null	-	-		Slope for RC type + relativizer	Slope for RC type + relativizer	35138	35221	-17555		

FE main effects	Main effects	Null	RC type + relativizer		Slope for RC type + relativizer	Slope for RC type + relativizer	35136	35230	-17552	2	6.34*
FE two-way interactions	Two-way interaction	Main effects	RC type + relativizer + RCtype*relativizer		Slope for RC type + relativizer	Slope for RC type + relativizer	35137	35237	-17552	1	0.96

Table S2.6. Results of the linear mixed-effect model for first-pass duration at R3 in the contrast *Que* versus *Quien*. The top of the table reports estimates/beta values, standard error (SE), confidence intervals (95% CI), *t*-values and levels of significance for fixed effects; the bottom of the table reports variance and standard deviation (S.D.) for the random effects, as well as their correlation.

Fixed Effects						
	Est/Beta	SE	95% CI		t	p
Intercept	297.77	11.07	276.06	319.48	26.88	< .001
RC type	9.44	9.47	-9.11	28.01	0.99	.63
Relativizer	-17.95	9.53	-36.64	0.73	-1.88	.17
RC type*relativizer	12.20	12.40	-12.09	36.51	0.98	.63

Random Effects			
	Variance	S.D.	Correlation
Participant (slope for RC type)	6.180 e+02	24.85	
Participant (slope for relativizer)	1.933 e+02	13.90	-0.63
Items (slope for RC type)	4.737 e-04	0.02	
Items (slope for relativizer)	2.811 e +02	16.76	0.16

Table S2.7. Models' comparisons using Likelihood-ratio test (LRT) for first-pass regression at R3 in the contrast *Que* versus *Quien*. The asterisk in the last column represents a *p*-value below the significance level of 0.05.

Model's selection

Sampling Units	N total obs = 2688<br>N Subjects = 48; N items = 28					
**Model specification**	**Model name**	**Nested /**	**Fixed Effects added**		**Random Effects**	**Model fit**	**LRT Test against**

		simpler Model								nested	
					Subjects	Items	AIC	BIC	LL	df	X2
RE only	Null	-	-		Slope for RC type + relativizer	Slope for RC type + relativizer	2610.6	2687.2	-1292.3		
FE main effects	Main effects	Null	RC type + relativizer		Slope for RC type + relativizer	Slope for RC type + relativizer	2594.2	2682.7	-1282.1	2	20.32*
FE two-way interactions	Two-way interaction	Main effects	RC type + relativizer + RCtype*relativizer		Slope for RC type + relativizer	Slope for RC type + relativizer	2595.6	2689.9	-1281.8	1	0.64

Table S2.8. Results of the linear mixed-effect model for first-pass regression at R3 in the contrast *Que* versus *Quien*. The top of the table reports estimates/beta values, standard error (SE), confidence intervals (95% CI), *z*-values and levels of significance for fixed effects; the bottom of the table reports variance and standard deviation (S.D.) for the random effects, as well as their correlation.

Fixed Effects						
	Est/Beta	SE	95% CI		z	p
Intercept	-1.90	0.17	-2.25	-1.55	-10.78	< .001
RC type	0.65	0.18	0.30	1.01	3.64	< .001
Relativizer	-0.02	0.17	-0.36	0.31	-0.13	.89
RC type*relativizer	0.16	0.20	-0.24	0.58	0.81	.83
Random Effects						
				Variance	S.D.	Correlation
Participant (slope for RC type)				0.21	0.46	
Participant (slope for relativizer)				0.007	0.08	0.79
Items (slope for RC type)				0.09	0.30	
Items (slope for relativizer)				0.03	0.19	-0.98

Table S2.9. Models' comparisons using Likelihood-ratio test (LRT) for quasi first-pass reading time at R3 in the contrast *Que* versus *Quien*. The asterisk in the last column represents a *p*-value below the significance level of 0.05.

Model's selection

Sampling Units	N total obs = 2688<br>N Subjects = 48; N items = 28									
**Model specification**	**Model name**	**Nested / simpler Model**	**Fixed Effects added**	**Random Effects**		**Model fit**			**LRT Test against nested**	
				**Subjects**	**Items**	**AIC**	**BIC**	**LL**	**df**	**X2**
RE only	Null	-	-	Slope for RC type + relativizer	Slope for RC type + relativizer	35501	35584	-17737		
FE main effects	Main effects	Null	RC type + relativizer	Slope for RC type + relativizer	Slope for RC type + relativizer	35499	35593	-17734	2	6.238*
FE two-way interactions	Two-way interaction	Main effects	RC type + relativizer + RCtype*relativizer	Slope for RC type + relativizer	Slope for RC type + relativizer	35500	35600	-17733	1	0.99

Table S2.10. Results of the linear mixed-effect model for quasi first-pass reading time at R3 in the contrast *Que* versus *Quien*. The top of the table reports estimates/beta values, standard error (SE), confidence intervals (95% CI), *t*-values and levels of significance for fixed effects; the bottom of the table reports variance and standard deviation (S.D.) for the random effects, as well as their correlation.

Fixed Effects						
	Est/Beta	SE	95% CI		t	p
Intercept	308.31	12.37	284.06	332.56	24.91	< .001
RC type	16.29	10.12	-3.55	36.14	1.60	.32
Relativizer	-11.71	10.00	-31.31	7.88	-1.17	.48
RC type*relativizer	6.46	13.25	-19.51	32.43	0.48	.62

Random Effects			
	Variance	S.D.	Correlation
Participant (slope for RC type)	702.46	26.50	
Participant (slope for relativizer)	185.61	13.62	-0.19
Items (slope for RC type)	3.091	1.75	

Items (slope for relativizer)	233.49	15.28	-1.00

Table S2.11. Models' comparisons using Likelihood-ratio test (LRT) for second-pass duration at R3 in the contrast *Que* versus *Quien*. The asterisk in the last column represents a *p*-value below the significance level of 0.05.

Model's selection

Sampling Units	N total obs = 2688<br>N Subjects = 48; N items = 28										
**Model specification**	**Model name**	**Nested / simpler Model**	**Fixed Effects added**		**Random Effects**		**Model fit**			**LRT Test against nested**	
					**Subjects**	**Items**	**AIC**	**BIC**	**LL**	**df**	**X2**
RE only	Null	-	-		Slope for RC type + relativizer	Slope for RC type + relativizer	37181	37264	-18577		
FE main effects	Main effects	Null	RC type + relativizer		Slope for RC type + relativizer	Slope for RC type + relativizer	37178	37272	-18573	2	7.34*
FE two-way interactions	Two-way interaction	Main effects	RC type + relativizer + RCtype*relativizer		Slope for RC type + relativizer	Slope for RC type + relativizer	37180	37280	-18573	1	0.003

Table S2.12. Results of the linear mixed-effect model for second-pass duration at R3 in the contrast *Que* versus *Quien*. The top of the table reports estimates/beta values, standard error (SE), confidence intervals (95% CI), *t*-values and levels of significance for fixed effects; the bottom of the table reports variance and standard deviation (S.D.) for the random effects, as well as their correlation.

Fixed Effects						
	Est/Beta	SE	95% CI		t	p
Intercept	62.34	9.15	44.40	80.28	6.81	< .001
RC type	49.14	18.29	13.27	85.00	2.68	.02
Relativizer	9.05	14.84	-20.03	38.14	0.61	1.00
RC type*relativizer	1.11	18.30	-34.76	36.98	0.06	1.00

Random Effects			
	Variance	S.D.	Correlation
Participant (slope for RC type)	5451.0	73.83	
Participant (slope for relativizer)	1413.3	37.59	1.00
Items (slope for RC type)	1505.6	38.80	
Items (slope for relativizer)	653.1	25.55	-0.16

Section E.3. Models' reports for eye-movement measures at R4

Table S3.1. Models' comparisons using Likelihood-ratio test (LRT) for first fixation at R4 in the contrast *Que* versus *Quien*. The asterisk in the last column represents a *p*-value below the significance level of 0.05.

Model's selection

Sampling Units	N total obs = 2688<br>N Subjects = 48; N items = 28										
**Model specification**	**Model name**	**Nested / simpler Model**	**Fixed Effects added**		**Random Effects**		**Model fit**			**LRT Test against nested**	
					**Subjects**	**Items**	**AIC**	**BIC**	**LL**	**df**	**X2**
RE only	Null	-	-		Slope for RC type + relativizer	Slope for RC type + relativizer	32923	33006	-16448		
FE main effects	Main effects	Null	RC type + relativizer		Slope for RC type + relativizer	Slope for RC type + relativizer	32881	32975	-16425	2	45.98*
FE two-way interactions	Two-way interaction	Main effects	RC type + relativizer + RCtype*relativizer		Slope for RC type + relativizer	Slope for RC type + relativizer	32882	32982	-16424	1	1.03

Table S3.2. Results of the linear mixed-effect model for first fixation at R4 in the contrast *Que* versus *Quien*. The top of the table reports estimates/beta values, standard error (SE), confidence intervals (95% CI), *t*-values and levels of significance for fixed

effects; the bottom of the table reports variance and standard deviation (S.D.) for the random effects, as well as their correlation.

Fixed Effects						
	Est/Beta	SE	95% CI		t	p
Intercept	156.47	10.29	136.28	176.65	15.19	< .001
RC type	2.71	6.61	-10.24	15.68	0.41	.68
Relativizer	50.81	6.51	38.05	63.58	7.80	< .001
RC type*relativizer	-8.27	8.11	-24.18	7.63	-1.01	.61
Random Effects						
				Variance	S.D.	Correlation
Participant (slope for RC type)				243.67	15.61	
Participant (slope for relativizer)				391.05	19.77	-0.03
Items (slope for RC type)				160.17	12.65	
Items (slope for relativizer)				37.36	6.11	-0.33

Table S3.3. Models' comparisons using Likelihood-ratio test (LRT) for first-fixation rate at R4 in the contrast *Que* versus *Quien*. The asterisk in the last column represents a *p*-value below the significance level of 0.05.

Model's selection

Sampling Units	N total obs = 2688<br>N Subjects = 48; N items = 28											
**Model specification**	**Model name**	**Nested / simpler Model**	**Fixed Effects added**		**Random Effects**		**Model fit**			**LRT Test against nested**	
					**Subjects**	**Items**	**AIC**	**BIC**	**LL**	**df**	**X2**
RE only	Null	-	-		Slope for RC type + relativizer	Slope for RC type + relativizer	2592.9	2669.6	-1283.5		
FE main effects	Main effects	Null	RC type + relativizer		Slope for RC type + relativizer	Slope for RC type + relativizer	2564.3	2652.8	-1267.2	2	32.62*

FE two-way interactions	Two-way interaction	Main effects	RC type + relativizer + RCtype*relativizer		Slope for RC type + relativizer	Slope for RC type + relativizer	2563.8	2658.2	-1265.9	1	2.49

Table S3.4. Results of the linear mixed-effect model for first-fixation rate at R4 in the contrast *Que* versus *Quien*. The top of the table reports estimates/beta values, standard error (SE), confidence intervals (95% CI), *z*-values and levels of significance for fixed effects; the bottom of the table reports variance and standard deviation (S.D.) for the random effects, as well as their correlation.

Fixed Effects						
	Est/Beta	SE	95% CI		z	p
Intercept	0.95	0.20	0.55	1.36	4.66	< .001
RC type	0.14	0.14	-0.14	0.42	0.97	.33
Relativizer	1.15	0.17	0.79	1.50	6.40	< .001
RC type*relativizer	-0.33	0.21	-0.75	0.07	-1.60	.21

Random Effects			
	Variance	S.D.	Correlation
Participant (slope for RC type)	0.07	0.26	
Participant (slope for relativizer)	0.25	0.50	1.00
Items (slope for RC type)	0.01	0.11	
Items (slope for relativizer)	0.002	0.04	-1.00

Table S3.5. Models' comparisons using Likelihood-ratio test (LRT) for first-pass duration at R4 in the contrast *Que* versus *Quien*. The asterisk in the last column represents a *p*-value below the significance level of 0.05.

Model's selection

Sampling Units	N total obs = 2688<br>N Subjects = 48; N items = 28

**Model specification**	**Model name**	**Nested / simpler Model**	**Fixed Effects added**		**Random Effects**		**Model fit**			**LRT Test against nested**	
					**Subjects**	**Items**	**AIC**	**BIC**	**LL**	**df**	**X2**

RE only	Null	-	-		Slope for RC type + relativizer	Slope for RC type + relativizer	33576	33659	-16774		
FE main effects	Main effects	Null	RC type + relativizer		Slope for RC type + relativizer	Slope for RC type + relativizer	33536	33631	-16752	2	43.77*
FE two-way interactions	Two-way interaction	Main effects	RC type + relativizer + RCtype*relativizer		Slope for RC type + relativizer	Slope for RC type + relativizer	33536	33636	-16751	1	2.34

Table S3.6. Results of the linear mixed-effect model for first-pass duration at R4 in the contrast *Que* versus *Quien*. The top of the table reports estimates/beta values, standard error (SE), confidence intervals (95% CI), *t*-values and levels of significance for fixed effects; the bottom of the table reports variance and standard deviation (S.D.) for the random effects, as well as their correlation.

Fixed Effects						
	Est/Beta	SE	95% CI		t	p
Intercept	168.65	11.71	145.69	191.61	14.39	< .001
RC type	-0.79	7.41	-15.33	13.73	-0.10	.91
Relativizer	57.68	7.12	43.70	71.65	8.09	< .001
RC type*relativizer	-14.05	9.17	-32.03	3.91	-1.53	.25

Random Effects			
	Variance	S.D.	Correlation
Participant (slope for RC type)	220.46	14.84	
Participant (slope for relativizer)	301.90	17.37	0.15
Items (slope for RC type)	233.08	15.26	
Items (slope for relativizer)	69.44	8.33	-0.64

Table S3.7. Models' comparisons using Likelihood-ratio test (LRT) for first-pass regression at R4 in the contrast *Que* versus *Quien*. The asterisk in the last column represents a *p*-value below the significance level of 0.05.

Model's selection

Sampling Units	N total obs = 2688

	N Subjects = 48; N items = 28											
**Model specification**	**Model name**	**Nested / simpler Model**	**Fixed Effects added**		**Random Effects**		**Model fit**			**LRT Test against nested**	
					**Subjects**	**Items**	**AIC**	**BIC**	**LL**	**df**	**X2**
RE only	Null	-	-		Slope for RC type + relativizer	Slope for RC type + relativizer	1215.7	1292.3	-594.85		
FE main effects	Main effects	Null	RC type + relativizer		Slope for RC type + relativizer	Slope for RC type + relativizer	1218.6	1307.0	-594.28	2	1.12
FE two-way interactions	Two-way interaction	Main effects	RC type + relativizer + RCtype*relati vizer		Slope for RC type + relativizer	Slope for RC type + relativizer	1220.2	1314.6	-594.13	1	0.31

Table S3.8. Results of the linear mixed-effect model for first-pass regression at R4 in the contrast *Que* versus *Quien*. The top of the table reports estimates/beta values, standard error (SE), confidence intervals (95% CI), *z*-values and levels of significance for fixed effects; the bottom of the table reports variance and standard deviation (S.D.) for the random effects, as well as their correlation.

Fixed Effects						
	Est/Beta	SE	95% CI		z	p
Intercept	-2.87	0.23	-3.33	-2.41	-12.21	< .001
RC type	-0.35	0.31	-0.96	0.25	-1.13	.77
Relativizer	-0.14	0.30	-0.73	0.44	-0.47	1.00
RC type*relativizer	0.21	0.38	-0.54	0.97	0.56	1.00

Random Effects			
	Variance	S.D.	Correlation
Participant (slope for RC type)	0.15	0.39	
Participant (slope for relativizer)	0.12	0.34	-0.99
Items (slope for RC type)	0.15	0.39	
Items (slope for relativizer)	0.15	0.39	-0.60

Table S3.9. Models' comparisons using Likelihood-ratio test (LRT) for quasi first-pass reading time at R4 in the contrast *Que* versus *Quien*. The asterisk in the last column represents a *p*-value below the significance level of 0.05.

Model's selection

Sampling Units	N total obs = 2688<br>N Subjects = 48; N items = 28										
**Model specification**	**Model name**	**Nested / simpler Model**	**Fixed Effects added**		**Random Effects**		**Model fit**			**LRT Test against nested**	
					**Subjects**	**Items**	**AIC**	**BIC**	**LL**	**df**	**X2**
RE only	Null	-	-		Slope for RC type + relativizer	Slope for RC type + relativizer	33843	33926	-16908		
FE main effects	Main effects	Null	RC type + relativizer		Slope for RC type + relativizer	Slope for RC type + relativizer	33808	33902	-16888	2	39.28*
FE two-way interactions	Two-way interaction	Main effects	RC type + relativizer + RCtype*relati vizer		Slope for RC type + relativizer	Slope for RC type + relativizer	33807	33907	-16887	1	2.67

Table S3.10. Results of the linear mixed-effect model for quasi first-pass reading time at R4 in the contrast *Que* versus *Quien*. The top of the table reports estimates/beta values, standard error (SE), confidence intervals (95% CI), *t*-values and levels of significance for fixed effects; the bottom of the table reports variance and standard deviation (S.D.) for the random effects, as well as their correlation.

Fixed Effects						
	Est/Beta	SE	95% CI		t	p
Intercept	172.74	12.10	149.00	196.47	14.26	< .001
RC type	-2.50	7.94	-18.06	13.05	-0.31	.75
Relativizer	60.60	7.73	45.44	75.75	7.83	< .001
RC type*relativizer	-15.77	9.64	-34.67	3.12	-1.63	.20
Random Effects						

	Variance	S.D.	Correlation
Participant (slope for RC type)	271.9	16.49	
Participant (slope for relativizer)	321.3	17.92	0.02
Items (slope for RC type)	304.8	17.46	
Items (slope for relativizer)	185.5	13.62	-0.56

Table S3.11. Models' comparisons using Likelihood-ratio test (LRT) for second-pass duration at R4 in the contrast *Que* versus *Quien*. The asterisk in the last column represents a *p*-value below the significance level of 0.05.

Model's selection

Sampling Units	N total obs = 2688<br>N Subjects = 48; N items = 28										
**Model specification**	**Model name**	**Nested / simpler Model**	**Fixed Effects added**		**Random Effects**		**Model fit**			**LRT Test against nested**	
					**Subjects**	**Items**	**AIC**	**BIC**	**LL**	**df**	**X2**
RE only	Null	-	-		Slope for RC type + relativizer	Slope for RC type + relativizer	34389	34472	-17181		
FE main effects	Main effects	Null	RC type + relativizer		Slope for RC type + relativizer	Slope for RC type + relativizer	34393	34487	-17180	2	0.55
FE two-way interactions	Two-way interaction	Main effects	RC type + relativizer + RCtype*relativizer		Slope for RC type + relativizer	Slope for RC type + relativizer	34393	34494	-17180	1	1.24

Table S3.12. Results of the linear mixed-effect model for second-pass duration at R4 in the contrast *Que* versus *Quien*. The top of the table reports estimates/beta values, standard error (SE), confidence intervals (95% CI), *t*-values and levels of significance for fixed effects; the bottom of the table reports variance and standard deviation (S.D.) for the random effects, as well as their correlation.

Fixed Effects					
	Est/Beta	SE	95% CI	t	p

Intercept	25.29	6.25	13.03	37.55	4.04	< .001
RC type	0.74	9.21	-17.31	18.80	0.08	.93
Relativizer	7.73	9.76	-11.40	26.87	0.79	.85
RC type*relativizer	-12.24	10.99	-33.78	9.29	-1.11	.79
Random Effects						
				Variance	S.D.	Correlation
Participant (slope for RC type)				274.88	16.58	
Participant (slope for relativizer)				514.54	22.68	-0.95
Items (slope for RC type)				525.08	22.91	
Items (slope for relativizer)				679.00	26.05	-1.00

Section E.4. Models' reports for eye-movement measures at R5

Table S4.1. Models' comparisons using Likelihood-ratio test (LRT) for first fixation at R5 in the contrast *Que* versus *Quien*. The asterisk in the last column represents a *p*-value below the significance level of 0.05.

Model's selection

Sampling Units	N total obs = 2688<br>N Subjects = 48; N items = 28										
**Model specification**	**Model name**	**Nested / simpler Model**	**Fixed Effects added**		**Random Effects**		**Model fit**			**LRT Test against nested**	
					**Subjects**	**Items**	**AIC**	**BIC**	**LL**	**df**	**X2**
RE only	Null	-	-		Slope for RC type + relativizer	Slope for RC type + relativizer	31718	31800	-15845		
FE main effects	Main effects	Null	RC type + relativizer		Slope for RC type + relativizer	Slope for RC type + relativizer	31715	31810	-15842	2	6.30*
FE two-way interactions	Two-way interaction	Main effects	RC type + relativizer + RCtype*relativizer		Slope for RC type + relativizer	Slope for RC type + relativizer	31717	31817	-15842	1	0.48

Table S4.2. Results of the linear mixed-effect model for first fixation at R5 in the contrast *Que* versus *Quien*. The top of the table reports estimates/beta values, standard error (SE), confidence intervals (95% CI), *t*-values and levels of significance for fixed effects; the bottom of the table reports variance and standard deviation (S.D.) for the random effects, as well as their correlation.

Fixed Effects						
	Est/Beta	SE	95% CI		t	p
Intercept	191.36	7.25	177.13	205.58	26.36	< .001
RC type	-9.51	4.88	-19.09	0.06	-1.94	.15
Relativizer	1.86	5.19	-8.31	12.04	0.35	.96
RC type*relativizer	4.58	6.55	-8.26	17.43	0.69	.96
Random Effects						
				Variance	S.D.	Correlation
Participant (slope for RC type)				113.06	10.63	
Participant (slope for relativizer)				263.01	16.21	0.96
Items (slope for RC type)				1.28	1.13	
Items (slope for relativizer)				0.53	0.72	-1.00

Table S4.3. Models' comparisons using Likelihood-ratio test (LRT) for first-fixation rate at R5 in the contrast *Que* versus *Quien*. The asterisk in the last column represents a *p*-value below the significance level of 0.05.

Model's selection

Sampling Units	N total obs = 2688<br>N Subjects = 48; N items = 28										
**Model specification**	**Model name**	**Nested / simpler Model**	**Fixed Effects added**		**Random Effects**		**Model fit**			**LRT Test against nested**	
					**Subjects**	**Items**	**AIC**	**BIC**	**LL**	**df**	**X2**
RE only	Null	-	-		Slope for RC type + relativizer	Slope for RC type + relativizer	1960.0	2036.7	-967.00		
FE main effects	Main effects	Null	RC type + relativizer		Slope for RC type + relativizer	Slope for RC type + relativizer	1963.3	2051.8	-966.67	2	0.67

Model specification	Model name	Nested / simpler Model	Fixed Effects added		Subjects	Items	AIC	BIC	LL	df	X2
FE two-way interactions	Two-way interaction	Main effects	RC type + relativizer + RCtype*relativizer		Slope for RC type + relativizer	Slope for RC type + relativizer	1962.8	2057.1	-965.39	1	2.56

Table S4.4. Results of the linear mixed-effect model for first-fixation rate at R5 in the contrast *Que* versus *Quien*. The top of the table reports estimates/beta values, standard error (SE), confidence intervals (95% CI), *z*-values and levels of significance for fixed effects; the bottom of the table reports variance and standard deviation (S.D.) for the random effects, as well as their correlation.

Fixed Effects						
	Est/Beta	SE	95% CI		z	p
Intercept	2.34	0.21	1.91	2.76	19.78	< .001
RC type	-0.32	0.19	-0.70	0.05	-1.67	.28
Relativizer	-0.19	0.19	-0.58	0.19	-0.96	.33
RC type*relativizer	0.39	0.24	-0.08	0.87	1.62	.28

Random Effects			
	Variance	S.D.	Correlation
Participant (slope for RC type)	0.09	0.31	
Participant (slope for relativizer)	0.13	0.37	0.72
Items (slope for RC type)	0.0008	0.02	
Items (slope for relativizer)	0.003	0.05	1.00

Table S4.5. Models' comparisons using Likelihood-ratio test (LRT) for first-pass duration at R5 in the contrast *Que* versus *Quien*. The asterisk in the last column represents a *p*-value below the significance level of 0.05.

Model's selection

Sampling Units	N total obs = 2688<br>N Subjects = 48; N items = 28										
**Model specification**	**Model name**	**Nested / simpler Model**	**Fixed Effects added**		**Random Effects**		**Model fit**			**LRT Test against nested**	
					**Subjects**	**Items**	**AIC**	**BIC**	**LL**	**df**	**X2**

RE only	Null	-	-		Slope for RC type + relativizer	Slope for RC type + relativizer	34390	34473	-17181		
FE main effects	Main effects	Null	RC type + relativizer		Slope for RC type + relativizer	Slope for RC type + relativizer	34393	34488	-17181	2	1.21
FE two-way interactions	Two-way interaction	Main effects	RC type + relativizer + RCtype*relativizer		Slope for RC type + relativizer	Slope for RC type + relativizer	34365	34465	-17166	1	30.06*

Table S4.6. Results of the linear mixed-effect model for first-pass duration at R5 in the contrast *Que* versus *Quien*. The top of the table reports estimates/beta values, standard error (SE), confidence intervals (95% CI), *t*-values and levels of significance for fixed effects; the bottom of the table reports variance and standard deviation (S.D.) for the random effects, as well as their correlation.

Fixed Effects						
	Est/Beta	SE	95% CI		t	p
Intercept	251.72	12.55	227.12	276.33	20.05	< .001
RC type	-16.93	8.28	-33.18	-0.69	-2.04	.09
Relativizer	-18.00	8.50	-34.66	-1.33	-2.11	.09
RC type*relativizer	23.05	10.67	2.13	43.97	2.16	.09
Random Effects						
				Variance	S.D.	Correlation
Participant (slope for RC type)				457.48	21.38	
Participant (slope for relativizer)				557.16	23.60	0.72
Items (slope for RC type)				61.55	7.84	
Items (slope for relativizer)				103.22	10.16	-1.00

Table S4.7. Models' comparisons using Likelihood-ratio test (LRT) for first-pass regression at R5 in the contrast *Que* versus *Quien*. The asterisk in the last column represents a *p*-value below the significance level of 0.05.

Model's selection

Sampling Units	N total obs = 2688

	N Subjects = 48; N items = 28										
**Model specification**	**Model name**	**Nested / simpler Model**	**Fixed Effects added**		**Random Effects**		**Model fit**			**LRT Test against nested**	
					**Subjects**	**Items**	**AIC**	**BIC**	**LL**	**df**	**X2**
RE only	Null	-	-		Slope for RC type + relativizer	Slope for RC type + relativizer	1576.2	1652.8	-775.10		
FE main effects	Main effects	Null	RC type + relativizer		Slope for RC type + relativizer	Slope for RC type + relativizer	1561.6	1650.0	-765.79	2	18.62*
FE two-way interactions	Two-way interaction	Main effects	RC type + relativizer + RCtype*relativizer		Slope for RC type + relativizer	Slope for RC type + relativizer	1551.5	1645.8	-759.72	1	12.12*

Table S4.8. Results of the linear mixed-effect model for first-pass regression at R5 in the contrast *Que* versus *Quien*. The top of the table reports estimates/beta values, standard error (SE), confidence intervals (95% CI), *z*-values and levels of significance for fixed effects; the bottom of the table reports variance and standard deviation (S.D.) for the random effects, as well as their correlation.

Fixed Effects						
	Est/Beta	SE	95% CI		z	p
Intercept	-1.85	0.18	-2.20	-1.49	-10.28	< .001
RC type	-1.20	0.28	-1.75	-0.65	-4.29	< .001
Relativizer	-1.13	0.26	-1.64	-0.62	-4.33	< .001
RC type*relativizer	1.22	0.36	0.51	1.93	3.38	< .001

Random Effects			
	Variance	S.D.	Correlation
Participant (slope for RC type)	1.116 e-01	0.33	
Participant (slope for relativizer)	2.177 e-01	0.46	-0.36
Items (slope for RC type)	3.589 e-01	0.59	
Items (slope for relativizer)	1.736 e-01	0.41	-0.99

Table S4.9. Models' comparisons using Likelihood-ratio test (LRT) for quasi first-pass reading time at R5 in the contrast *Que* versus *Quien*. The asterisk in the last column represents a *p*-value below the significance level of 0.05.

Model's selection

Sampling Units	N total obs = 2688<br>N Subjects = 48; N items = 28									
**Model specification**	**Model name**	**Nested / simpler Model**	**Fixed Effects added**	**Random Effects**		**Model fit**			**LRT Test against nested**	
				**Subjects**	**Items**	**AIC**	**BIC**	**LL**	**df**	**X2**
RE only	Null	-	-	Slope for RC type + relativizer	Slope for RC type + relativizer	34922	35005	-17447		
FE main effects	Main effects	Null	RC type + relativizer	Slope for RC type + relativizer	Slope for RC type + relativizer	34919	35014	-17444	2	7.15*
FE two-way interactions	Two-way interaction	Main effects	RC type + relativizer + RCtype*relativizer	Slope for RC type + relativizer	Slope for RC type + relativizer	34911	35011	-17438	1	10.52*

Table S4.10. Results of the linear mixed-effect model for quasi first-pass reading time at R5 in the contrast *Que* versus *Quien*. The top of the table reports estimates/beta values, standard error (SE), confidence intervals (95% CI), *t*-values and levels of significance for fixed effects; the bottom of the table reports variance and standard deviation (S.D.) for the random effects, as well as their correlation.

Fixed Effects						
	Est/Beta	SE	95% CI		t	p
Intercept	280.07	14.05	252.53	307.60	19.93	< .001
RC type	-36.37	10.02	-56.02	-16.73	-3.62	< .001
Relativizer	-33.97	9.10	-51.81	-16.12	-3.73	< .001
RC type*relativizer	38.46	11.84	15.24	61. 68	3.24	.001
Random Effects						

	Variance	S.D.	Correlation
Participant (slope for RC type)	129.6	11.39	
Participant (slope for relativizer)	519.9	22.80	0.56
Items (slope for RC type)	772.2	27.79	
Items (slope for relativizer)	53.0	7.28	1.00

Table S4.11. Models' comparisons using Likelihood-ratio test (LRT) for second-pass duration at R5 in the contrast *Que* versus *Quien*. The asterisk in the last column represents a *p*-value below the significance level of 0.05.

Model's selection

Sampling Units	N total obs = 2688<br>N Subjects = 48; N items = 28										
**Model specification**	**Model name**	**Nested / simpler Model**	**Fixed Effects added**		**Random Effects**		**Model fit**			**LRT Test against nested**	
					**Subjects**	**Items**	**AIC**	**BIC**	**LL**	**df**	**X2**
RE only	Null	-	-		Slope for RC type + relativizer	Slope for RC type + relativizer	36597	36680	-18285		
FE main effects	Main effects	Null	RC type + relativizer		Slope for RC type + relativizer	Slope for RC type + relativizer	36619	36714	-18294	2	0
FE two-way interactions	Two-way interaction	Main effects	RC type + relativizer + RCtype*relativizer		Slope for RC type + relativizer	Slope for RC type + relativizer	36578	36679	-18272	1	42.90*

Table S4.12. Results of the linear mixed-effect model for second-pass duration at R5 in the contrast *Que* versus *Quien*. The top of the table reports estimates/beta values, standard error (SE), confidence intervals (95% CI), *t*-values and levels of significance for fixed effects; the bottom of the table reports variance and standard deviation (S.D.) for the random effects, as well as their correlation.

Fixed Effects					
	Est/Beta	SE	95% CI	t	p

Intercept	90.34	11.83	67.14 113.52	7.63	< .001
RC type	-57.13	12.87	-82.35 -31.91	-4.44	< .001
Relativizer	-38.24	12.01	-61.78 -14.69	-3.18	.002
RC type*relativizer	38.43	16.53	6.03 70.83	2.32	.02
Random Effects					
			Variance	S.D.	Correlation
Participant (slope for RC type)			709.9	26.64	
Participant (slope for relativizer)			106.9	10.34	1.00
Items (slope for RC type)			396.5	19.91	
Items (slope for relativizer)			152.0	12.33	-0.85

Section F. Supplementary material of the eye-movement measures for the contrast *Que* vs. *Donde*

Table S1. Mean reading times by condition at R3, R4 and R5 for the contrast *Que* vs. *Donde*. Eye-movement measures are first-fixation rate (FFR), first fixation (FF), first-pass duration (FPD), first-pass regression (FPR), quasi first-pass reading time (QFP), second-pass duration (SPD). The numbers in parentheses indicate standard deviation.

Region	Measure	RESTRICTIVE		NON-RESTRICTIVE	
		QUE	*DONDE*	*QUE*	*DONDE*
R3	FFR (%)	0.92 (0.28)	0.92 (0.27)	0.92 (0.27)	0.93 (0.26)
	FF (ms)	248.72 (126.44)	239.18 (117.41)	247.52 (134.72)	259.01 (146.1)
	FPD (ms)	287.05 (176.41)	275.45 (167.98)	284.18 (194.26)	299.75 (203.85)
	FPR (%)	0.14 (0.35)	0.15 (0.36)	0.23 (0.42)	0.23 (0.42)
	QFP (ms)	304.23 (195.54)	293.65 (186.1)	306.77 (214.99)	318.63 (227.2)
	SPD (ms)	69.9 (229.81)	65.36(191.99)	103.12 (266.41)	98.56 (250.15)
R4	FFR (%)	0.79 (0.41)	0.84 (0.36)	0.82 (0.38)	0.82 (0.38)
	FF (ms)	195.83 (135.44)	190.21 (104.41)	206.78 (138.08)	183.45 (102.96)
	FPD (ms)	205.57 (144.95)	203.56 (122.54)	213.47 (146.32)	198.2 (125.81)
	FPR (%)	0.10 (0.30)	0.06 (0.24)	0.09 (0.29)	0.07 (0.26)
	QFP (ms)	210.14 (151.37)	212.83 (134.33)	218.8 (154.56)	206.77 (137.38)
	SPD (ms)	36.99 (150.57)	30.47 (138.78)	41.53 (173.3)	42.41 (275.21)
R5	FFR (%)	0.84 (0.36)	0.86 (0.34)	0.83 (0.38)	0.84 (0.37)
	FF (ms)	189.78 (103)	188.5 (93.45)	181.05 (98.94)	186.98 (100.18)
	FPD (ms)	245.1 (186.69)	223.2 (141.15)	238.79 (179.11)	233.44 (166.31)
	FPR (%)	0.11 (0.31)	0.04 (0.20)	0.09 (0.29)	0.04 (0.20)
	QFP (ms)	262.04 (204.22)	228.2 (145.37)	251.2 (191.65)	237.97 (168.83)
	SPD (ms)	59.32 (239.32)	27.83 (193.06)	60.7 (241.03)	26.92 (154.92)

Section F.2. Models' reports for eye-movement measures at R3

Table S2.1. Models' comparisons using Likelihood-ratio test (LRT) for first fixation at R3 in the contrast *Que* versus *Donde*. The asterisk in the last column represents a *p*-value below the significance level of 0.05.

Model's selection

Sampling Units	N total obs = 2688<br>N Subjects = 48; N items = 28										
**Model specification**	**Model name**	**Nested / simpler Model**	**Fixed Effects added**		**Random Effects**		**Model fit**			**LRT Test against nested**	
					**Subjects**	**Items**	**AIC**	**BIC**	**LL**	**df**	**X2**
RE only	Null	-	-		Slope for RC type + relativizer	Slope for RC type + relativizer	33603	33685	-16787		
FE main effects	Main effects	Null	RC type + relativizer		Slope for RC type + relativizer	Slope for RC type + relativizer	33604	33699	-16786	2	2.28
FE two-way interactions	Two-way interaction	Main effects	RC type + relativizer + RCtype*relativizer		Slope for RC type + relativizer	Slope for RC type + relativizer	33601	33702	-16784	1	4.99*

Table S2.2. Results of the linear mixed-effect model for first fixation at R3 in the contrast *Que* versus *Donde*. The top of the table reports estimates/beta values, standard error (SE), confidence intervals (95% CI), *t*-values and levels of significance for fixed effects; the bottom of the table reports variance and standard deviation (S.D.) for the random effects, as well as their correlation.

Fixed Effects						
	Est/Beta	SE	95% CI		t	p
Intercept	239.18	8.07	223.35	255.01	29.61	< .001
RC type	19.76	7.65	4.75	34.77	2.58	.02
Relativizer	9.54	6.79	-3.77	22.85	1.40	.16
RC type*relativizer	-21.00	9.39	-39.41	-2.58	-2.23	.05

Random Effects			
	Variance	S.D.	Correlation
Participant (slope for RC type)	583.22	24.15	
Participant (slope for relativizer)	34.48	5.87	-0.71
Items (slope for RC type)	66.37	8.14	
Items (slope for relativizer)	38.60	6.21	-1.00

Table S2.3. Models' comparisons using Likelihood-ratio test (LRT) for first-fixation rate at R3 in the contrast *Que* versus *Donde*. The asterisk in the last column represents a *p*-value below the significance level of 0.05.

Model's selection

Sampling Units	N total obs = 2688<br>N Subjects = 48; N items = 28										
**Model specification**	**Model name**	**Nested / simpler Model**	**Fixed Effects added**		**Random Effects**		**Model fit**			**LRT Test against nested**	
					**Subjects**	**Items**	**AIC**	**BIC**	**LL**	**df**	**X2**
RE only	Null	-	-		Slope for RC type + relativizer	Slope for RC type + relativizer	1446.8	1523.4	-710.39		
FE main effects	Main effects	Null	RC type + relativizer		Slope for RC type + relativizer	Slope for RC type + relativizer	1450.7	1539.1	-710.33	2	0.11
FE two-way interactions	Two-way interaction	Main effects	RC type + relativizer + RCtype*relativizer		Slope for RC type + relativizer	Slope for RC type + relativizer	1452.5	1546.9	-710.27	1	0.12

Table S2.4. Results of the linear mixed-effect model for first-fixation rate at R3 in the contrast *Que* versus *Donde*. The top of the table reports estimates/beta values, standard error (SE), confidence intervals (95% CI), *z*-values and levels of significance for fixed effects; the bottom of the table reports variance and standard deviation (S.D.) for the random effects, as well as their correlation.

Fixed Effects						
	Est/Beta	SE	95% CI		z	p
Intercept	2.69	0.19	2.30	3.08	13.62	< .001
RC type	0.11	0.25	-0.38	0.60	0.45	1.00
Relativizer	0.08	0.24	-0.39	0.56	0.34	1.00
RC type*relativizer	-0.10	0.30	-0.69	0.47	-0.36	1.00
Random Effects						
				Variance	S.D.	Correlation
Participant (slope for RC type)				0.01	0.10	
Participant (slope for relativizer)				0.01	0.11	0.99
Items (slope for RC type)				0.19	0.44	
Items (slope for relativizer)				0.15	0.39	-0.44

Table S2.5. Models' comparisons using Likelihood-ratio test (LRT) for first-pass duration at R3 in the contrast *Que* versus *Donde*. The asterisk in the last column represents a *p*-value below the significance level of 0.05.

Model's selection

Sampling Units	N total obs = 2688<br>N Subjects = 48; N items = 28											
**Model specification**	**Model name**	**Nested / simpler Model**	**Fixed Effects added**		**Random Effects**		**Model fit**			**LRT Test against nested**	
					**Subjects**	**Items**	**AIC**	**BIC**	**LL**	**df**	**X2**
RE only	Null	-	-		Slope for RC type + relativizer	Slope for RC type + relativizer	35356	35438	-17664		
FE main effects	Main effects	Null	RC type + relativizer		Slope for RC type + relativizer	Slope for RC type + relativizer	35358	35452	-17663	2	1.56
FE two-way interactions	Two-way interaction	Main effects	RC type + relativizer + RCtype*relativizer		Slope for RC type + relativizer	Slope for RC type + relativizer	35356	35457	-17661	1	3.62

Table S2.6. Results of the linear mixed-effect model for first-pass duration at R3 in the contrast *Que* versus *Donde*. The top of the table reports estimates/beta values, standard error (SE), confidence intervals (95% CI), *t*-values and levels of significance for fixed effects; the bottom of the table reports variance and standard deviation (S.D.) for the random effects, as well as their correlation.

Fixed Effects						
	Est/Beta	SE	95% CI		t	p
Intercept	275.45	13.24	249.48	301.41	20.79	< .001
RC type	24.26	10.92	2.85	45.67	2.22	.07
Relativizer	11.60	9.16	-6.36	29.57	1.26	.20
RC type*relativizer	-27.22	12.96	-52.63	-1.81	-2.10	.07

Random Effects			
	Variance	S.D.	Correlation
Participant (slope for RC type)	780.91	27.94	
Participant (slope for relativizer)	1.43	1.19	0.45
Items (slope for RC type)	532.01	23.06	
Items (slope for relativizer)	0.40	0.63	-0.70

Table S2.7. Models' comparisons using Likelihood-ratio test (LRT) for first-pass regression at R3 in the contrast *Que* versus *Donde*. The asterisk in the last column represents a *p*-value below the significance level of 0.05.

Model's selection

Sampling Units	N total obs = 2688<br>N Subjects = 48; N items = 28									
**Model specification**	**Model name**	**Nested / simpler Model**	**Fixed Effects added**	**Random Effects**		**Model fit**			**LRT Test against nested**	
				**Subjects**	**Items**	**AIC**	**BIC**	**LL**	**df**	**X2**
RE only	Null	-	-	Slope for RC type + relativizer	Slope for RC type + relativizer	2461.2	2537.9	-1217.6		
FE main effects	Main effects	Null	RC type + relativizer	Slope for RC type + relativizer	Slope for RC type + relativizer	2447.4	2535.9	-1208.7	2	17.78*

FE two-way interactions	Two-way interaction	Main effects	RC type + relativizer + RCtype*relativizer		Slope for RC type + relativizer	Slope for RC type + relativizer	2449.4	2543.7	-1208.7	1	0.03

Table S2.8. Results of the linear mixed-effect model for first-pass regression at R3 in the contrast *Que* versus *Donde*. The top of the table reports estimates/beta values, standard error (SE), confidence intervals (95% CI), *z*-values and levels of significance for fixed effects; the bottom of the table reports variance and standard deviation (S.D.) for the random effects, as well as their correlation.

Fixed Effects						
	Est/Beta	SE	95% CI		z	p
Intercept	-1.96	0.17	-2.31	-1.60	-10.91	< .001
RC type	0.56	0.16	0.24	0.88	3.47	.001
Relativizer	-0.07	0.19	-0.45	0.30	-0.39	1.00
RC type*relativizer	0.03	0.21	-0.38	0.46	0.17	1.00
Random Effects						
				Variance	S.D.	Correlation
Participant (slope for RC type)				0.007	0.08	
Participant (slope for relativizer)				0.0009	0.03	-0.85
Items (slope for RC type)				0.01	0.12	
Items (slope for relativizer)				0.16	0.41	-0.83

Table S2.9. Models' comparisons using Likelihood-ratio test (LRT) for quasi first-pass reading time at R3 in the contrast *Que* versus *Donde*. The asterisk in the last column represents a *p*-value below the significance level of 0.05.

Model's selection

Sampling Units	N total obs = 2688<br>N Subjects = 48; N items = 28										
**Model specification**	**Model name**	**Nested / simpler Model**	**Fixed Effects added**		**Random Effects**		**Model fit**			**LRT Test against nested**	
					**Subjects**	**Items**	**AIC**	**BIC**	**LL**	**df**	**X2**

RE only	Null	-	-		Slope for RC type + relativizer	Slope for RC type + relativizer	35844	35926	-17908		
FE main effects	Main effects	Null	RC type + relativizer		Slope for RC type + relativizer	Slope for RC type + relativizer	35846	35940	-17907	2	1.64
FE two-way interactions	Two-way interaction	Main effects	RC type + relativizer + RCtype*relativizer		Slope for RC type + relativizer	Slope for RC type + relativizer	35845	35946	-17906	1	2.72

Table S2.10. Results of the linear mixed-effect model for quasi first-pass reading time at R3 in the contrast *Que* versus *Donde*. The top of the table reports estimates/beta values, standard error (SE), confidence intervals (95% CI), *t*-values and levels of significance for fixed effects; the bottom of the table reports variance and standard deviation (S.D.) for the random effects, as well as their correlation.

Fixed Effects						
	Est/Beta	SE	95% CI		t	p
Intercept	293.65	15.43	263.41	323.89	19.03	< .001
RC type	24.94	12.31	0.80	49.07	2.02	.12
Relativizer	10.58	10.49	-9.98	31.15	1.00	.31
RC type*relativizer	-22.53	14.15	-50.26	5.19	-1.59	.22
Random Effects						
				Variance	S.D.	Correlation
Participant (slope for RC type)				1024.23	32.00	
Participant (slope for relativizer)				30.55	5.52	-0.99
Items (slope for RC type)				845.55	29.07	
Items (slope for relativizer)				265.29	16.28	0.62

Table S2.11. Models' comparisons using Likelihood-ratio test (LRT) for second-pass duration at R3 in the contrast *Que* versus *Donde*. The asterisk in the last column represents a *p*-value below the significance level of 0.05

Model's selection

Sampling Units	N total obs = 2688

	N Subjects = 48; N items = 28										
**Model specification**	**Model name**	**Nested / simpler Model**	**Fixed Effects added**		**Random Effects**		**Model fit**			**LRT Test against nested**	
					**Subjects**	**Items**	**AIC**	**BIC**	**LL**	**df**	**X2**
RE only	Null	-	-		Slope for RC type + relativizer	Slope for RC type + relativizer	36835	36918	-18404		
FE main effects	Main effects	Null	RC type + relativizer		Slope for RC type + relativizer	Slope for RC type + relativizer	36829	36924	-18399	2	9.93*
FE two-way interactions	Two-way interaction	Main effects	RC type + relativizer + RCtype*relativizer		Slope for RC type + relativizer	Slope for RC type + relativizer	36881	36982	-18424	1	0

Table S2.12. Results of the linear mixed-effect model for second-pass duration at R3 in the contrast *Que* versus *Donde*. The top of the table reports estimates/beta values, standard error (SE), confidence intervals (95% CI), *t*-values and levels of significance for fixed effects; the bottom of the table reports variance and standard deviation (S.D.) for the random effects, as well as their correlation.

Fixed Effects						
	Est/Beta	SE	95% CI		t	p
Intercept	65.36	9.31	47.10	83.63	7.01	< .001
RC type	33.14	16.00	31.73	64.51	2.07	.11
Relativizer	4.53	13.87	-22.66	31.73	0.32	1.00
RC type*relativizer	-0.01	17.44	-34.20	34.18	-0.001	1.00

Random Effects			
	Variance	S.D.	Correlation
Participant (slope for RC type)	4571.2	67.61	
Participant (slope for relativizer)	1208.9	34.77	1.00
Items (slope for RC type)	244.7	15.64	
Items (slope for relativizer)	429.9	20.74	0.97

Section F.3. Models' reports for eye-movement measures at R4

Table S3.1. Models' comparisons using Likelihood-ratio test (LRT) for first fixation at R4 in the contrast *Que* versus *Donde*. The asterisk in the last column represents a *p*-value below the significance level of 0.05

Model's selection

Sampling Units	N total obs = 2688<br>N Subjects = 48; N items = 28									
**Model specification**	**Model name**	**Nested / simpler Model**	**Fixed Effects added**	**Random Effects**		**Model fit**			**LRT Test against nested**	
				**Subjects**	**Items**	**AIC**	**BIC**	**LL**	**df**	**X2**
RE only	Null	-	-	Slope for RC type + relativizer	Slope for RC type + relativizer	32860	32942	-16416		
FE main effects	Main effects	Null	RC type + relativizer	Slope for RC type + relativizer	Slope for RC type + relativizer	32861	32955	-16415	2	2.93
FE two-way interactions	Two-way interaction	Main effects	RC type + relativizer + RCtype*relativizer	Slope for RC type + relativizer	Slope for RC type + relativizer	32858	32958	-16412	1	4.70*

Table S3.2. Results of the linear mixed-effect model for first fixation at R4 in the contrast *Que* versus *Donde*. The top of the table reports estimates/beta values, standard error (SE), confidence intervals (95% CI), *t*-values and levels of significance for fixed effects; the bottom of the table reports variance and standard deviation (S.D.) for the random effects, as well as their correlation.

Fixed Effects						
	Est/Beta	SE	95% CI		t	p
Intercept	190.21	7.55	175.39	205.02	25.16	< .001
RC type	-6.82	5.86	-18.32	4.67	-1.16	.49
Relativizer	5.62	9.77	-13.52	24.77	0.57	.56
RC type*relativizer	17.61	8.11	1.70	33.52	2.17	.09
Random Effects						

	Variance	S.D.	Correlation
Participant (slope for RC type)	33.85	5.81	
Participant (slope for relativizer)	1865.97	43.19	-0.98
Items (slope for RC type)	21.38	4.62	
Items (slope for relativizer)	663.18	25.75	0.35

Table S3.3. Models' comparisons using Likelihood-ratio test (LRT) for first-fixation rate at R4 in the contrast *Que* versus *Donde*. The asterisk in the last column represents a *p*-value below the significance level of 0.05

Model's selection

Sampling Units	N total obs = 2688<br>N Subjects = 48; N items = 28										
**Model specification**	**Model name**	**Nested / simpler Model**	**Fixed Effects added**		**Random Effects**		**Model fit**			**LRT Test against nested**	
					**Subjects**	**Items**	**AIC**	**BIC**	**LL**	**df**	**X2**
RE only	Null	-	-		Slope for RC type + relativizer	Slope for RC type + relativizer	2334.0	2410.7	-1154.0		
FE main effects	Main effects	Null	RC type + relativizer		Slope for RC type + relativizer	Slope for RC type + relativizer	2337.9	2426.3	-1153.9	2	0.15
FE two-way interactions	Two-way interaction	Main effects	RC type + relativizer + RCtype*relativizer		Slope for RC type + relativizer	Slope for RC type + relativizer	2338.3	2432.7	-1153.2	1	1.53

Table S3.4. Results of the linear mixed-effect model for first-fixation rate at R4 in the contrast *Que* versus *Donde*. The top of the table reports estimates/beta values, standard error (SE), confidence intervals (95% CI), *z*-values and levels of significance for fixed effects; the bottom of the table reports variance and standard deviation (S.D.) for the random effects, as well as their correlation.

Fixed Effects					
	Est/Beta	SE	95% CI	z	p

Intercept	1.95	0.19	1.57	2.34	10.06	< .001
RC type	-0.17	0.17	-0.51	0.16	-1.01	.62
Relativizer	-0.19	0.23	-0.65	0.25	-0.84	.62
RC type*relativizer	0.28	0.22	-0.15	0.72	1.28	.59
Random Effects						
			Variance	S.D.	Correlation	
Participant (slope for RC type)			0.09	0.31		
Participant (slope for relativizer)			0.39	0.63	-1.00	
Items (slope for RC type)			0.007	0.08		
Items (slope for relativizer)			0.44	0.66	-0.99	

Table S3.5. Models' comparisons using Likelihood-ratio test (LRT) for first-pass duration at R4 in the contrast *Que* versus *Donde*. The asterisk in the last column represents a *p*-value below the significance level of 0.05

Model's selection

Sampling Units	N total obs = 2688<br>N Subjects = 48; N items = 28										
**Model specification**	**Model name**	**Nested / simpler Model**	**Fixed Effects added**	**Random Effects**		**Model fit**			**LRT Test against nested**		
				**Subjects**	**Items**	**AIC**	**BIC**	**LL**	**df**	**X2**	
RE only	Null	-	-	Slope for RC type + relativizer	Slope for RC type + relativizer	33422	33505	-16697			
FE main effects	Main effects	Null	RC type + relativizer	Slope for RC type + relativizer	Slope for RC type + relativizer	33425	33520	-16697	2	0.94	
FE two-way interactions	Two-way interaction	Main effects	RC type + relativizer + RCtype*relativizer	Slope for RC type + relativizer	Slope for RC type + relativizer	33425	33525	-16696	1	2.15	

Table S3.6. Results of the linear mixed-effect model for first-pass duration at R4 in the contrast *Que* versus *Donde*. The top of the table reports estimates/beta values, standard

error (SE), confidence intervals (95% CI), *t*-values and levels of significance for fixed effects; the bottom of the table reports variance and standard deviation (S.D.) for the random effects, as well as their correlation.

Fixed Effects						
	Est/Beta	SE	95% CI		t	p
Intercept	203.56	9.15	185.63	221.49	22.24	< .001
RC type	-5.45	6.78	-18.74	7.84	-0.80	.84
Relativizer	2.01	10.21	-18.01	22.03	0.19	.84
RC type*relativizer	13.20	9.00	-4.43	30.85	1.46	.42
Random Effects						
				Variance	S.D.	Correlation
Participant (slope for RC type)				193.45	13.90	
Participant (slope for relativizer)				1555.76	39.44	-0.11
Items (slope for RC type)				40.94	6.39	
Items (slope for relativizer)				880.89	29.68	-0.08

Table S3.7. Models' comparisons using Likelihood-ratio test (LRT) for first-pass regression at R4 in the contrast *Que* versus *Donde*. The asterisk in the last column represents a *p*-value below the significance level of 0.05

Model's selection

Sampling Units	N total obs = 2688<br>N Subjects = 48; N items = 28										
**Model specification**	**Model name**	**Nested / simpler Model**	**Fixed Effects added**		**Random Effects**		**Model fit**			**LRT Test against nested**	
					**Subjects**	**Items**	**AIC**	**BIC**	**LL**	**df**	**X2**
RE only	Null	-	-		Slope for RC type + relativizer	Slope for RC type + relativizer	1461.7	1538.3	-717.84		
FE main effects	Main effects	Null	RC type + relativizer		Slope for RC type + relativizer	Slope for RC type + relativizer	1460.1	1548.5	-715.03	2	5.61

FE two-way interactions	Two-way interaction	Main effects	RC type + relativizer + RCtype*relativizer		Slope for RC type + relativizer	Slope for RC type + relativizer	1461.6	1555.9	-714.80	1	0.46

Table S3.8. Results of the linear mixed-effect model for first-pass regression at R4 in the contrast *Que* versus *Donde*. The top of the table reports estimates/beta values, standard error (SE), confidence intervals (95% CI), *z*-values and levels of significance for fixed effects; the bottom of the table reports variance and standard deviation (S.D.) for the random effects, as well as their correlation.

Fixed Effects						
	Est/Beta	SE	95% CI		z	p
Intercept	-3.14	0.26	-3.65	-2.62	-11.95	< .001
RC type	0.34	0.27	-0.19	0.87	1.25	.41
Relativizer	0.54	0.26	0.03	1.05	2.09	.11
RC type*relativizer	-0.22	0.31	-0.84	0.39	-0.70	.48
Random Effects						
				Variance	S.D.	Correlation
Participant (slope for RC type)				0.22	0.47	
Participant (slope for relativizer)				0.02	0.15	0.18
Items (slope for RC type)				0.02	0.16	
Items (slope for relativizer)				0.10	0.32	-0.46

Table S3.9. Models' comparisons using Likelihood-ratio test (LRT) for quasi first-pass reading time at R4 in the contrast *Que* versus *Donde*. The asterisk in the last column represents a *p*-value below the significance level of 0.05.

Model's selection

Sampling Units	N total obs = 2688<br>N Subjects = 48; N items = 28										
**Model specification**	**Model name**	**Nested / simpler Model**	**Fixed Effects added**		**Random Effects**		**Model fit**			**LRT Test against nested**	
					**Subjects**	**Items**	**AIC**	**BIC**	**LL**	**df**	**X2**

RE only	Null	-	-		Slope for RC type + relativizer	Slope for RC type + relativizer	33829	33912	-16901		
FE main effects	Main effects	Null	RC type + relativizer		Slope for RC type + relativizer	Slope for RC type + relativizer	33833	33927	-16901	2	0.22
FE two-way interactions	Two-way interaction	Main effects	RC type + relativizer + RCtype*relativizer		Slope for RC type + relativizer	Slope for RC type + relativizer	33833	33933	-16899	1	2.37

Table S3.10. Results of the linear mixed-effect model for quasi first-pass reading time at R4 in the contrast *Que* versus *Donde*. The top of the table reports estimates/beta values, standard error (SE), confidence intervals (95% CI), *t*-values and levels of significance for fixed effects; the bottom of the table reports variance and standard deviation (S.D.) for the random effects, as well as their correlation.

Fixed Effects						
	Est/Beta	SE	95% CI		t	p
Intercept	212.83	9.42	194.37	231.30	22.59	< .001
RC type	-6.16	7.34	-20.55	8.22	-0.84	.80
Relativizer	-2.69	10.39	-23.07	17.67	-0.26	.80
RC type*relativizer	14.67	9.73	-4.39	33.75	1.50	.39
Random Effects						
				Variance	S.D.	Correlation
Participant (slope for RC type)				249.57	15.80	
Participant (slope for relativizer)				1524.50	39.04	-0.15
Items (slope for RC type)				37.45	6.12	
Items (slope for relativizer)				810.50	28.47	-0.39

Table S3.11. Models' comparisons using Likelihood-ratio test (LRT) for second-pass duration at R4 in the contrast *Que* versus *Donde*. The asterisk in the last column represents a *p*-value below the significance level of 0.05.

Model's selection

Sampling Units	N total obs = 2688

	N Subjects = 48; N items = 28											
**Model specification**	**Model name**	**Nested / simpler Model**	**Fixed Effects added**		**Random Effects**		**Model fit**			**LRT Test against nested**	
					**Subjects**	**Items**	**AIC**	**BIC**	**LL**	**df**	**X2**
RE only	Null	-	-		Slope for RC type + relativizer	Slope for RC type + relativizer	35867	35949	-17919		
FE main effects	Main effects	Null	RC type + relativizer		Slope for RC type + relativizer	Slope for RC type + relativizer	35870	35964	-17919	2	1.20
FE two-way interactions	Two-way interaction	Main effects	RC type + relativizer + RCtype*relativizer		Slope for RC type + relativizer	Slope for RC type + relativizer	35872	35972	-17919	1	0

Table S3.12. Results of the linear mixed-effect model for second-pass duration at R4 in the contrast *Que* versus *Donde*. The top of the table reports estimates/beta values, standard error (SE), confidence intervals (95% CI), *t*-values and levels of significance for fixed effects; the bottom of the table reports variance and standard deviation (S.D.) for the random effects, as well as their correlation.

Fixed Effects						
	Est/Beta	SE	95% CI		t	p
Intercept	30.47	8.28	14.24	46.71	3.67	< .001
RC type	11.89	12.01	-11.65	35.44	0.99	.96
Relativizer	6.51	11.18	-15.39	28.43	0.58	1.00
RC type*relativizer	-7.38	14.57	-35.94	21.17	-0.50	1.00

Random Effects			
	Variance	S.D.	Correlation
Participant (slope for RC type)	444.9	21.09	
Participant (slope for relativizer)	689.4	26.26	-1.00
Items (slope for RC type)	809.6	28.45	
Items (slope for relativizer)	128.1	11.32	-1.00

Section F.4. Models' reports for eye-movement measures at R5

Table S4.1. Models' comparisons using Likelihood-ratio test (LRT) for first fixation at R5 in the contrast *Que* versus *Donde*. The asterisk in the last column represents a *p*-value below the significance level of 0.05.

Model's selection

Sampling Units	N total obs = 2688<br>N Subjects = 48; N items = 28											
**Model specification**	**Model name**	**Nested / simpler Model**	**Fixed Effects added**		**Random Effects**		**Model fit**			**LRT Test against nested**		
					**Subjects**	**Items**	**AIC**	**BIC**	**LL**	**df**	**X2**	
RE only	Null	-	-		Slope for RC type + relativizer	Slope for RC type + relativizer	31789	31872	-15881			
FE main effects	Main effects	Null	RC type + relativizer		Slope for RC type + relativizer	Slope for RC type + relativizer	31792	31886	-15880	2	1.24	
FE two-way interactions	Two-way interaction	Main effects	RC type + relativizer + RCtype*relativizer		Slope for RC type + relativizer	Slope for RC type + relativizer	31793	31893	-15880	1	1.17	

Table S4.2. Results of the linear mixed-effect model for first fixation at R5 in the contrast *Que* versus *Donde*. The top of the table reports estimates/beta values, standard error (SE), confidence intervals (95% CI), *t*-values and levels of significance for fixed effects; the bottom of the table reports variance and standard deviation (S.D.) for the random effects, as well as their correlation.

Fixed Effects						
	Est/Beta	SE	95% CI		t	p
Intercept	188.50	7.31	174.17	202.83	25.78	< .001
RC type	-1.57	5.83	-13.00	9.87	-0.26	1.00
Relativizer	1.28	5.42	-9.35	11.91	0.23	1.00
RC type*relativizer	-7.20	6.64	-20.22	5.81	-1.08	.83
Random Effects						

	Variance	S.D.	Correlation
Participant (slope for RC type)	244.87	15.64	
Participant (slope for relativizer)	225.35	15.01	0.05
Items (slope for RC type)	193.33	13.90	
Items (slope for relativizer)	75.97	8.71	1.00

Table S4.3. Models' comparisons using Likelihood-ratio test (LRT) for first-fixation rate at R5 in the contrast *Que* versus *Donde*. The asterisk in the last column represents a *p*-value below the significance level of 0.05.

Model's selection

Sampling Units	N total obs = 2688<br>N Subjects = 48; N items = 28										
**Model specification**	**Model name**	**Nested / simpler Model**	**Fixed Effects added**		**Random Effects**		**Model fit**			**LRT Test against nested**	
					**Subjects**	**Items**	**AIC**	**BIC**	**LL**	**df**	**X2**
RE only	Null	-	-		Slope for RC type + relativizer	Slope for RC type + relativizer	2075.9	2152.6	-1025.0		
FE main effects	Main effects	Null	RC type + relativizer		Slope for RC type + relativizer	Slope for RC type + relativizer	2079.6	2168.0	-1024.8	2	0.34
FE two-way interactions	Two-way interaction	Main effects	RC type + relativizer + RCtype*relati vizer		Slope for RC type + relativizer	Slope for RC type + relativizer	2081.6	2175.9	-1024.8	1	0.006

Table S4.4. Results of the linear mixed-effect model for first-fixation rate at R5 in the contrast *Que* versus *Donde*. The top of the table reports estimates/beta values, standard error (SE), confidence intervals (95% CI), *z*-values and levels of significance for fixed effects; the bottom of the table reports variance and standard deviation (S.D.) for the random effects, as well as their correlation.

Fixed Effects					
	Est/Beta	SE	95% CI	z	p

Intercept	2.15	0.20	1.75	2.55	10.57	< .001
RC type	-0.10	0.19	-0.49	0.28	-0.54	1.00
Relativizer	-0.01	0.19	-0.39	0.36	-0.07	1.00
RC type*relativizer	0.02	0.23	-0.44	0.48	0.08	1.00
Random Effects						
				Variance	S.D.	Correlation
Participant (slope for RC type)				1.784 e- 01	0.42	
Participant (slope for relativizer)				1.177 e- 01	0.34	-0.24
Items (slope for RC type)				2.873 e- 02	0.16	
Items (slope for relativizer)				1.841 e- 05	0.004	1.00

Table S4.5. Models' comparisons using Likelihood-ratio test (LRT) for first-pass duration at R5 in the contrast *Que* versus *Donde*. The asterisk in the last column represents a *p*-value below the significance level of 0.05.

Model's selection

Sampling Units	N total obs = 2688<br>N Subjects = 48; N items = 28									
**Model specification**	**Model name**	**Nested / simpler Model**	**Fixed Effects added**	**Random Effects**		**Model fit**			**LRT Test against nested**	
				**Subjects**	**Items**	**AIC**	**BIC**	**LL**	**df**	**X2**
RE only	Null	-	-	Slope for RC type + relativizer	Slope for RC type + relativizer	34539	34622	-17256		
FE main effects	Main effects	Null	RC type + relativizer	Slope for RC type + relativizer	Slope for RC type + relativizer	34540	34635	-17254	2	2.61
FE two-way interactions	Two-way interaction	Main effects	RC type + relativizer + RCtype*relativizer	Slope for RC type + relativizer	Slope for RC type + relativizer	34540	34640	-17253	1	2.26

Table S4.6. Results of the linear mixed-effect model for first-pass duration at R5 in the contrast *Que* versus *Donde*. The top of the table reports estimates/beta values, standard

error (SE), confidence intervals (95% CI), *t*-values and levels of significance for fixed effects; the bottom of the table reports variance and standard deviation (S.D.) for the random effects, as well as their correlation.

Fixed Effects						
	Est/Beta	SE	95% CI		t	p
Intercept	223.20	13.57	196.59	249.81	16.44	< .001
RC type	10.17	8.08	-5.67	26.01	1.25	.26
Relativizer	21.90	10.13	2.04	41.75	2.16	.09
RC type*relativizer	-16.57	11.02	-38.18	5.02	-1.50	.26

Random Effects			
	Variance	S.D.	Correlation
Participant (slope for RC type)	125.51	11.20	
Participant (slope for relativizer)	1327.29	36.43	0.07
Items (slope for RC type)	55.03	7.41	
Items (slope for relativizer)	398.75	19.96	-0.41

Table S4.7. Models' comparisons using Likelihood-ratio test (LRT) for first-pass regression at R5 in the contrast *Que* versus *Donde*. The asterisk in the last column represents a *p*-value below the significance level of 0.05.

Model's selection

Sampling Units	N total obs = 2688<br>N Subjects = 48; N items = 28										
**Model specification**	**Model name**	**Nested / simpler Model**	**Fixed Effects added**	**Random Effects**		**Model fit**			**LRT Test against nested**	
				**Subjects**	**Items**	**AIC**	**BIC**	**LL**	**df**	**X2**
RE only	Null	-	-	Slope for RC type + relativizer	Slope for RC type + relativizer	1335.1	1411.7	-654.53		
FE main effects	Main effects	Null	RC type + relativizer	Slope for RC type + relativizer	Slope for RC type + relativizer	1325.9	1414.3	-647.94	2	13.17*

FE two-way interactions	Two-way interaction	Main effects	RC type + relativizer + RCtype*relativizer		Slope for RC type + relativizer	Slope for RC type + relativizer	1327.3	1421.7	-647.66	1	0.56

Table S4.8. Results of the linear mixed-effect model for first-pass regression at R5 in the contrast *Que* versus *Donde*. The top of the table reports estimates/beta values, standard error (SE), confidence intervals (95% CI), *z*-values and levels of significance for fixed effects; the bottom of the table reports variance and standard deviation (S.D.) for the random effects, as well as their correlation.

Fixed Effects						
	Est/Beta	SE	95% CI		z	p
Intercept	-3.40	0.27	-3.95	-2.85	-12.21	< .001
RC type	0.07	0.35	-0.62	0.76	0.19	.89
Relativizer	1.02	0.30	0.43	1.61	3.40	.001
RC type*relativizer	-0.29	0.39	-1.06	0.47	-0.75	.89
Random Effects						
				Variance	S.D.	Correlation
Participant (slope for RC type)				0.19	0.43	
Participant (slope for relativizer)				0.10	0.31	-0.34
Items (slope for RC type)				0.16	0.40	
Items (slope for relativizer)				0.19	0.44	0.82

Table S4.9. Models' comparisons using Likelihood-ratio test (LRT) for quasi first-pass reading time at R5 in the contrast *Que* versus *Donde*. The asterisk in the last column represents a *p*-value below the significance level of 0.05.

Model's selection

Sampling Units	N total obs = 2688<br>N Subjects = 48; N items = 28										
**Model specification**	**Model name**	**Nested / simpler Model**	**Fixed Effects added**		**Random Effects**		**Model fit**			**LRT Test against nested**	
					**Subjects**	**Items**	**AIC**	**BIC**	**LL**	**df**	**X2**

RE only	Null	-	-		Slope for RC type + relativizer	Slope for RC type + relativizer	34798	34881	-17385		
FE main effects	Main effects	Null	RC type + relativizer		Slope for RC type + relativizer	Slope for RC type + relativizer	34796	34891	-17382	2	5.56
FE two-way interactions	Two-way interaction	Main effects	RC type + relativizer + RCtype*relativizer		Slope for RC type + relativizer	Slope for RC type + relativizer	34795	34895	-17381	1	3.19

Table S4.10. Results of the linear mixed-effect model for quasi first-pass reading time at R5 in the contrast *Que* versus *Donde*. The top of the table reports estimates/beta values, standard error (SE), confidence intervals (95% CI), *t*-values and levels of significance for fixed effects; the bottom of the table reports variance and standard deviation (S.D.) for the random effects, as well as their correlation.

Fixed Effects						
	Est/Beta	SE	95% CI		t	p
Intercept	228.20	14.46	199.85	256.55	15.77	< .001
RC type	9.68	8.80	-7.57	26.93	1.10	.27
Relativizer	33.84	11.27	11.74	55.93	3.00	.008
RC type*relativizer	-20.62	11.53	-43.23	1.98	-1.78	.14
Random Effects						
				Variance	S.D.	Correlation
Participant (slope for RC type)				300.1	17.32	
Participant (slope for relativizer)				1604.0	40.05	-0.36
Items (slope for RC type)				131.9	11.48	
Items (slope for relativizer)				760.3	27.57	-0.33

Table S4.11. Models' comparisons using Likelihood-ratio test (LRT) for second-pass duration at R5 in the contrast *Que* versus *Donde*. The asterisk in the last column represents a *p*-value below the significance level of 0.05.

Model's selection

Sampling Units	N total obs = 2688

	N Subjects = 48; N items = 28										
**Model specification**	**Model name**	**Nested / simpler Model**	**Fixed Effects added**		**Random Effects**		**Model fit**			**LRT Test against nested**	
					**Subjects**	**Items**	**AIC**	**BIC**	**LL**	**df**	**X2**
RE only	Null	-	-		Slope for RC type + relativizer	Slope for RC type + relativizer	36345	36428	-18159		
FE main effects	Main effects	Null	RC type + relativizer		Slope for RC type + relativizer	Slope for RC type + relativizer	36340	36435	-18154	2	9.06*
FE two-way interactions	Two-way interaction	Main effects	RC type + relativizer + RCtype*relativizer		Slope for RC type + relativizer	Slope for RC type + relativizer	36342	36442	-18154	1	0.02

Table S4.12. Results of the linear mixed-effect model for second-pass duration at R5 in the contrast *Que* versus *Donde*. The top of the table reports estimates/beta values, standard error (SE), confidence intervals (95% CI), *t*-values and levels of significance for fixed effects; the bottom of the table reports variance and standard deviation (S.D.) for the random effects, as well as their correlation.

Fixed Effects						
	Est/Beta	SE	95% CI		t	p
Intercept	27.83	8.05	12.05	43.61	3.45	.002
RC type	-0.90	11.86	-24.15	22.35	-0.07	1.00
Relativizer	31.49	13.05	5.90	57.08	2.41	.04
RC type*relativizer	2.24	15.90	-28.91	33.41	0.14	1.00

Random Effects			
	Variance	S.D.	Correlation
Participant (slope for RC type)	573.59	23.95	
Participant (slope for relativizer)	1587.91	39.84	0.82
Items (slope for RC type)	67.38	8.20	
Items (slope for relativizer)	309.32	17.58	0.56

Section G. Supplementary material for the eye-movement measure total reading time of R4, R5 and R6 together

Section G.1. Total reading time for the contrast *Que* vs. *El cual*

Table S1.1. Models' comparisons using Likelihood-ratio test (LRT) for total reading time of R4, R5 and R6 in the contrast *Que* versus *El cual*. The asterisk in the last column represents a *p*-value below the significance level of 0.05.

Model's selection

Sampling Units	N total obs = 8064<br>N Subjects = 48; N items = 28										
**Model specification**	**Model name**	**Nested / simpler Model**	**Fixed Effects added**		**Random Effects**		**Model fit**			**LRT Test against nested**	
					**Subjects**	**Items**	**AIC**	**BIC**	**LL**	**df**	**X2**
RE only	Null	-	-		Slope for RC type + relativizer	Slope for RC type + relativizer	118439	118537	-59206		
FE main effects	Main effects	Null	RC type + relativizer		Slope for RC type + relativizer	Slope for RC type + relativizer	118465	118577	-59216	2	0
FE two-way interactions	Two-way interaction	Main effects	RC type + relativizer + RCtype*relativizer		Slope for RC type + relativizer	Slope for RC type + relativizer	118467	118586	-59216	1	0.19

Table S1.2. Results of the linear mixed-effect model for total reading time of R4, R5 and R6 in the contrast *Que* versus *El cual*. The top of the table reports estimates/beta values, standard error (SE), confidence intervals (95% CI), *t*-values and levels of significance for fixed effects; the bottom of the table reports variance and standard deviation (S.D.) for the random effects, as well as their correlation.

Fixed Effects					
	Est/Beta	SE	95% CI	t	p

Intercept	556.45	29.84	16.40	614.95	18.64	< .001
RC type	-35.98	15.58	-66.53	-5.43	-2.3	.06
Relativizer	-31.17	14.83	-60.23	-2.10	-2.1	.07
RC type*relativizer	-7.27	16.40	-39.42	24.87	-0.44	.65
Random Effects						
				Variance	S.D.	Correlation
Participant (slope for RC type)				57.35	7.57	
Participant (slope for relativizer)				227.89	15.09	1.00
Items (slope for RC type)				3001.81	54.78	
Items (slope for relativizer)				2257.38	47.51	0.60

Section G.2. Total reading time for the contrast *Que* vs. *Quien*

Table S2.1. Models' comparisons using Likelihood-ratio test (LRT) for total reading time of R4, R5 and R6 in the contrast *Que* versus *Quien*. The asterisk in the last column represents a *p*-value below the significance level of 0.05.

Model's selection

Sampling Units	N total obs = 8064<br>N Subjects = 48; N items = 28									
**Model specification**	**Model name**	**Nested / simpler Model**	**Fixed Effects added**	**Random Effects**		**Model fit**			**LRT Test against nested**	
				**Subjects**	**Items**	**AIC**	**BIC**	**LL**	**df**	**X2**
RE only	Null	-	-	Slope for RC type + relativizer	Slope for RC type + relativizer	120188	120286	-60080		
FE main effects	Main effects	Null	RC type + relativizer	Slope for RC type + relativizer	Slope for RC type + relativizer	120176	120287	-60072	2	16.92*
FE two-way interactions	Two-way interaction	Main effects	RC type + relativizer + RCtype*relativizer	Slope for RC type + relativizer	Slope for RC type + relativizer	120175	120294	-60071	1	2.46

Table S2.2. Results of the linear mixed-effect model for total reading time of R4, R5 and R6 in the contrast *Que* versus *Quien*. The top of the table reports estimates/beta values, standard error (SE), confidence intervals (95% CI), *t*-values and levels of significance for fixed effects; the bottom of the table reports variance and standard deviation (S.D.) for the random effects, as well as their correlation.

Fixed Effects						
	Est/Beta	SE	95% CI		t	p
Intercept	542.50	34.03	475.81	609.20	15.94	< .001
RC type	-54.39	12.95	-79.78	-28.99	-4.19	< .001
Relativizer	-8.00	13.12	-33.72	17.71	-0.61	.54
RC type*relativizer	28.65	18.24	-7.10	64.41	1.57	.23

Random Effects			
	Variance	S.D.	Correlation
Participant (slope for RC type)	3.427 e+00	1.85	
Participant (slope for relativizer)	2.102 e+02	14.94	1.00
Items (slope for RC type)	3.906 e+01	6.24	
Items (slope for relativizer)	3.743 e+01	6.11	-1.00

Section G.3. Total reading time for the contrast *Que* vs. *Donde*

Table S3.1. Models' comparisons using Likelihood-ratio test (LRT) for total reading time of R4, R5 and R6 in the contrast *Que* versus *Donde*. The asterisk in the last column represents a *p*-value below the significance level of 0.05.

Model's selection

Sampling Units	N total obs = 8064<br>N Subjects = 48; N items = 28										
**Model specification**	**Model name**	**Nested / simpler Model**	**Fixed Effects added**	**Random Effects**		**Model fit**			**LRT Test against nested**	
				**Subjects**	**Items**	**AIC**	**BIC**	**LL**	**df**	**X2**
RE only	Null	-	-	Slope for RC type + relativizer	Slope for RC type + relativizer	118903	119000	-59437		

FE main effects	Main effects	Null	RC type + relativizer		Slope for RC type + relativizer	Slope for RC type + relativizer	118900	119012	-59434	2	6.24*
FE two-way interactions	Two-way interaction	Main effects	RC type + relativizer + RCtype*relativizer		Slope for RC type + relativizer	Slope for RC type + relativizer	118902	119020	-59434	1	0.67

Table S3.2. Results of the linear mixed-effect model for total reading time of R4, R5 and R6 in the contrast *Que* versus *Donde*. The top of the table reports estimates/beta values, standard error (SE), confidence intervals (95% CI), *t*-values and levels of significance for fixed effects; the bottom of the table reports variance and standard deviation (S.D.) for the random effects, as well as their correlation.

Fixed Effects						
	Est/Beta	SE	95% CI		t	p
Intercept	510.80	32.50	447.10	574.50	15.71	< .001
RC type	-5.93	12.41	-30.27	18.39	-0.47	.82
Relativizer	25.53	12.08	1.84	49.23	2.11	.10
RC type*relativizer	-13.88	16.95	-47.11	19.35	-0.81	.82

Random Effects			
	Variance	S.D.	Correlation
Participant (slope for RC type)	2.951 e+02	17.18	
Participant (slope for relativizer)	1.186 e+02	10.89	-1.00
Items (slope for RC type)	1.194 e+02	10.92	
Items (slope for relativizer)	1.427 e+00	1.19	1.00
